# Targeted strategies for the management of wildlife diseases: the case of brucellosis in Alpine ibex

**DOI:** 10.1186/s13567-021-00984-0

**Published:** 2021-09-14

**Authors:** Sébastien Lambert, Anne Thébault, Sophie Rossi, Pascal Marchand, Elodie Petit, Carole Toïgo, Emmanuelle Gilot-Fromont

**Affiliations:** 1 Laboratoire de Biométrie et Biologie Évolutive UMR 5558, CNRS, Université Lyon 1, Université de Lyon, Villeurbanne, France; 2 Direction de l’évaluation des Risques, Agence Nationale de Sécurité Sanitaire, de l’Alimentation, de l’Environnement et du Travail (Anses), Maisons-Alfort, France; 3 Unité Sanitaire de La Faune, Office Français de la Biodiversité (OFB), Gap, France; 4 Unité Ongulés Sauvages, Office Français de la Biodiversité (OFB), Juvignac, France; 5 Unité Sanitaire de La Faune, Office Français de la Biodiversité (OFB), Sévrier, France; 6 Unité Ongulés Sauvages, Office Français de La Biodiversité (OFB), Gières, France; 7 Laboratoire de Biométrie et Biologie Évolutive UMR 5558, CNRS, VetAgro Sup, Université de Lyon, Villeurbanne, France; 8 Present Address: Department of Pathobiology and Population Sciences, Royal Veterinary College, University of London, Hatfield, UK

**Keywords:** Wildlife disease, disease management, metapopulation, heterogeneity, mathematical modelling, capture, vaccination, culling, targeting, test-and-remove

## Abstract

The management of infectious diseases in wildlife reservoirs is challenging and faces several limitations. However, detailed knowledge of host–pathogen systems often reveal heterogeneity among the hosts’ contribution to transmission. Management strategies targeting specific classes of individuals and/or areas, having a particular role in transmission, could be more effective and more acceptable than population-wide interventions. In the wild population of Alpine ibex (*Capra ibex*—a protected species) of the Bargy massif (French Alps), females transmit brucellosis (*Brucella melitensis*) infection in ~90% of cases, and most transmissions occur in the central spatial units (“core area”). Therefore, we expanded an individual-based model, developed in a previous study, to test whether strategies targeting females or the core area, or both, would be more effective. We simulated the relative efficacy of realistic strategies for the studied population, combining test-and-remove (euthanasia of captured animals with seropositive test results) and partial culling of unmarked animals. Targeting females or the core area was more effective than untargeted management options, and strategies targeting both were even more effective. Interestingly, the number of ibex euthanized and culled in targeted strategies were lower than in untargeted ones, thus decreasing the conservation costs while increasing the sanitary benefits. Although there was no silver bullet for the management of brucellosis in the studied population, targeted strategies offered a wide range of promising refinements to classical sanitary measures. We therefore encourage to look for heterogeneity in other wildlife diseases and to evaluate potential strategies for improving management in terms of efficacy but also acceptability.

**Supplementary Information:**

The online version contains supplementary material available at 10.1186/s13567-021-00984-0.

## Introduction

Wildlife populations can act as reservoirs of multi-host infections shared with domestic livestock, such as bovine tuberculosis or brucellosis, that have strong impacts on human or animal health, and detrimental consequences on human activities [1]. This role of reservoir may become apparent when disease management programs entail massive decrease of incidence and prevalence in domestic animals, up to a level when the eradication efforts are hampered by the wildlife reservoir, as observed for example for bovine tuberculosis in several areas in the world [2]. Wildlife reservoirs can also trigger a re-emergence of the infection after eradication in the domestic compartment was obtained, and thus jeopardize the infection-free status. In this context of (re)-emergence of zoonotic infectious diseases caused by infections with wildlife reservoirs [3], the use of disease management strategies in wildlife has been increasing in the last decades [4].

The management of infectious diseases in wildlife is yet particularly challenging compared to their counterpart in domestic animals. Numerous tools are available to mitigate or eradicate infectious diseases in wildlife, but they all face important limitations. Among them, logistical and financial constraints, preservation of genetic variability of the host populations, impact on the ecosystem beyond the host–pathogen system, or ethical and acceptability issues, are just a few examples [5–8]. Moreover, the efficacy of management strategies is highly dependent on the host ecology and the transmission dynamics of infectious diseases in wildlife, which are often poorly understood. In addition, unexpected ecological interactions between management interventions and the host–pathogen system can lead to counterintuitive outcomes and reduce management efficacy [9–11].

As a result, managers and decision-makers are facing high uncertainties for the choice of a strategy when confronted with wildlife infectious diseases. For instance, various strategies of culling are often used to manage wildlife diseases, but their acceptability is regularly questioned by their unguaranteed efficacy and their costs for biodiversity and ecosystems conservation [12].

Mathematical modelling is a useful tool to help decision-makers by providing a better understanding of disease dynamics and evaluating the consequences of disease management strategies in wildlife [13–15]. One major advantage of mathematical models is the possibility to compare the relative efficacy of several strategies through simulations, which is often impractical in real life conditions [16]. However, to evaluate the effectiveness of management as accurately as possible, a deep knowledge of the host–pathogen system is required, with a detailed understanding of infection dynamics and the integration of biological characteristics that determine the persistence of infectious agent. Behavioral ecology and demography of the host population are namely key factors in the host–pathogen dynamics, that have to be identified for an integrative management.

Interestingly, detailed knowledge on wild host–pathogen systems often reveals spatial and individual heterogeneity of the hosts’ contributions to the transmission dynamics [17, 18]. Management strategies, including culling, targeting the areas or the individuals responsible for most transmissions could therefore represent an opportunity for wildlife disease management schemes which are more cost-effective and also more acceptable for wildlife conservation [19, 20]. Such targeted interventions could be used not only for reducing intra-specific transmission within the wild reservoir host, but also for reducing inter-specific transmission between the wild population and the population to protect (e.g., domestic livestock).

For instance, instead of applying a unique treatment to the entire population, specific subpopulations can be targeted depending on their spatial situation and their role in disease spread [21–23]. Likewise, targeting specific individuals based on certain traits associated with higher risk of transmission such as age, sex or dominance status could also provide better results and higher acceptability than population-wide management [24–26]. Thus, in addition to common population-wide disease management strategies, it is also possible to compare the relative efficacy of more specific targeted strategies using mathematical models [27].

In the present study, we focused on the management of *Brucella melitensis* infection in the wild population of Alpine ibex (*Capra ibex*) of the Bargy massif (French Alps). This is an example of multi-host infection shared between wildlife, livestock and humans, that causes economic and public health issues [28]. In this case study, the population of ibex acted as a reservoir of brucellosis, which was revealed when the infection reemerged in livestock and humans in 2012, while France has been officially free of brucellosis in domestic ruminants for several years [28, 29]. As it was the first reported case of brucellosis maintenance in this species, the absence of detailed knowledge on this particular host–pathogen system led the French authorities to implement a capture–recapture and spatial field monitoring, while attempting several management strategies classically applied in wildlife populations. Extensive culling operations, selective culling operations (removing only the seropositive individuals, “test-and-remove”) or combinations of both were successively implemented in this population to mitigate the risk of brucellosis transmission to livestock and humans. However, such strategies raised ethical and population conservation issues, especially as ibex populations (including the studied population) have been restored in the Alps during the last decades and the species is now protected in France.

Given this context with strong public health and economic issues on the one hand, and ethical and population conservation concerns on the other hand, it is of the upmost importance to find optimal disease management strategies. The current objective of the Authorities is to control *Brucella melitensis* infection by reducing prevalence in ibex, so as to reduce the probability of transmission to livestock and increase the probability that *B. melitensis* would fade-out, without threatening the conservation of the ibex population [30].

Targeted management strategies could be relevant in this case by maximizing the effectiveness while minimizing the costs of field operations and the negative impact of interventions on the ibex population. Indeed, in-depth knowledge of the host–pathogen system is now available, which notably revealed a strong spatial structure and heterogeneity for brucellosis transmission in the studied population [31–33]. More specifically, it has been highlighted that females transmit the infection in ~90% of cases [33] and that they are spatially structured in five subpopulations with nearly no female movements between them [31]. These subpopulations exhibit contrasted seroprevalence levels [31]. The subpopulations with the highest seroprevalence and abundance, that occupy a central position in the massif, are hotspots of transmission and act as sources for the subpopulations in the periphery, in particular due to movements of males between female subpopulations [33]. Based on knowledge accumulated on this brucellosis-ibex system since 2012, it is now possible to evaluate the relative efficacy of different management strategies, and to compare them with their counterparts targeting specific individuals or subpopulations.

To achieve this goal, we expanded the model developed in a previous study [33] to simulate the future dynamics of the population, with and without disease management, so as to evaluate the relative efficacy of various management strategies. Our first objective was to evaluate and compare the following strategies: (i) “do nothing”, without any management actions, which corresponds to the reference to which the other strategies will be compared to, (ii) serological testing of live individuals followed by removal (here, mainly euthanasia) of seropositive individuals (“test-and-remove”), and (iii) test-and-remove of live individuals combined to culling of others without testing. The simulated strategies representing possible future management options were elaborated in close connection with the ongoing field monitoring and management in order to simulate realistic schemes and parameters. Given the strong spatial structure and heterogeneity for brucellosis transmission highlighted in the studied population, our second objective was to evaluate the above disease management strategies, but targeting specific gender, specific subpopulations, or both. We formulated the following predictions:

- First, management strategies targeting the core area of the massif, i.e., the subpopulations at the center of the massif, where seroprevalence was ~3 to 7 times higher than in the periphery [31] should be more effective in reducing transmission than management strategies applied over the whole massif.
- Second, targeting specifically females should be more effective in reducing transmission than management applied to both sexes, because females play a predominant role in the transmission of brucellosis, being responsible for ~90% of transmission [33].
- Finally, combining both approaches in a “multilevel” strategy (targeting females from the core area) should be even more effective for reducing transmission than either one alone.

All strategies were compared in terms of efficacy (change in seroprevalence and probability of extinction) and demographic impact (population size and number of individuals removed or culled).

## Materials and methods

### Study site and population

We focused on the Alpine ibex population of the Bargy massif in the French Alps (46°N, 6.28°E; elevation: 1500–2438 m; area: *ca.* 7000 ha). Brucellosis was detected in the ibex population in 2012, with a seroprevalence over 38% [29, 34]. Several culling operations were conducted since then in an attempt to try to eradicate brucellosis, leading to drastic decrease in population size. More precisely, the pre-breeding (excluding newborns) population size was 567 individuals (95% confidence interval [487–660]) in 2013, and decreased by approximately half after massive culling operations conducted in 2013 [31]. It then decreased again in 2015 after a massive test-and-remove operation with 38 euthanized seropositive individuals out of 125 captured ibex, combined with the indiscriminate culling of 70 unmarked ibex. After 2015, lighter test-and-remove operations were implemented and the population size stabilized (around 374 [326–435] individuals in 2018; C. Toïgo, unpubl. data). In parallel, the seroprevalence of brucellosis decreased in the population: in females of the core area, the seroprevalence decreased from 51% in 2013 to 21% in 2018 [35]. Therefore, we placed our study in the context where population abundance and level of seroprevalence had already been affected by management strategies that took place between 2012 and 2018 and simulated the period 2019–2028 under various scenarios.

### Individual-based model

We expanded the stochastic individual-based SEIR (Susceptible, Exposed, Infectious, Recovered) model developed in a previous study to represent the brucellosis dynamics in the studied ibex population [33].

The full description of the model and the complete system of mathematical equations are provided in Additional file 1. The model considered ibex demography, the population structure into five socio-spatial units, transmission of infection within- and between-unit, and management strategies, which are all briefly described below.

Population dynamics were shaped to reproduce a logistic population growth, where the population size stabilises around the carrying capacity in the absence of management interventions, using density-dependent responses of population parameters. However, the massive culling operations of 2013 and 2015 did not induce any increase in female reproductive success to date, despite the drop in population size (C. Toïgo, unpubl. data). This may be explained by a delay of several years on density-dependent responses [36], although the exact duration of this delay remains uncertain. To take this uncertainty into account in our model, we considered that the density-dependent regulation of population parameters could relax from the 1^st^ year (2019) up to the year after our simulations (2029), the latter case corresponding to the absence of increase in reproductive output up to 2028.

Because females in the studied population are structured into five socio-spatial units as revealed by GPS data [31], we relied on a spatial metapopulation model where all individuals living in the same socio-spatial unit constituted a subpopulation, each subpopulation being characterised by its own relative carrying capacity, defined as a proportion of the carrying capacity of the whole population. The five subpopulations constituted the overall metapopulation [33]. In the model, only males were able to move temporarily between subpopulations, with probabilities determined from GPS data (movements mostly occurred during the mating period [33]).

Within-unit transmission of brucellosis occurred via four transmission routes demonstrated in domestic ruminants and suspected in ibex based on bacteriological data: horizontal transmission after abortion (first pregnancy post-infection) or parturition when *Brucella* is shed in genital fluids, venereal transmission, congenital transmission and pseudo-vertical transmission [32, 37]. Horizontal transmission after abortion or parturition was the most important transmission route, followed by the vertical and venereal transmission routes [33]. We assumed contacts within units were density-dependent for horizontal transmission of *Brucella* through infectious abortions or births, and frequency-dependent for venereal transmission [33]. To depict between-unit transmission, we used a mechanistic model that explicitly integrated movements of males, representing opportunities of contacts and transmission between individuals from different subpopulations [33].

Susceptible (S) individuals that acquired infection became exposed (E) during the incubation period, then became infectious (I), i.e., seropositive, actively infected and shedding the bacteria. As the probability of active infection decreases with increasing age in seropositive individuals [32], we assumed that infectious ibex became recovered (R), i.e. still seropositive but not actively infected and not shedding the bacteria, after an average duration of infectious period based on bacteriological data [32, 33].

Finally, test-and-remove and culling operations were integrated in the model. The model was attributed an objective level for the total number of individuals to be captured or culled annually (see below). Each year, this total number was randomly distributed among each targeted sex-class and socio-spatial unit, before sampling individuals at random inside each category. For test-and-remove during captures, seropositive individuals were removed (euthanized), whereas seronegative ones were marked and released. The sensitivity and specificity of serological tests were assumed to be 95% and 100%, respectively [33, 36]. In each case, if the number of individuals to be captured or culled in a given socio-spatial unit and sex-class was greater than the number available, management action was applied only to the available individuals in the socio-spatial unit and sex-class under consideration. As a result, the number of treated animals could be below the objective level, by lack of available individuals (representing also the field difficulties that could occur when trying to implement a given strategy).

### Management strategies

**Figure 1:**
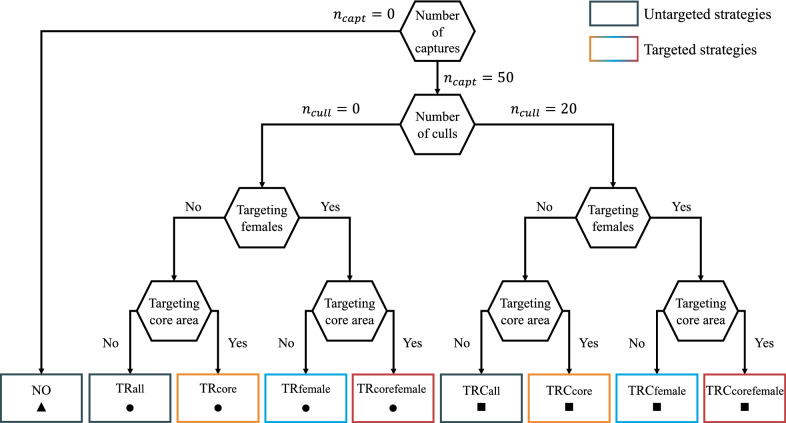
**Diagram of management strategies.** Management strategies can be distinguished based on the number of captures﻿ ($${n}_{capt}$$﻿) for test-and-remove operations (TR, circles), the number of culls ($${n}_{cull})$$ when test-and-remove is combined with the culling of unmarked (never captured) individuals (TRC, squares), and whether females and the core area are targeted. Do nothing (NO, triangle) and TR(C)all are untargeted strategies, TR(C)core is targeted towards the core area of the massif, TR(C)female is targeted towards females, TR(C)corefemale is targeted towards both females and the core area.

We simulated several management strategies, classically applied in wildlife populations and that were used in the past or are under consideration in the studied population. In addition, we refined these strategies for our case study by targeting specific genders or specific socio-spatial units depending on their role in transmission (Figure 1).

To respond to our first objective, we compared two scenarios with untargeted management strategies to a reference scenario without any management, called “do nothing” (“NO”). The first untargeted strategy was called test-and-remove (“TRall”), i.e., serological testing of captured individuals followed by removal (euthanasia) of seropositive individuals among them. In our scenarios, only individuals that were never captured before (i.e., unmarked) were targeted for capture, but this could be refined in the future. Ibex < 2 years of age were not targeted, as those are seldom captured in the studied population. The maximum target level for the number of captures was 50 individuals each year, which was the level achieved in the last years in the population among an overall population of around 300 individuals. This level was considered to be the maximal feasible level for the implementation of field operations.

The second untargeted strategy was to combine TR with the culling of unmarked individuals without testing (“TRCall”). The idea behind this strategy is that unmarked individuals are expected to have a higher risk of being seropositive than marked individuals (which were seronegative when released). The combination of TR with culling of unmarked individuals should therefore decrease the number of infected individuals more effectively, while maintaining a proportion of marked individuals that are not submitted to culling (and supposedly healthy). Only newborns were not targeted by culling for ethical reasons. The maximum objective level for the number of unmarked individuals culled was limited to 20 individuals each year.

To respond to our second objective, we compared three targeted versions of the TR and TRC strategies to their untargeted counterparts (“TR(C)all”): (i) TR(C) targeted towards the socio-spatial units of the core area of the massif (“TR(C)core”), (ii) TR(C) targeted towards females (“TR(C)female”) and (iii) TR(C) targeted towards both levels, i.e., females and the core area (“TR(C)corefemale”). The rationale behind these versions was that most transmission occurs in the core area, and that females play a predominant role in the transmission of brucellosis [33]. Therefore, targeting these specific areas and gender should be more effective than population-wide interventions.

### Initial conditions and model outputs

The initial population for the first time step of the model was based on the simulated population at the last time step of the previously published model [33]. This previous model ran for 6 years (between December 1, 2012 and November 30, 2018) and was calibrated by fitting three parameters to observed data using Approximate Bayesian Computation (ABC) rejection algorithm (see [33] for details). Simulations with 1000 iterations, each iteration using a set of parameter values from the 1000 sets retained in the ABC, produced predictions in accordance with observations both qualitatively and quantitatively [33]. Therefore, we used the 1000 simulated populations at the last time step as initial conditions for our current study. The model was developed to represent the evolution of brucellosis transmission and population dynamics for 10 years with a discrete weekly time step.

Model outputs were: (i) the seroprevalence at the end of the simulations, which is a proxy of infection in the population and can be directly compared with field data; (ii) the proportion of simulations where *Brucella* was no longer present at the end of the simulation (no remaining infectious ibex), used as a probability of extinction of brucellosis in this wild reservoir, which would be a desirable goal; (iii) the final population size; and the numbers of individuals (iv) captured, (v) removed (during test-and-remove protocol), and (vi) culled (without testing) after 10 years of simulations, in order to provide useful information to approach the economic and population conservation costs and the acceptability of field operations.

### Sensitivity analysis

Parameter values were calibrated in Lambert et al. [33]. However, given the remaining uncertainty in the three parameters fitted by ABC, and the additional uncertainty around new parameters related to management interventions, we performed a sensitivity analysis of model outputs to a selected subset of 11 parameters (Table 1). Using a fractional factorial plan, with three levels per parameter: the median, minimum and maximum of their range values, we performed a global sensitivity analysis on simulated outputs, implemented in the multisensi R package [38, 39]. For each parameter, generalized sensitivity indexes GSI were calculated and were represented by Pareto plots. Details of the sensitivity analysis can be found in Additional file 2.

**Table 1:** **Definition of model parameters included in the sensitivity analysis.**

Symbol	Description (dimension)	Fixed value	Range value	References
${d}_{dens}$	Delay between the beginning of the simulations and the relaxation of the density-dependent regulation (years)		[0–10]	*
$K$	Carrying capacity of the metapopulation (individuals)		[535–591]	†
${d}_{man}$	Duration of management interventions (years)	10	[5–10]	‡
${n}_{capt}$	Objective level for the total number of individuals to be captured (per year)	50	[0–50]	‡
${n}_{cull}$	Objective level for the total number of individuals to be culled (per year)	20	[0–20]	‡
$Se$	Sensitivity of serologic tests	0.95	[0.75–1]	*; [36, 44–46]
${\nu }_{ven}$	Probability of successful venereal transmission given contact between an infectious and a susceptible host		[0.005–0.682]	†
${\beta }_{cong}$	Congenital transmission probability by in utero infection	0.05	[0–0.10]	*; [47]
${\beta }_{pseu}$	Pseudo-vertical transmission probability by milk ingestion	0.05	[0–0.10]	*
${\beta }_{IA}={\beta }_{IB}$	Per capita probability of one host coming into effective contact with one infectious abortion or birth (per week)		[0.001–0.128]	†
γ	Probability of recovery (annual)	0.16	[0.11–0.22]	§

^*^Experts knowledge.

^†^95% credible interval (Approximate Bayesian Computation); Lambert et al. [33].

^‡^Tailored to represent realistic management interventions in the study population.

^§^95% confidence interval (calibrated using field data); Lambert et al. [33].

### Comparison of management strategies

We simulated the evolution of the ibex population and of the disease dynamics for 10 years (between 2019 and 2028) for each management strategy. Each simulation ran with 1000 iterations, each iteration using a set of parameter values obtained by Approximate Bayesian Computation to reflect uncertainty in parameter values [33].

Given the uncertainty around the delay in density-dependent responses and the importance of this uncertainty on the model outputs (see “Results”), we considered three possible assumptions: (i) the “short-delay” assumption, where the density-dependent regulation of population parameters relaxed the 1^st^ year (2019) of the simulations (${d}_{dens}=0$ years); (ii) the “medium-delay” assumption, where the density-dependent regulation of population parameters relaxed the 6^th^ year (2024) of the simulations (${d}_{dens}=5$ years); and (iii) the “long-delay” assumption, where the density-dependent regulation of population parameters would relax the year after the end of our simulations, corresponding to the hypothesis of no increase reproductivity up to 2028 (${d}_{dens}=10$ years). The results of each management strategy were therefore simulated under each of these three assumptions (Additional file 3).

In order to compare the effects of each strategy, we used Mann–Whitney (MW) tests to compare the distributions of the seroprevalence outputs between pairs of scenarios, and Chi-squared (χ^2^) tests to compare the proportions of simulations where *Brucella* was extinct at the end of the simulation. p-values under 0.05 were considered significant.

Finally, an additional untargeted scenario (test-and-vaccinate-or-remove) was explored to assess the efficacy of vaccination, although the use of the only commercially available vaccine (*B. melitensis* Rev.1 vaccine) *in natura* faces many limitations [30, 40]. This scenario is presented in Additional file 4.

## Results

### Sensitivity analysis

Among the 11 parameters included in the sensitivity analysis (Table 1), seven parameters had generalized sensitivity indices over 0.05 and were the most influential on the six outputs considered (Table 2 and Additional file 2). Epidemiological outputs (seroprevalence and probability of *Brucella* extinction) were mainly influenced by the per-capita probability of one host coming into effective contact with one infectious abortion or birth (${\beta }_{IA}={\beta }_{IB}$), whereas the delay in density-dependent responses (${d}_{dens}$) accounted for most of the variation in the population size. Finally, variations in management outputs (numbers of individuals captured, removed and culled) depended mainly on management parameters (duration of management interventions, objective level for the number of individuals to be captured or culled annually), except for ${\beta }_{IA}={\beta }_{IB}$ being the second most influential parameter for the number of individuals removed during captures (Table 2). The results of the sensitivity analysis were similar when considering untargeted or targeted management scenarios (Additional file 2).

**Table 2:** **Generalized sensitivity indices for each parameter and each simulated output in the untargeted scenarios.**

Outputs	Parameters						
	${\beta }_{IA}={\beta }_{IB}$	${n}_{capt}$	${d}_{dens}$	${n}_{cull}$	${d}_{man}$	${\nu }_{ven}$	$K$
Seroprevalence	**0.86**	0.02	0.12	0.01	0.01	0.09	0.01
Probability of *Brucella* extinction	**0.66**	0.07	0.05	0.02	0.01	**0.29**	0.02
Population size	0.04	0.03	**0.69**	0.10	0.03	0.01	**0.23**
Number (over 10 years)							
Captured	0.01	**0.81**	0.07	0.01	**0.23**	0.01	0.01
Removed	**0.31**	**0.62**	0.03	0.03	0.18	0.04	0.02
Culled	0.01	0.04	0.05	**0.82**	**0.21**	0.01	0.01
All outputs	0.28	0.28	0.18	0.18	0.12	0.05	0.05

Only the results for the seven main parameters (generalized sensitivity indices over 0.05 when considering all simulated outputs) are presented. Outputs were: (i) the seroprevalence at the end of the simulations (median and variance); (ii) the proportion of simulations where *Brucella* was extinct at the end of the simulation; (iii) the population size at the end of the simulations (median and variance); and the numbers of individuals (iv) captured, (v) removed (during test-and-remove protocol), and (vi) culled (without testing) after 10 years of simulations (median and variance). For each output, one or two parameters were clearly identified as key parameters with global sensitivity indices over 0.20 (in bold). Parameter definitions are provided in Table 1.

Based on these results, we compared management strategies in the following sections using the maximum possible values for management parameters. Remaining uncertainty around the three parameters (${\beta }_{IA}={\beta }_{IB}$, ${\upsilon }_{ven}$ and $K$) fitted to observed data in our previous study was accounted for by simulating 1000 iterations, each iteration using a set of parameter values retained in the ABC. Finally, to account for the uncertainty and the influence of the delay in density-dependent responses, we considered three different assumptions for this parameter (see “Materials and methods”).

### Management interventions vs do nothing

**Figure 2:**
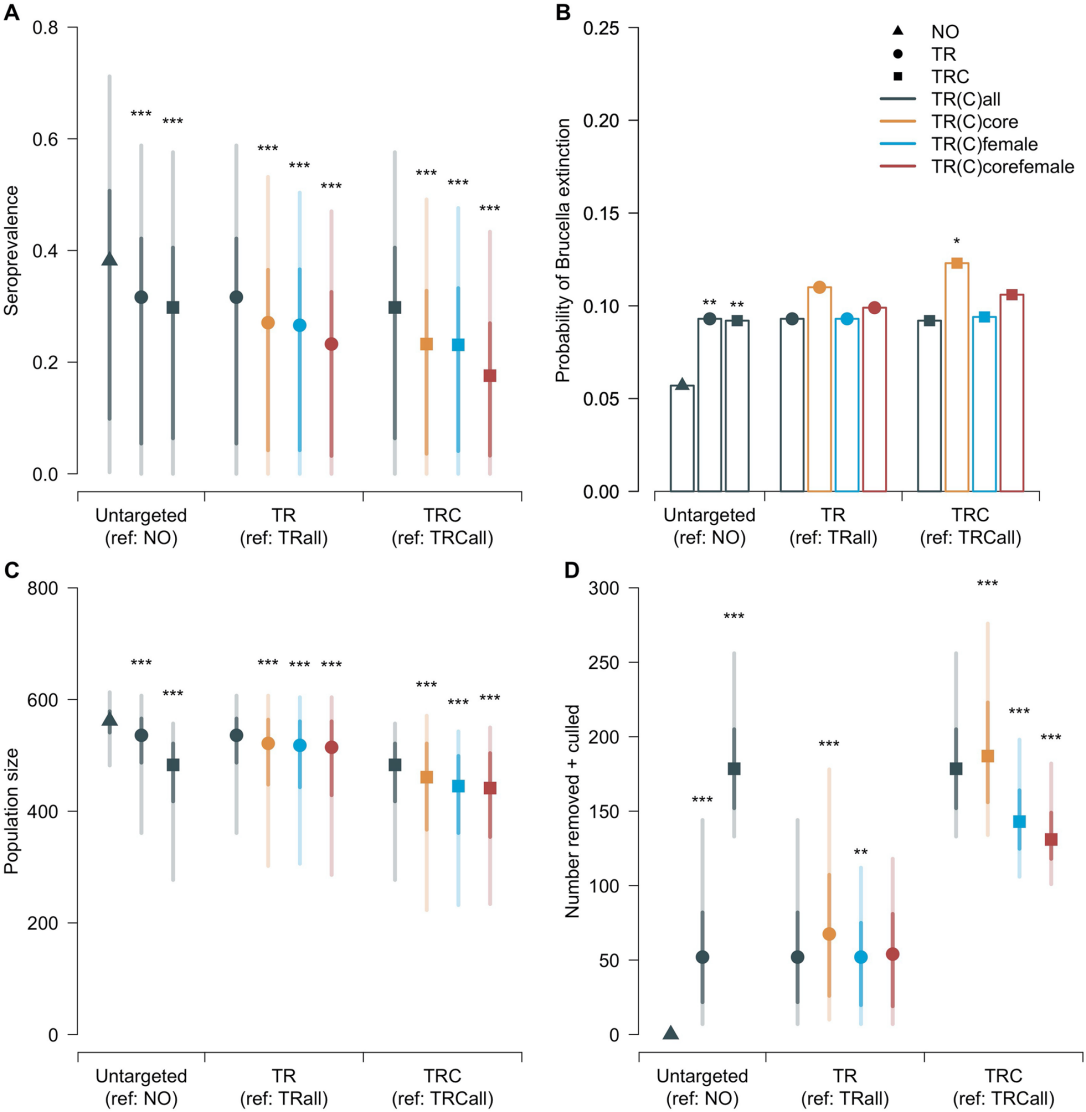
**Results of untargeted and targeted management scenarios. A **Simulated seroprevalence at the end of the simulations; **B** Proportion of simulations where *Brucella melitensis* was no longer persistent at the end of the simulations; **C** Population size at the end of the simulations; **D** total number of individuals removed and culled over the 10 years of simulations. Results obtained under the short-delay in density-dependence assumption (see Additional file 3 for the medium-delay and long-delay in density-dependence assumptions). NO: do nothing (triangle), TR: test-and-remove (points), TRC: TR combined with the culling of unmarked individuals (squares), TR(C)all: untargeted (grey), TR(C)core: targeted towards the core area (orange), TR(C)female: targeted towards females (blue), TR(C)corefemale: targeted towards both females and the core area (dark red). Except for the probability of *Brucella* extinction (single value), central points indicate the median, with 95% and 50% credible intervals indicated by light and dark shaded bars, respectively. Stars above bars indicate the p-values (“***”: *p* < 0.001; “**”: *p* < 0.01; “*”: *p* < 0.05) of the Chi-squared (**B**) or Mann–Whitney (**A, C**–**D**) tests comparing the result of a given strategy with its reference (indicated in the x-axis). All scenarios (except NO) had an objective of 50 individuals captured each year, and TRC had an additional objective of 20 unmarked individuals culled each year.

In all scenarios, the longer the delay in density-dependent responses, the lower the seroprevalence at the end of the simulations, the greater the proportion of simulations where brucellosis faded out, and the lower the population size at the end of the simulations (Figure 2 and Additional file 3).

The probability of *B. melitensis* extinction in the reference scenario “do nothing” (NO) was very low, from 5.7% (${d}_{dens}=0$ years) to 7.5% (${d}_{dens}=10$ years). All management strategies (TR/TRC, targeted/untargeted) showed increased sanitary benefits (significantly higher probabilities of extinction and significantly lower seroprevalence) associated with increased population costs (significantly lower population sizes) compared to the reference scenario NO (Figure 2 and Additional file 3).

### Test-and-remove alone or in combination with culling

The total number of individuals captured and removed was significantly lower for TRC compared to their TR counterparts because culling unmarked individuals each year reduced the number of unmarked individuals available for captures (Table 3 and Additional file 3). TRC was nonetheless associated with increased or similar sanitary benefits compared to TR (non-significant differences or significantly lower seroprevalence and significantly higher probabilities of extinction depending on the assumption; Table 3). However, the increased sanitary benefits for TRC compared to TR were also always associated with a significant increase of the sum of the number of individuals removed (during test-and-remove protocol) and the number of individuals culled (without testing) because of the additional culling, resulting in a significantly lower population size at the end of the simulations (Figure 2 and Additional file 3).

**Table 3:** **Total number of individuals managed after 10 consecutive years of untargeted and targeted management scenarios.**

	TR scenarios			
	TRall (reference)	TRcore	TRfemale	TRcorefemale
Number (over 10 years)				
Captured	310 [273–350]	296 [255–329] $\downarrow$ (*p* < 0.001)	207 [168–246] $\downarrow$ (*p* < 0.001)	184 [141–224] $\downarrow$ (*p* < 0.001) $\downarrow$ (*p* < 0.001)^a^ $\downarrow$ (*p* < 0.001)^b^
Removed	52 [7–144]	68 [10–178] $\uparrow$ (*p* < 0.001)	52 [7–112] $\downarrow$ (*p* = 0.006)	54 [7–118] (*p* = 0.310) $\downarrow$ (*p* < 0.001)^a^ (*p* = 0.070)^b^

	TRC scenarios			
	TRCall (reference)	TRCcore	TRCfemale	TRCcorefemale
Number (over 10 years)				
Captured	264 [224–301]	252 [196–294] $\downarrow$ (*p* < 0.001)	144 [105–181] $\downarrow$ (*p* < 0.001)	124 [66–160] $\downarrow$ (*p* < 0.001) $\downarrow$ (*p* < 0.001)^a^ $\downarrow$ (*p* < 0.001)^b^
Removed	48 [7–126]	58 [8–142] $\uparrow$ (*p* < 0.001)	39 [6–89] $\downarrow$ (*p* < 0.001)	34 [6–81] $\downarrow$ (*p* < 0.001) $\downarrow$ (*p* < 0.001)^a^ $\downarrow$ (*p* = 0.001)^b^
Culled	131 [114–148]	131 [113–147] (*p* = 0.938)	107 [86–123] $\downarrow$ (*p* < 0.001)	99 [77–121] $\downarrow$ (*p* < 0.001) $\downarrow$ (*p* < 0.001)^a^ $\downarrow$ (*p* < 0.001)^b^

Results obtained under the short-delay in density-dependence assumption (see Additional file 3 for the medium-delay and the long-delay in density-dependence assumptions). Results are indicated as median [95% credible intervals]. The p-values of the Mann–Whitney test for the distributions of the outputs compared to TR(C)all (reference) are indicated in parentheses. All scenarios had an objective of 50 individuals captured per year, and TRC scenarios had an additional objective of 20 unmarked individuals culled per year.

TR: test-and-remove, TRC: TR combined with the culling of unmarked individuals, TR(C)core: targeted towards the core area of the massif, TR(C)female: targeted towards females, TR(C)corefemale: targeted towards both females and the core area (multilevel).

^a^Reference: TR(C)core.

^b^Reference: TR(C)female.

### Targeted test-and-remove

When targeting the core area (TRcore), the total number of captures after 10 years slightly decreased compared to the untargeted strategy (TRall), but more seropositive individuals were euthanized and removed (Figure 2, Table 3 and Additional file 3), resulting in a significantly lower seroprevalence at the end of the simulations (Figure 2 and Additional file 3). The proportions of simulations where brucellosis faded out were higher for TRcore compared to TRall, although not significantly (Figure 2 and Additional file 3).

When targeting only females in the whole massif (TRfemale) or in the core area (TRcorefemale), the total number of captures dropped (Table 3 and Additional file 3), which reflects the fact that there were less females available than the numbers that were targeted. The number of individuals removed also decreased compared to the untargeted strategy (Figure 2, Table 3 and Additional file 3). Nonetheless, the seroprevalence at the end of the simulations was significantly lower for TRfemale and TRcorefemale compared to TRall, except under the long-delay assumption (Figure 2 and Additional file 3).

Although the number of individuals removed was the smallest in the TRcorefemale scenario, the seroprevalence was similar (medium- and long-delay assumptions) or significantly lower (short-delay assumption) in TRcorefemale compared to TRfemale (Figure 2 and Additional file 3).

The results were more contrasted, with significantly lower seroprevalence (short-delay assumption), non-significant difference (medium-delay assumption) or higher seroprevalence (long-delay assumption), when comparing TRcorefemale with TRcore (Additional file 3). Moreover, the probability of *Brucella* extinction was always lower for TRcorefemale compared to TRcore (Additional file 3).

### Targeted test-and-remove combined with culling

When targeting the core area (TRCcore), the total number of captures after 10 years slightly decreased compared to the untargeted strategy (TRCall), but similar (medium- and long-delay assumptions) or higher (short-delay assumption) number of individuals were removed (Table 3 and Additional file 3). The total number of individuals culled after 10 years slightly decreased (medium- and long-delay assumptions) or remained stable (short-delay assumption) compared to the untargeted strategy (Table 3 and Additional file 3). Overall, the total number of individuals removed and culled decreased for TRCcore compared to TRCall, except for the short-delay assumption (Figure 2 and Additional file 3). Nonetheless, the seroprevalence at the end of the simulations was always significantly lower for TRCcore compared to TRCall (Figure 2 and Additional file 3). The proportions of simulations where brucellosis faded out were higher for TRCcore compared to TRCall, although not always significantly (Figure 2 and Additional file 3).

When targeting only females in the whole massif (TRCfemale) or in the core area (TRCcorefemale), the total number of captures dropped (Table 3 and Additional file 3), which reflects the fact that there were less females available than the numbers that were targeted. The number of individuals removed and culled also decreased compared to the untargeted strategy (Figure 2, Table 3 and Additional file 3). Nonetheless, the seroprevalence at the end of the simulations was significantly lower for TRCfemale and TRCcorefemale compared to TRCall, except under the long-delay assumption (Figure 2 and Additional file 3). However, the probability of *Brucella* extinction decreased for TRCfemale and TRCcorefemale compared to TRCall, except under the short-delay assumption (Figure 2 and Additional file 3).

Although the number of individuals removed and culled was the smallest in the TRCcorefemale scenario, the seroprevalence was similar (long-delay assumption) or significantly lower (short- and medium-delay assumptions) in TRCcorefemale compared to TRCfemale (Figure 2 and Additional file 3).

The results were more contrasted, with significantly lower seroprevalence (short-delay assumption), non-significant difference (medium-delay assumption) or higher seroprevalence (long-delay assumption), when comparing TRCcorefemale with TRCcore (Additional file 3). Moreover, the probability of B*rucella* extinction was always lower for TRCcorefemale compared to TRCcore (Additional file 3).

## Discussion

Using brucellosis in Alpine ibex from the Bargy massif as a case study, we simulated feasible disease management strategies, including ones targeting individuals and/or areas having a particular role in transmission, in mitigating brucellosis seroprevalence and persistence after 10 years of model simulations. Beside measuring several indicators of the sanitary benefits of these scenarios, we estimated the numbers of individuals captured, removed (euthanized at capture) and culled, in order to provide information useful to approach the costs of interventions. These numbers are not direct estimates of operational costs, since for example capturing needs to get closer to the targeted individual and is thus more difficult than culling. However, these numbers are a prerequisite to estimate the cost of each strategy, in terms of operational and financial costs, but also in terms of population conservation and acceptability of measures.

### Brucellosis persistence

Brucellosis faded-out only in a small proportion of the simulations during the study period of 10 years. As for any infection in finite populations, extinction of the infectious agent is inevitable [41], but, given the results of our model, spontaneous extinction (especially for the do nothing strategy) should not be expected in a near future. This would be even more the case if the demographic parameters should happen to increase in the next few years as a result of delayed density-dependent responses expected after the massive culling and test-and-remove operations conducted in 2013 and 2015, which led to a drastic decrease in population density. Indeed, no increase in female reproductive performance was detected to date, several years after the drop in population size induced by these operations, but such an increase could occur after a delay of several years linked to cohort effects. Indeed, in ungulates, the conditions experienced during the year of birth have long-lasting effects on female individual performance, and as long as females born at high density represent a significant part of reproductive females, an increase in performance is barely expected to be detected at the population level (see e.g. [42, 43]). Our results showed that the sooner the demographic parameters increased, the longer brucellosis persisted and the higher the seroprevalence was in the population after 10 years. This result is in accordance with the theoretical model of Lloyd-Smith et al. [41], where faster demographic turnover favours longer persistence, because a higher number of susceptible individuals are born each year. Therefore, the monitoring of demographic parameters, such as population size estimates and female reproductive performance, will be essential to detect any increase early and to adapt management strategies.

### Efficacy of management interventions

Our model did not include any estimate of economical, ethical or societal (acceptability) costs and benefits, as these were beyond the scope of this paper. However, these considerations obviously have to be considered to elaborate management scenarios. Our results provide estimates of the impact on population size, and number of individuals captured and culled, in order to provide figures that could be integrated in such analyses.

Test-and-remove decreased brucellosis persistence and seroprevalence in the population when the objective was to capture 50 individuals each year (Figure 2 and Additional file 3). Higher levels of captures could increase the probability of capturing and removing infectious individuals, and, as a result, have a higher impact on transmission. However, the level of captures of 50 individuals each year was chosen because it was considered as feasible in practice given operational costs and field conditions. In particular, the population size decreased since the first management interventions, while the proportion of marked individuals increased [35]. As a result, there are less individuals, and especially unmarked individuals, available for captures. Moreover, the flight initiation distance increased, making captures more and more difficult every year (unpublished data).

The addition of culling 20 unmarked individuals compared to TR alone was slightly more effective in decreasing seroprevalence and brucellosis persistence, especially for medium- and long-delay assumptions for the delay in the response of density-dependent parameters (Figure 2 and Additional file 3). In the TRC scenarios, there was a competition between test-and-remove and culling for the access to unmarked individuals, leading to less captures and less seropositive individuals removed than in TR scenarios (Tables 3, 4 and Additional file 3). This decrease in number of seropositive individuals removed thus had to be compensated by the effect of culling. This effect was probably lower under the short-delay assumption, because the number of individuals culled represented a lower proportion of the overall population as the growth rate of the population rapidly increased. These increased sanitary benefits obtained for TRC were however associated with significant costs in terms of population conservation, as the total number of individuals removed and culled was significantly higher and the population size significantly lower than in TR alone (Figure 2, Tables 3, 4 and Additional file 3). The cost–benefit balance of TR compared to TRC strategies will therefore have to be taken into account by managers and decision-makers for the choice of future strategies implemented in the Bargy massif population.

**Table 4:** **Summary of differences between test-and-remove alone or combined with culling.**

Scenario	TRC vs TR											
	All			Core			Female			Corefemale		
${d}_{dens}$	0	5	10	0	5	10	0	5	10	0	5	10
Seroprevalence	NS	$\downarrow$	$\downarrow$		$\downarrow$		$\downarrow$	$\downarrow$	NS		$\downarrow$	
Probability of *Brucella* extinction	NS	$\uparrow$	$\uparrow$	NS	NS	$\uparrow$	NS	NS	$\uparrow$	NS	NS	$\uparrow$
Population size		$\downarrow$			$\downarrow$			$\downarrow$			$\downarrow$	
Number (over 10 years)												
Captured		$\downarrow$			$\downarrow$			$\downarrow$			$\downarrow$	
Removed		$\downarrow$			$\downarrow$			$\downarrow$			$\downarrow$	
Removed + culled		$\uparrow$			$\uparrow$			$\uparrow$			$\uparrow$	

Arrows indicate significant (*p* < 0.05) increase ($\uparrow$) or decrease ($\downarrow$) of model outputs as indicated by the chi-squared (probability of *Brucella* extinction) or Mann–Whitney (all other outputs) tests comparing the results between TRC scenarios and their TR counterparts. “NS” indicates the absence of significant differences. Output values are detailed in Figure 2, Table 3 and Additional file 3. The delay in density-dependent responses, ${d}_{dens}$, takes different values according to the assumption (“short-delay” assumption: ${d}_{dens}=0$ years; “medium-delay” assumption: ${d}_{dens}=5$ years; “long-delay” assumption: ${d}_{dens}=10$ years).

TR: test-and-remove, TRC: TR combined with the culling of unmarked individuals, TR(C)core: targeted towards the core area of the massif, TR(C)female: targeted towards females, TR(C)corefemale: targeted towards both females and the core area (multilevel).

### Targeting specific areas

Strategies that targeted the core area of the massif, where seroprevalence is higher and most transmissions happened [31, 33], were as or more effective than their counterparts that were applied to the whole massif (Figure 2 and Additional file 3). For similar levels of captures, more seropositive individuals would be removed in test-and-remove operations when the core area was specifically targeted (Table 3 and Additional file 3), which explains at least in part why it was more effective. However, test-and-remove combined with culling targeted towards the core area of the massif was still associated with increased sanitary benefits, although this time less individuals were removed and culled. These results confirm the prediction that removing individuals from the core area is more effective than removing individuals in the whole massif, because of the predominant role these individuals play in transmission.

### Targeting gender

In most scenarios, targeting females decreased the seroprevalence compared to untargeted interventions (Figure 2 and Additional file 3), despite the fact that the number of seropositive individuals removed was lower (Table 3 and Additional file 3). These results confirm the prediction that removing females is more effective than removing both genders, because females are responsible for the vast majority of transmission in the system [33].

However, it should be noted that the proportions of simulations where *Brucella* faded out were sometimes significantly lower when only females were targeted compared to their counterparts targeting both sexes (Figure 2 and Additional file 3). Although transmission of brucellosis was clearly decreased when targeting only females, males were never the target of any management intervention. Therefore, although they transmit infection at a lesser extent than females, males could stay infected during management interventions, and thereby increase brucellosis persistence after 10 years of model simulations. One other possible limitation to this refinement is the amount of effort and time required and associated costs, which are partially reflected by the smaller number of captures, removals and culls achieved when targeting only females (Table 3 and Additional file 3). Because of this limitation, the number of individuals removed and culled could become too low and impede the increased efficacy obtained by targeting females.

Although these limitations should be taken into account by managers and decision-makers, it should also be noted that field agents are more flexible than our model. For example, if females are not available during one capture intervention in a specific area, agents could target males from the same area instead, which was not permitted in our model. Therefore, such a flexible strategy could result in higher numbers of captures and removals, and therefore in even higher efficacy, than the results we provided here.

In addition to a better efficacy in decreasing brucellosis seroprevalence and transmission, strategies targeting females and/or the core area also had less impact in terms of population conservation and acceptability, as revealed by the often lower numbers of ibex removed and culled compared to their counterparts targeting both sexes (Table 3 and Additional file 3). This is an important component for the choice of an optimal management strategy, as it could represent a more acceptable alternative than population-wide strategies by decreasing the costs-benefits balance.

### Surveillance and management perspectives

The objective of brucellosis management is to maintain France’s officially free status, and therefore to mitigate the risk of brucellosis transmission to livestock. The aim is therefore to control infection in the ibex population to reduce the risk of interspecies transmission, and ideally to increase the likelihood of fade-out by reducing infection. As demonstrated with our model, management interventions under consideration in the study population are likely to have a significant impact on both seroprevalence and probability of *Brucella* extinction in the population. However, all interventions are likely to have a significant impact on the ibex population as long as management interventions are in place. This impact should be minimized whenever possible, bearing in mind that if *B. melitensis* extinction is achieved, management interventions will be interrupted and the population will be able to recover up to its carrying capacity.

One important result of our study is the sensitivity of the epidemiological dynamics and the efficacy of management strategies to the delay in density-dependent responses of population parameters. Therefore, the monitoring of the reproductive success and the population size is critical, and should be continued to detect early changes in population dynamics.

Epidemiological surveillance should also be maintained alongside management interventions, to monitor the efficacy of any management strategies implemented in the field. In particular, unexpected ecological or behavioural feedbacks could lead to counterintuitive results of management interventions [9, 11]. Although in the study population no changes of space use or social organization was observed following the 2013 massive culling operation (C. Calenge, pers. comm.; [31]), behavioural disruption following future management interventions cannot be excluded. Although these counterintuitive effects were not explored with our model, targeted interventions in particular could lead to behavioural changes. For instance, reducing the number of females or depleting a particular subpopulation could lead to increase movements or dispersal between subpopulations, which could be hard to predict.

Overall, any strategy should be implemented in an adaptive management framework, where new data collected alongside ongoing interventions should be used to inform and adapt future strategies. Our model could be used in the future in this framework, by using new data to improve model fit and then re-evaluate possible scenarios.

### Perspectives on further research

Beside management-related parameters and the delay in density-dependent responses, the sensitivity analysis pointed out three key parameters having significant influence in determining the variability of model outputs: the carrying capacity, the per-capita probability of one individual coming into effective contact with one infectious abortion or birth per week, and the probability of successful venereal transmission given contact between an infectious and a susceptible host. These three parameters were already estimated by fitting the model to available data in a previous study [33], but our results demonstrate that using additional data to parameterize the model would help reduce uncertainty.

In particular, the per-capita probability of one individual coming into effective contact with one infectious abortion or birth per week plays a critical role on the seroprevalence and the probability of *Brucella melitensis* extinction. The current assumption is that horizontal transmission by infectious abortions or births is density-dependent within a given socio-spatial unit, i.e., depends on the number of infectious events within the unit. However, variation in population size within unit may lead to more complex patterns of transmission dynamics [41]. Further research would be needed to better characterise the frequency of contacts with those infectious events and its variation.

Further management scenarios could be evaluated by our model in the future. For instance, in bison in the Greater Yellowstone Area, a selective test-and-remove focusing on pre-reproductive females was more effective compared to the same strategy in females regardless of age [20]. The rationale behind this result was that those females were removed before they could shed *Brucella* during their first pregnancies, and was more effective than removing females that already shed the bacteria in previous pregnancies but already recovered from the infection [20]. Targeting younger females either for capture or for culling could therefore represent an even more relevant option, by increasing the sanitary benefits while decreasing the costs in terms of population conservation compared to strategies targeting females regardless of age.

Our results confirmed our predictions that targeting specific classes of individuals or specific areas that play a major role in transmission is more effective than untargeted management, and also that multilevel strategies targeting both are even more effective, which is an original contribution of our study. For brucellosis in the ibex population of the Bargy massif, this is achieved by targeting the central units of the population, that are transmission hotspots, and females, that play a predominant role in transmission compared to males. However, there is no silver bullet for the management of brucellosis in this population as all management strategies that we evaluated had limitations, with brucellosis fade-out not predicted to happen in a near future. Combination of test-and-remove with culling appeared to be better than test-and-remove alone. Targeted management represents a valuable option for managing wildlife infectious diseases, and offers a wide range of possible refinements to classical sanitary measures. We therefore encourage to look for heterogeneity in other systems and to evaluate potential strategies for improving management in terms of efficacy but also in terms of operational costs, population conservation costs and acceptability.

## Supplementary Information

**Additional file 1. Model description.** [10, 31–33, 36, 37, 44–70] This file contains the full description of the individual-based model following the updated “Overview”, “Design concepts”, “Detail” protocol, and the complete system of mathematical equations.

**Additional file 2. Sensitivity analysis.** [33, 36, 38, 39, 44–47, 51, 52, 55, 60, 66, 71–73] This file contains the framework and detailed results of the sensitivity analysis.

**Additional file 3. Comparison of management scenarios under varying assumptions for the delay in density-dependent responses.** This file contains additional figures and tables showing model outputs for different assumptions.

**Additional file 4. Test-and-vaccinate-or-remove strategy.** [30, 35, 36, 40, 74–80] This file describes the methods, the results and the conclusions for an additional management scenario.

## Data Availability

The model was parameterized in a previous study, using data available in the Appendix of the publication (available at: https://doi.org/10.1016/j.ecolmodel.2020.109009).

## Abbreviations

S: susceptible
E: exposed
I: infectious
R: recovered
NO: do nothing
TR: test-and-remove
TVR: test-and-vaccinate-or-remove
TRC: test-and-remove combined with culling
MWtests: Mann–Whitney tests
χ^2^ tests: Chi-squared tests
