# Supplementary material for: Targeted strategies for the management of wildlife diseases: the case of brucellosis in Alpine ibex
Source: Vet Res. 2021 Sep 14;52:116. doi: 10.1186/s13567-021-00984-0 (PMC8439036; doi:10.1186/s13567-021-00984-0)
Supplement: Supplementary file 1 — Additional file 1. Model description. [10, 31–33, 36, 37, 44–70] This file contains the full description of the individual-based model following the updated “Overview”, “Design concepts”, “Detail” protocol, and the complete system of mathematical equations. [file 13567_2021_984_MOESM1_ESM.docx]

**ADDITIONAL FILE 1: Model description**

We describe the individual-based model we built following the updated ODD protocol (“Overview”, “Design concepts”, “Details”) of Grimm et al. [48, 49].

**1. Overview**

**1.1. Purpose**

We expanded an individual-based, spatially implicit SEIR (Susceptible, Exposed, Infectious, Recovered) model developed in a previous study [33]. In the present study, the purpose of the model was to evaluate the relative efficacy of various management strategies, taking into account the strong spatial structure of the studied population [31] and the heterogeneity of brucellosis transmission highlighted in the previous modelling study [33] arising from differences among individuals in terms of social behaviour, space use, mating behaviour, transmission routes or active infection. We used an individual-based model for this purpose because it offers the possibility to take into account numerous individual specificities that can impact the infection dynamics [50] and to target individuals for management based on these specificities.

**1.2. Entities, state variables, and scales**

The only entities in the model were ibex individuals. To track information on each individual at each time step, the model included several state variables: individual ibex identity, sex, age in weeks, socio-spatial unit, health status (susceptible: $S$; exposed: $E$; actively infected: $I;$ or non-actively infected, i.e., non-shedder: $R$), year of infection, year of recovery and reproductive status (sexually receptive, pregnant, abortion, birth, parity).

The population is structured into five socio-spatial units [31]. Within each socio-spatial unit, ibex have the tendency to form groups, but they experience very loose bonds: groups are not stable and split and merge frequently (fission-fusion dynamics – C. Calenge, pers. comm.). To account for this structure in the model, we used a spatial metapopulation model, often applied to populations naturally subdivided into spatial units, with homogeneous contacts within subpopulations [50]. At birth, each individual was attributed to the socio-spatial unit of its mother (permanent socio-spatial unit). All individuals living in the same socio-spatial unit constituted a subpopulation, each subpopulation being characterised by its name and its own relative carrying capacity, defined as a proportion of the carrying capacity of the whole population. The five subpopulations constituted the overall metapopulation.

The model was developed to represent the evolution of brucellosis transmission and population dynamics for ten years starting on December 1^st^, 2018, with a discrete weekly time step. This time step was chosen because some processes are expected to occur at the week level (e.g., incubation period of 3 weeks) and a weekly time step remains feasible by our simulation process.

**1.3. Process overview and scheduling**

Simulated years began on December 1^st^, and were split into two periods: the mating period, which lasts from early December to mid-January in ibex [51, 52] and was therefore fixed at 7 weeks in the model; and the non-mating period for the remaining 45 weeks. During the mating period $\left( 1\leq t\leq d_{rut} \right)$, the events occurring successively at each discrete time step were mortality, spatial movements, reproduction, venereal transmission, incubation and recovery. During the non-mating period $\left( d_{rut}+1\leq t\leq52 \right)$, the events occurring successively at each discrete time step were management strategies, mortality, spatial movements, abortions or births, congenital/pseudo-vertical transmission, horizontal transmission, incubation and recovery. Such an order is artificial but is required to apply probabilities of dying or getting infected to the correct numbers of individuals. For each event, individuals were processed simultaneously.

Alpine ibex show a strong seasonal pattern of reproduction, and thus of brucellosis transmission. The mating period is short, from early December to mid-January, and *ca.* 70% of mating associations between males and females occur during the first 2 weeks [52], which produces a seasonal peak of births of susceptible individuals in late May and early June. Social structure is also subject to seasonal variation, because of progressive sexual segregation [53] with consequences on relevant contact patterns for transmission. These seasonal patterns were important to take into account because of their expected impacts on disease dynamics.

Gestation in ibex lasts 167 days [54], which leads births to take place from the end of May to mid-July, i.e., 24 weeks after the mating period. Abortions caused by brucellosis occur during the last third of gestation [37], i.e., 16 weeks after the beginning of gestation for an ibex. In domestic ruminants, shedding of *Brucella* through placenta, foetal fluids and vaginal discharges when a female aborts or gives birth may last *ca.* three weeks [55], and we assumed a similar duration for ibex in the model. Therefore, horizontal transmission of *Brucella* through abortions is possible from early April to the end of May. However, after parturition, an ibex female tends to isolate itself in cliffs and rocky areas with its newborn for two weeks [53]. Thus, other individuals were assumed to be in contact with a female only the third week after its parturition, reducing the period of exposure to this transmission route.

**2. Design concepts**

The model considers ibex demography, transmission of infection within- and between-unit, and management strategies. Population dynamics emerge from individual reproduction and mortality, which parameters depend on age, sex and density.

Infection dynamics emerge from contacts between susceptible and actively infected individuals and from probabilities of successful transmission given those contacts for each transmission routes. For venereal transmission, contacts emerge from male mating tactic and from female sexual receptivity, described by empirical rules and probabilities. Thus, venereal transmission depended only on the mating system of the host and not on the population size, and can thus be labelled as frequency-dependent [56]. For horizontal transmission of *Brucella* through infectious abortions or births, contacts were considered as homogeneous inside a socio-spatial unit, i.e., all individuals could contact each other. Thus, this horizontal transmission increases with the population size (i.e., density-dependent [56]). For transmission between subpopulations, we used a mechanistic model that explicitly represented movements of individuals, representing opportunities of contacts and transmission between individuals from different subpopulations [57]. Heterogeneity of transmission can emerge from age- and sex-specific variation in these contact and transmission patterns.

For the model analysis, the individual statuses were used to derive population-level variables such as population size or seroprevalence.

Transitions between states were modelled as stochastic flows assuming demographic stochasticity. Each transition was the outcome of a Bernoulli process.

**3. Details**

**3.1. Initialisation**

The initial population for the first time step of the model was based on the simulated population at the last time step of the previously published model [33]. This previous model ran for six years (between December 1, 2012 and November 30, 2018) and was calibrated by fitting three parameters to observed data using Approximate Bayesian Computation (ABC) rejection algorithm (see [33] for details). Simulations with 1000 iterations, each iteration using a set of parameter values from the 1000 sets retained in the ABC, produced predictions in accordance with observations both qualitatively and quantitatively [33]. Therefore, we used the 1000 simulated populations at the last time step as initial conditions for our current study.

**3.2. Input**

The model does not use input data or an external model to represent time series of driving environmental variables.

**3.3. Submodels**

The equations describing model processes are given below (1-22). See Table A1 for the parameter values.

***3.3.1. Density-dependence***

*Implementation:*

Each year, the probability of winter juvenile mortality $\mu_{0}$, as well as the probability for a female to become sexually receptive $\tau$, had density-dependent responses at the socio-spatial unit level that were shaped to reproduce a logistic population growth [58].

**Table A1: Definition of model parameters to evaluate management of brucellosis in ibex (Bargy massif, France).**

| **Submodel** | Symbol | Description (dimension) | Value | References |
| --- | --- | --- | --- | --- |
| **Density-dependence** | $d_{dens}$ | Delay between the beginning of the simulations and the relaxation of the density-dependent regulation (years) | 0; 5; 10 | * |
|  | $k$ | Threshold for density-dependent effect (individuals) | 131 | * |
|  | $K$ | Carrying capacity of the metapopulation (individuals) |  | † |
| **Management** | $d_{man}$ | Duration of management interventions (years) | 10 | ‡ |
|  | $n_{capt}$ | Objective level for the total number of individuals to be captured (per year) | 50 | ‡ |
|  | $n_{cull}$ | Objective level for the total number of individuals to be culled (per year) | 20 | ‡ |
|  | $Se$ | Sensitivity of serologic tests | 0.95 | [36] |
| **Mortality** | $\mu_{0}^{min}$ | Probability of winter mortality of newborns, minimum (over the duration of winter) | 0 | * |
|  | $\mu_{0}^{max}$ | Probability of winter mortality of newborns, maximum (over the duration of winter) | 0.60 | * |
|  | $\mu_{X♀}$ | Probability of mortality of X-years old females (annual) | Table A3 | § |
|  | $\mu_{X♂}$ | Probability of mortality of X-years old males (annual) | Table A3 | § |
| **Movements** | $p_{ij}^{'}$ | Probability for a male whose permanent unit is $i$to visit unit $j$ during one time step (per week) | Figure A3 | § |
| **Reproduction** | $\tau_{X}^{max}$ | Sexual receptivity probability of X-years old females, maximum | Table A2 | * |
|  | $\tau_{X}^{min}$ | Sexual receptivity probability of X-years old females, minimum | Table A2 | * |
|  | $\zeta_{\tau}$ | Impact of brucellosis on sexual receptivity probability of infectious females | 0.80 | * |
|  | $d_{rut}$ | Duration of mating period (weeks) | 7 | [52] |
|  | $p_{1}$ | Proportion of associations during the first 2 weeks of the mating period | 0.70 | [52] |
|  | $p_{2}$ | Proportion of associations during the rest of the mating period | 0.30 | [52] |
|  | $n_{T}$ | Number of tending males per female (over the duration of mating) | 1 | [52] |
|  | $n_{C}$ | Number of coursing males per female (over the duration of mating) | 3 | [52] |
|  | $p_{C1}$ | Proportion of coursing males 2-5 years old associated to females | 1/3 | [59] |
|  | $p_{C2}$ | Proportion of coursing males 6-7 years old associated to females | 2/3 | [59] |
|  | $\tau_{T}$ | Copulation probability of tending males (8-14 years old) | 6/7 | [59] |
|  | $\tau_{C}$ | Copulation probability of coursing males (2-7 years old) | 1/7 | [59] |
|  | $n^{max}$ | Maximal number of partners for males (per week) | 3 | [52] |

* Experts knowledge

† Accepted parameter values in the Approximate Bayesian Computation [33]

‡ Tailored to represent realistic management interventions in the study population

§ Calibrated using field data

**Table A1 (continued).**

| **Submodel** | Symbol | Description (dimension) | Value | References |
| --- | --- | --- | --- | --- |
| **Venereal transmission** | $\nu_{ven}$ | Probability of successful venereal transmission from tending males to females given contact |  | † |
|  | $\kappa$ | Relative efficacy of venereal transmission from coursing males | 0.45 | [52] |
|  | $\omega$ | Relative efficacy of female-to-male venereal transmission | 0.60 | [60] |
| **Abortions or births** | $d_{abo}$ | Delay between mating and abortion due to *Brucella* (weeks) | 16 | [37] |
|  | $\rho$ | Probability of abortion during the first pregnancy following infection | 0.80 | [61] |
|  | $d_{gest}$ | Duration of gestation (weeks) | 24 | [54] |
|  | $\eta_{1}$ | Probability of giving birth for primiparous females | 0.70 | * |
|  | $\eta_{2}$ | Probability of giving birth for multiparous females | 0.90 | * |
|  | $\zeta_{\eta}$ | Impact of brucellosis on probability of giving birth for infectious females in subsequent pregnancies | 0.90 | * |
|  | $\delta$ | Sex ratio | 0.50 | * |
| **Vertical transmission** | $d_{isol}$ | Duration of postpartum isolation of the mother-offspring couple (weeks) | 2 | [51] |
|  | $\beta_{cong}$ | Congenital transmission probability by *in utero* infection | 0.05 | [47] |
|  | $\beta_{pseu}$ | Pseudo-vertical transmission probability by milk ingestion | 0.05 | * |
| **Horizontal transmission** | $d_{shed}$ | Duration of shedding of *Brucella* in genital fluids when a female aborts or gives birth (weeks) | 3 | [55] |
|  | $\beta_{IA}$ | Per capita probability of one female coming into effective contact with one infectious abortion due to *Brucella* (per week) |  | † |
|  | $\sigma$ | Reduction in the number of contacts with infectious abortion for males ≤5 years old | 0.27 | § |
|  | $\phi$ | Reduction in the number of contacts with infectious abortion for males >5 years old | 0.06 | § |
|  | $\beta_{IB}$ | Per capita probability of one female or one newborn coming into effective contact with one infectious birth (per week) |  | † |
| **Incubation and recovery** | $d_{inc}$ | Duration of *Brucella* incubation (weeks) | 3 | [55] |
|  | $\gamma$ | Probability of recovery (annual) | 0.16 | § |

* Experts knowledge

† Accepted parameter values in the Approximate Bayesian Computation [33]

‡ Tailored to represent realistic management interventions in the study population

§ Calibrated using field data

For these parameters, we used sigmoid functions (1-3) using explicit variables ($k(u)$*:* the threshold for density-dependent effect in unit $u$, $K(u)$: the carrying capacity of unit $u$, $N(y^{*},u)$: the annual pre-breeding population size in unit $u$). $N(y^{*},u)$ was the simulated population size of 2013 (before the massive culling operation conducted in the population) in unit $u$ if $y\leq d_{dens}$, and the simulated population size at the beginning of year $y$ otherwise. See Table A1 and Table A2 for the parameter values, and Figure A1 for the graphical shape of the functions of equations 1-3 [33, 36].

Each unit was characterized by its own relative carrying capacity, defined as a proportion of the carrying capacity of the whole population. Between 2012 and June 2018, 508 different recorded individuals were old enough to be already present in the population in 2012. We considered that this sample was large enough to be representative of the population (estimated carrying capacity: 564 [535–591]), and therefore we extrapolated the repartition of these 508 different individuals among socio-spatial units to the total population in our model. These proportions were: 7.5% for Unit 1, 8.1% for Unit 2, 61.3% for Unit 3, 10.1% for Unit 4 and 13.0% for Unit 5 [33].

| (1) | $\mu_{0}\left( y,u \right)=\left\{ \begin{aligned} \mu_{0}^{min} , &\mathrm{when}N(y^{*},u)<k(u) \\ 1-\frac{1}{e^{-a}+1}, &\mathrm{when} k(u)<N(y^{*},u)<K(u) \\ \mu_{0}^{max} , &\mathrm{when}N\left( y^{*},u \right)>K(u) \end{aligned} \right.$ |
| --- | --- |
|  | $\begin{matrix} \mathrm{Where} & a=\left( \frac{N(y^{*},u)-k(u)}{K(u)-k(u)} \right)logit\left( 1-\mu_{0}^{max} \right)+\left( \frac{K(u)-N(y^{*},u)}{K(u)-k(u)} \right)logit\left( 1-\mu_{0}^{min} \right) \end{matrix}$ |
| (2) | $\tau_{X}\left( y,u \right)=\left\{ \begin{aligned} \tau_{X}^{max} , &\mathrm{when}N(y^{*},u)<k(u) \\ \frac{1}{e^{-a}+1}, &\mathrm{when} k(u)<N(y^{*},u)<K(u) \\ \tau_{X}^{min} , &\mathrm{when}N\left( y^{*},u \right)>K(u) \end{aligned} \right.$ |
|  | $\begin{matrix} \mathrm{Where} & a=\left( \frac{N(y^{*},u)-k(u)}{K(u)-k(u)} \right)logit\left( \tau_{X}^{min} \right)+\left( \frac{K(u)-N(y^{*},u)}{K(u)-k(u)} \right)logit\left( \tau_{X}^{max} \right) \end{matrix}$ |
|  | and $X$ corresponds to age $1-11$. |
| (3) | $\tau_{12-16}\left( y,u \right)=\left\{ \begin{aligned} \frac{1}{e^{-a}+1}, &\mathrm{when} N(y^{*},u)<K(u) \\ \tau_{12-16}^{min} , &\mathrm{when}N\left( y^{*},u \right)>K(u) \end{aligned} \right.$ |
|  | $\begin{matrix} \mathrm{Where} & a=\left( \frac{N(y^{*},u)-k(u)}{K(u)-k(u)} \right)logit\left( \tau_{12-16}^{min} \right)+\left( \frac{K(u)-N(y^{*},u)}{K(u)-k(u)} \right)logit\left( \tau_{12-16}^{max} \right) \end{matrix}$ |

**Table A2: Values of sexual receptivity probability of females depending on age.**

|  | $X$ (age in years) | | | | | |
| --- | --- | --- | --- | --- | --- | --- |
|  | 0 | 1 | 2-3 | 4-11 | 12-16 | 17-19 |
| $\tau_{X}^{max}$ | - | 0.50 | 0.80 | 1 | 0.80 | - |
| $\tau_{X}^{min}$ | - | 0 | 0 | 0.50 | 0.30 | - |


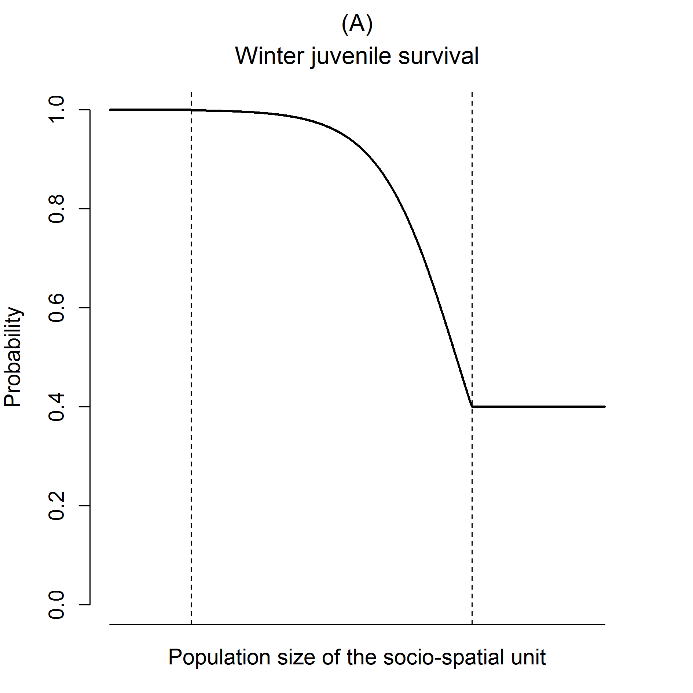

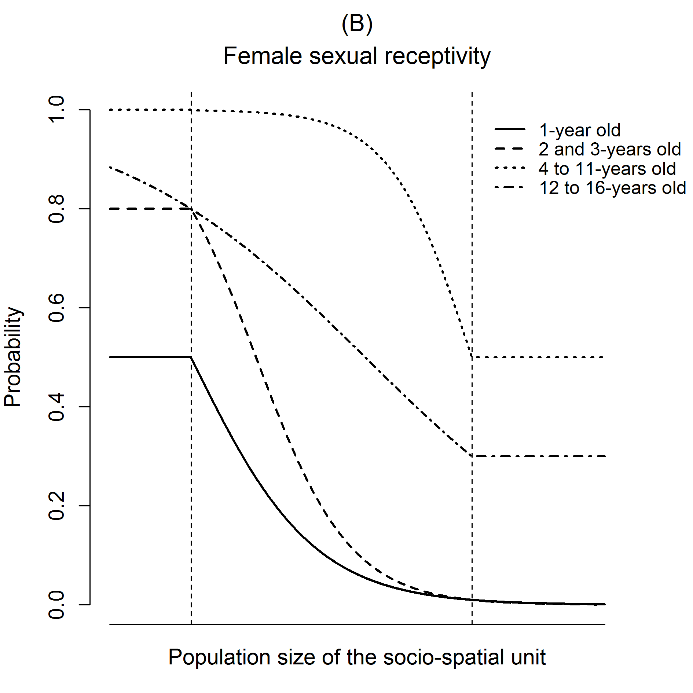


**Figure A1: Winter juvenile survival (A) and female sexual receptivity (B) function of the unit’s population size.**

Vertical dashed lines represent the values of $k(u)$*:* the threshold for density-dependent effect in unit $u$ and $K(u)$: the carrying capacity of unit $u$.

*Rationale:*

Density-dependence of survival and reproduction is a common process in populations of large herbivores [62]. Density-dependent responses of population parameters were shaped to reproduce a logistic population growth, where the population size stabilises around the carrying capacity in the absence of management interventions. Indeed, previous studies of population dynamics of ibex have shown that the logistic model may be appropriate to describe the density regulation in ibex populations [58]. Density-dependent processes play a crucial role in disease ecology because it can interact with transmission, for example by increasing the number of births and therefore the pool of susceptible individuals after population size reduction [10]. In the case of brucellosis, density-dependence of reproduction is also critical because all transmission routes are related to reproduction processes.

The massive culling operations of 2013 and 2015 did not induce any increase in female reproductive success to date, despite the drop in population size (C. Toïgo, pers. comm.). This may be explained by a delay on density-dependent responses, that need several years before they can occur [36]. Although no increase in reproduction parameter was observed to date, the duration of this delay remains uncertain.

| **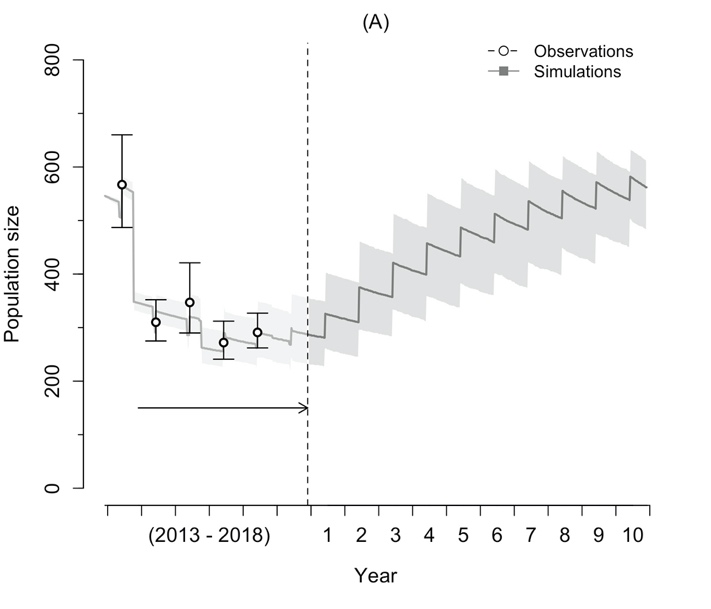** | **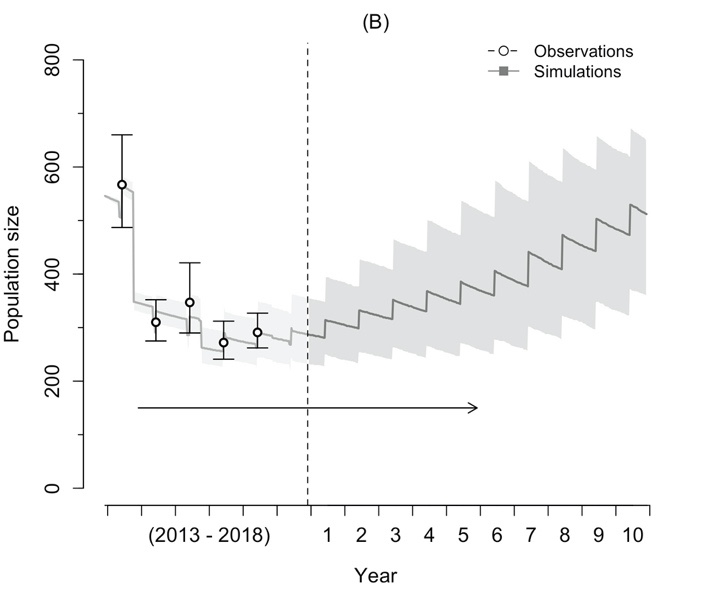** | **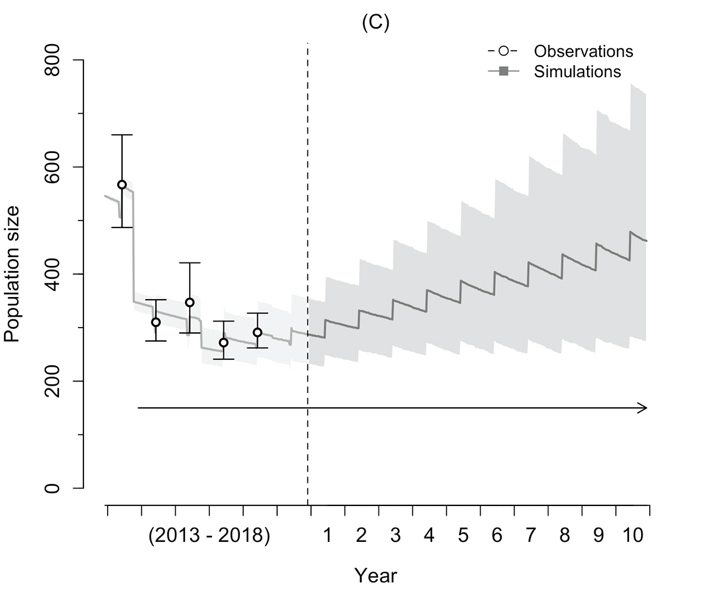** |
| --- | --- | --- |

**Figure A2: Evolution of the population size without newborns (<1 year old) over time.**

(A) Short-delay assumption: the density-dependent regulation of population parameters relaxed the first year of the simulated management scenario. (B) Medium-delay assumption: the density-dependent regulation of population parameters relaxed after five years of the simulated management scenario. (C) Long-delay assumption: the density-dependent regulation of population parameters did not relax over the ten years of the simulated management scenario. The open black circles and black lines are the observed data and the 95% confidence interval, respectively. The grey line represents the median of the simulated output over the 1000 iterations, while the grey area represents the 95% credible interval. The vertical dashed line represents the separation between the 2013-2018 period, fitted to the observed dynamics of the population (previous modelling study; [33]), and the ten consecutive years of simulated “Do Nothing” management scenario (current study).

We considered three possible assumptions to account for this uncertainty (Figure A2): (*i*) the “short-delay” assumption, where the density-dependent regulation of population parameters relaxed the first year of the simulations ($d_{dens}=0$ years); (*ii*) the “medium-delay” assumption, where the density-dependent regulation of population parameters relaxed after five years of the simulations ($d_{dens}=5$ years); (*iii*) the “long-delay” assumption, where the density-dependent regulation of population parameters did not relax over the ten years of the simulations ($d_{dens}=10$ years).

***3.3.2. Management***

*Implementation:*

| (4) | When test-and-remove was applied, each individual $i$ in socio-spatial unit $u$ was captured following a Bernoulli process with probability: |
| --- | --- |
|  | $p_{i}\left( t,u \right)=\frac{n_{⚥\left( t,u \right)}}{N_{⚥\left( t,u \right)}}$ |
|  | where $n_{⚥\left( t,u \right)}$ is the number of unmarked males or females $⚥$ to be captured in socio-spatial unit $u$ at time $t$, and $N_{⚥\left( t,u \right)}$ is the total number of unmarked individuals of the corresponding sex-class in the socio-spatial unit $u$ at time $t$. |

The numbers of unmarked individuals captured in each sex-class and in each unit targeted for managements were randomly sampled so that $\sum_{u} n_{⚥\left( t,u \right)}=n_{capt}$. Then, individuals detected as seropositive were removed, whereas individuals detected as seronegative were marked (if not yet the case) and released. $S$ and $E$ individuals were considered as seronegative, whereas $I$ and $R$ individuals were seropositive. Among seropositive individuals, a proportion of $1-Se$ (where $Se$ represents test sensitivity) were mistakenly detected as seronegative.

| (5) | When culling of unmarked individuals was applied, each individual $i$ in socio-spatial unit $u$ was culled following a Bernoulli process with probability: |
| --- | --- |
|  | $p_{i}\left( t,u \right)=\frac{n_{⚥\left( t,u \right)}}{N_{⚥\left( t,u \right)}}$ |
|  | where $n_{⚥\left( t,u \right)}$ is the number of unmarked males or females $⚥$ to be culled in socio-spatial unit $u$ at time $t$, and $N_{⚥\left( t,u \right)}$ is the total number of unmarked individuals of the corresponding sex in socio-spatial unit $u$ at time $t$. |

The numbers of unmarked individuals culled in each sex-class and in each unit targeted for managements were randomly sampled so that $\sum_{u} n_{⚥\left( t,u \right)}=n_{cull}$.

In each case, if the number $n_{X⚥\left( t,u \right)}$ of individuals to be captured or culled in a given socio-spatial unit and sex-class at time $t$ was greater than the number $N_{⚥\left( t,u \right)}$ available, management action was applied only to the available individuals in the socio-spatial unit and sex-class under consideration.

*Rationale:*

We simulated several management strategies, classically applied in wildlife populations and that were used in the past or are under consideration in the studied population: (*i*) “do nothing”, without any management actions, which corresponds to the reference to which the other strategies were compared to; (*ii*) serological testing of live individuals followed by removal (here, mainly euthanasia) of seropositive individuals (“test-and-remove”); and (*iii*) test-and-remove of live individuals combined to culling of others without testing. The simulated strategies representing possible future management options were elaborated in close connection with the ongoing field monitoring and management in order to simulate realistic schemes and parameters.

In our scenarios, only individuals that were never captured before (i.e., unmarked) were targeted for capture or culling, but this could be refined in the future. The model was attributed an objective levels for the total number of individuals to be captured ($n_{capt}=50$) or culled ($n_{cull}=20$) annually. Each year, this total number was randomly distributed among each targeted sex-class and socio-spatial unit, before sampling individuals at random inside each category. In each case, if the number of individuals to be captured or culled in a given socio-spatial unit and sex-class was greater than the number available, management action was applied only to the available individuals in the socio-spatial unit and sex-class under consideration. As a result, the number of treated animals could be below the objective level, by lack of available individuals (representing also the field difficulties that could occur when trying to implement a given strategy).

During captures, seropositive individuals were removed, whereas seronegative ones were marked and released. The sensitivity and specificity of serological tests were assumed to be 95% and 100%, respectively [36]. Although values of sensitivity and specificity of the serological tests that were used in ibex are unknown in this species, such high values were demonstrated when applied to the diagnosis of brucellosis in domestic ruminants [44-46]. Moreover, the serological status in the data of the ibex Bargy population was based on several serological tests performed in parallel, which improves the accuracy of the diagnosis [61].

***3.3.3. Mortality***

*Implementation:*

We used a complete age-dependent model, with a mortality estimate for each age and sex (Toïgo et al. [69] – see Table A1 and Table A3).

| (6) | For each $X$-year old individual $i$, mortality was the outcome of a Bernoulli process with the probability of dying at time $t$ given by: |
| --- | --- |
|  | $p_{i}\left( t \right)=1-\left( 1-\mu_{X⚥} \right)^{\frac{1}{52}}$ |
|  | with $\mu_{X⚥}$ the annual probability of mortality (over 52 weeks) of $X$-year old male or female $⚥$. |

| (7) | For juveniles of socio-spatial unit $u$, mortality was the outcome of a Bernoulli process with the probability of dying at time $t$ given by: |
| --- | --- |
|  | $\left\{ \begin{aligned} p_{i}\left( t,u \right)=1-\left( 1-\mu_{0}\left( u \right) \right)^{\frac{1}{13}}, &\mathrm{if}1\leq t\leq13 \\ p_{i}\left( t,u \right)=0, &\mathrm{if}14\leq t \end{aligned} \right.$ |
|  | with $\mu_{0}$ the probability of winter juvenile mortality (over 13 weeks). |

*Rationale:*

Annual probabilities of natural mortality depended on age and sex, and were derived from data of other ibex populations (Table A3 – Toïgo et al. [63]; Toïgo, pers. comm.). Males lived up to 16 years and females up to 19 years [63].

The density-dependent response of winter juvenile survival was determined using a function that related survival to the carrying capacity and the abundance of the subpopulation (Eq. A1 and Figure Al; [33, 36]). The carrying capacity was estimated in our previous study using Approximate Bayesian Computation [33]. Neonatal mortality was included in the probability to give birth to a live newborn, and therefore the mortality of juveniles was assumed to happen only in winter.

In our model, we assumed no brucellosis-related mortality, as there is currently no evidence for it (no important mortality detected, no seropositive individuals detected in the few individuals found dead).

**Table A3: Values of annual probability of mortality depending on age- and sex-classes.**

|  | $X$ (age in years) | | | | | | | |
| --- | --- | --- | --- | --- | --- | --- | --- | --- |
|  | 0 | 1-8 | 9-12 | 13-15 | 16 | 17 | 18 | 19 |
| $♀$ | Fig. A1 | 0.01 | 0.14 | 0.15 | 0.15 | 0.15 | 0.9 | 1 |
| $♂$ |  | 0.02 | 0.15 | 0.49 | 0.9 | 1 | - | - |

***3.3.4. Spatial movements***

*Implementation:*

| (8) | During mating period, the movement matrix of males was represented as: |
| --- | --- |
|  | $M=\left( \begin{matrix} 1 & 0 & 0 & 0 & 0 \\ 0 & 0.985 & 0.002 & 0.011 & 0.002 \\ 0.008 & 0.049 & 0.908 & 0.016 & 0.019 \\ 0 & 0.002 & 0.027 & 0.918 & 0.053 \\ 0 & 0 & 0.044 & 0.142 & 0.814 \end{matrix} \right)$ |

| (9) | During abortion period, the movement matrix of males was represented as: |
| --- | --- |
|  | $M=\left( \begin{matrix} 0.988 & 0 & 0.010 & 0.002 & 0 \\ 0 & 0.981 & 0.013 & 0.006 & 0 \\ 0 & 0.017 & 0.925 & 0.057 & 0.001 \\ 0 & 0 & 0.020 & 0.979 & 0.001 \\ 0 & 0 & 0 & 0.028 & 0.972 \end{matrix} \right)$ |
|  | where $m_{i,j}^{♂}$, the element in $i^{th}$ row and $j^{th}$ column of the movement matrix, is the weekly probability $p_{ij}^{'}$ for a male whose permanent unit is $i$ to temporarily visit the unit $j$ at the time step $t$. Thus, for a male whose permanent socio-spatial unit is $u \left( u\in\left[ 1;5 \right] \right)$, the outcome of the multinomial process to determine the temporary socio-spatial unit at time $t$ depended on the vector of probability $p_{i}\left( t,u \right)=m_{u,*}^{♂}$, where $m_{u,*}^{♂}$ represents all the elements of the $u^{th}$ row of the movement matrix. |

*Rationale:*

Using GPS hourly locations from 51 females and 39 males, we estimated the probability that an ibex belonging to one socio-spatial unit moved to another one. The probability for an ibex whose permanent socio-spatial unit is $i$ to visit the socio-spatial unit $j$, and therefore to engage in potentially infectious contacts, was calculated as [64]:

$$p_{ij}=\frac{\sum_{k=1}^{n_{i}} R_{ijk}}{n_{i}}$$

$R_{ijk}$ was set to 1 if the $k^{th}$ hourly location of an ibex from unit $i$ was in unit $j$ and 0 otherwise, and $n_{i}$ was the total number of hourly locations for all ibex from unit $i$ during the period considered. For females, this probability was always $p_{ii}=1$, and $p_{ij}=0$ for $j\neq i$, thus females stayed in their socio-spatial unit lifelong. Thus, we assumed that females were faithful to their permanent unit and did not engage in any movements between units.

Males, however, were found to move between units during the periods when they can transmit or acquire infection, i.e., during mating period in winter, and in spring when infectious abortions due to *Brucella* occur. The probability and direction of movements were estimated separately for the two periods, because males are especially likely to visit other units during the mating period [31]. Then, we converted these probabilities $p_{ij}$ over the duration of the mating period or the abortion period in the corresponding weekly probabilities of movements at each time step $p_{ij}^{'}$ for the two periods (Figure A3).

We considered all movements to be temporary. Thus, in the model, at each time step, males either stayed in their permanent socio-spatial unit or moved to another socio-spatial unit, with probabilities depending on permanent socio-spatial unit.

*
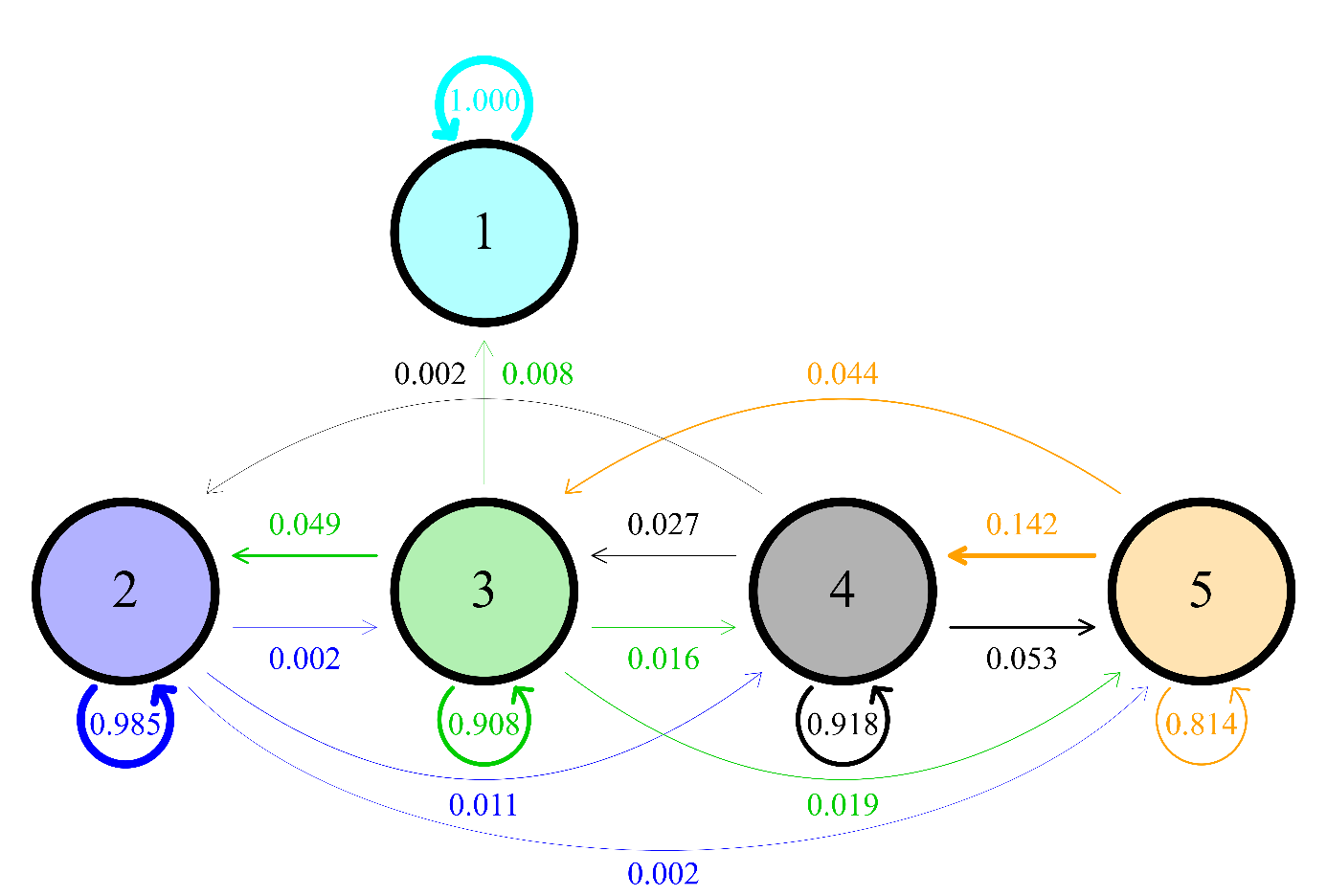
*

(A)

*
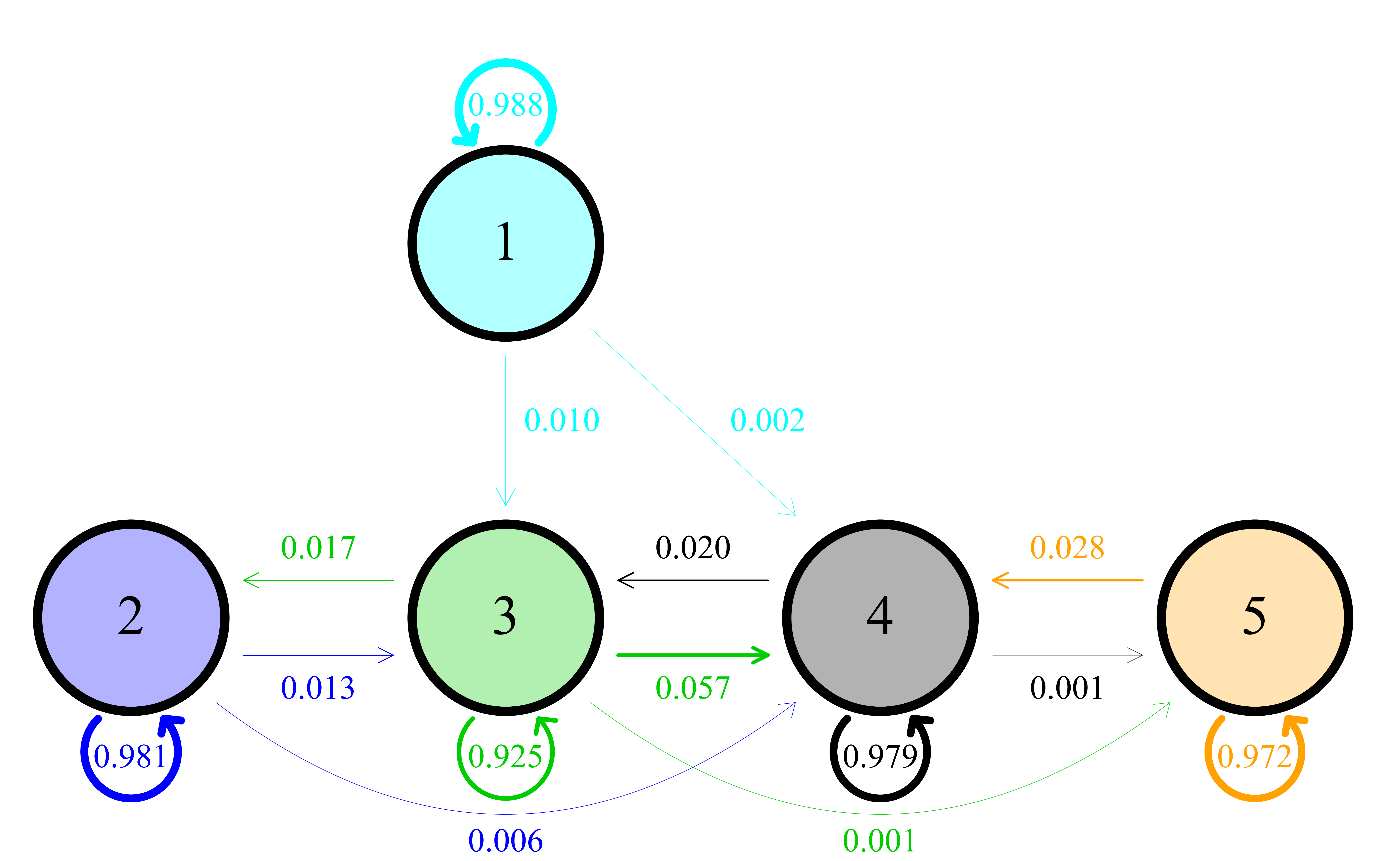
*

(B)

**Figure A3: Weekly probabilities of between-units movements for males during mating (A) and abortion (B) periods.**

Arrows represent movements and are drawn with the colour of the permanent socio-spatial unit (sky blue: Leschaux-Andey, navy blue: Charmieux-Buclon, green: Jallouvre-Peyre, black: Grand Bargy, orange: Petit Bargy). Arrows are proportional to the probability of movements, which are also explicitly specified for each movement.

***3.3.5. Reproduction***

*Implementation:*

For each female, becoming sexually receptive during mating was the outcome of a Bernoulli process.

| (10) | For each $S$, $E$, and $R$ female $i$ in socio-spatial unit $u$, becoming sexually receptive during mating was the outcome of a Bernoulli process with the probability: |
| --- | --- |
|  | $p_{i}\left( u \right)=\tau_{X}\left( u \right)$ |
|  | with $\tau_{X}$ the probability of being sexually receptive for a $X$-year old female. |

| (11) | For each $I$ female $i$ in socio-spatial unit $u$, becoming sexually receptive during mating was the outcome of a Bernoulli process with the probability: |
| --- | --- |
|  | $p_{i}\left( u \right)=\zeta_{\tau}\times\tau_{X}\left( u \right)$ |
|  | with $\tau_{X}$ the probability of being sexually receptive for an $X$-year old female and $\zeta_{\tau}$ the impact of brucellosis on sexual receptivity probability. |

| (12) | Sexual receptivity only lasted for one weekly time step. The week of receptivity was the outcome of a trial with the following probability distribution: |
| --- | --- |
|  | $\left\{ \begin{aligned} p\left( week=t \right)=\frac{p_{1}}{2}, &\mathrm{if}1\leq t\leq2 \\ p\left( week=t \right)=\frac{p_{2}}{d_{rut}-2}, &\mathrm{if}3\leq t\leq d_{rut} \end{aligned} \right.$ |
|  | with $p_{1}$ the proportion of associations during the first 2 weeks of the mating period, $p_{2}$ the proportion of associations during the rest of the mating period, and $d_{rut}$ the duration of the mating period. |

For each receptive female, $n_{T}$ tending males and $n_{C}$ coursing males were sampled for mating association (13-14):

| (13) | Tending males were sampled among all available tending males present in the same socio-spatial unit $u$ as the female at time $t$, following a uniform distribution: for each individual $i$, the probability to be associated with this female was: |
| --- | --- |
|  | $p_{i}\left( t,u \right)=\frac{1}{N_{T\left( t,u \right)}}$ |
|  | where $N_{T\left( t,u \right)}$ is the number of available tending males in unit $u$ at time $t$. |

| (14) | Coursing males were sampled following a bimodal distribution according to age. For each individual $i$ in socio-spatial unit $u$, the probability to be associated with this female was: |
| --- | --- |
|  | $\left\{ \begin{aligned} p_{i}\left( t,u \right)=\frac{p_{C1}}{p_{C1}\times N_{C1\left( t,u \right)}+p_{C2}\times N_{C2\left( t,u \right)}}, &for coursing males \leq5 years old \\ p_{i}\left( t,u \right)=\frac{p_{C2}}{p_{C1}\times N_{C1\left( t,u \right)}+p_{C2}\times N_{C2\left( t,u \right)}}, &for coursing males 6-7 years old \end{aligned} \right.$ |
|  | where $p_{C1}$ and $p_{C2}$ are the proportion of coursing males under 5 years old and 6-7 years old associated to females, and $N_{C1\left( t,u \right)}$ and $N_{C2\left( t,u \right)}$ are the number of available coursing males under 5 years old and 6-7 years old in unit $u$ at time $t$, respectively. |
| (15) | Only one male among those associated to a receptive female engaged in copulation. A single male was randomly selected, based on the probabilities: |
|  | $\left\{ \begin{aligned} p_{i}\left( t \right)=\frac{\tau_{T}}{n_{T}}, &for tending males \\ p_{i}\left( t \right)=\frac{\tau_{C}}{n_{C}}, &for coursing males \end{aligned} \right.$ |
|  | where $\tau_{T}$ and $\tau_{C}$ are copulation probabilities of tending males (8-14 years old) and coursing males (2-7 years old) respectively, and $n_{T}$ and $n_{C}$ the number of tending and coursing males respectively associated to the female considered. |

Every receptive female who engaged in sexual intercourse with a male became pregnant.

*Rationale:*

In the model, reproductive success of females was the outcome of two processes: sexual receptivity during mating, and the probability to give birth to a live newborn. In ibex, most mature females are sexually receptive only once during the mating period [52]. We considered that a mature female could become sexually receptive during mating only once every year with probabilities depending on age (starting at 1.5 years old [51]). The density-dependent response of female receptivity was determined using a function that related receptivity to the carrying capacity and the abundance of the subpopulation (Eq. A2-3 and Figure A1; [33, 36]). In our model, we assumed that *Brucella* infection decreased sexual receptivity during mating, thus leading to a lower probability of gestation (see Table A1) based on data from the study population and by analogy with domestic ruminant infection [36].

Males, on the other hand, are sexually mature at 2.5 years of age and active every year throughout the mating period [59]. Sexual receptivity in females is short, less than two days [52], so we assumed that mature males could be associated with up to 3 females per week (Table A1).

Males adopt different mating tactics according to their dominance status. Subordinate males adopt mainly the coursing tactic, whereas dominant males adopt the tending tactic [52, 59]. Tending males monopolise access to the female, whereas coursing males are more opportunistic and try to access the female temporarily when it starts to run [52]. The adoption of one of the two tactics is strongly age-dependent. Younger males (2-7 years) mainly adopt the coursing tactic while older males (≥ 8 years) mainly engage in tending [52, 59]. Accordingly, in the model, we made the simple assumption that all males aged 2-7 years were coursing while all males aged 8-14 years were considered as tending.

During mating, a receptive female is associated with one tending male and a median of three coursing males [52]. In the model, we associated a receptive female with up to four males: one tending male and three coursing males, randomly sampling males among those that were not already associated to another female and depending on age. Moreover, among coursing males, those between 2 and 5 years old were around twice less frequent than males of 6 and 7 years of age (Eq. A14 – 4/67 versus 3/23 – Table 3 in [59]).

In ibex, only one male succeeds in engaging sexual intercourse with a given female [52]. Based on paternity data, males that we considered as tending (≥ 8 years) are around 6 times more successful in engaging sexual intercourse with the female than coursing males (Eq. A15 – 14/32 *versus* 7/90 – Table 3 in [59]). We used these probabilities in the model to sample the successful male among those that were associated to the receptive female. As a result of these two processes (association to a receptive female and selection of the successful male), offspring were sired by a small fraction of males, mainly dominant males using the tending tactic [59].

***3.3.6. Venereal transmission***

*Implementation:*

We considered that all actively infected males and females $I$ shed the bacteria during mating, and could therefore be responsible for venereal transmission, until they became $R$ (non-actively infected), based on the probability of recovery $\gamma$.

| (16) | For male-to-female transmission, a $S$ female $i$ engaged in sexual intercourse with a $I$ male became $E$ following a Bernoulli process with probability: |
| --- | --- |
|  | $\left\{ \begin{aligned} p_{i}\left( t \right)=\nu_{ven}, &for a female with a tending male \\ p_{i}\left( t \right)=\kappa\times\nu_{ven}, &for a female with a coursing male \end{aligned} \right.$ |
|  | with $\nu_{ven}$ the probability of successful venereal transmission from tending males to females given contact, and $\kappa$ the relative efficacy of coursing male-to-female transmission. |

| (17) | For female-to-male transmission, a $S$ male $i$ engaged in sexual intercourse with a $I$ female became $E$ following a Bernoulli process with probability: |
| --- | --- |
|  | $\left\{ \begin{aligned} p_{i}\left( t \right)=\omega\times\nu_{ven}, &for a tending male \\ p_{i}\left( t \right)=\omega\times{\kappa\times\nu}_{ven}, &for a coursing male \end{aligned} \right.$ |
|  | with $\nu_{ven}$ the probability of successful venereal transmission from tending males to females given contact, $\omega$ the relative efficacy of female-to-male transmission, and $\kappa$ the relative efficacy of coursing male-to-female transmission. |

*Rationale:*

The probability of successful venereal transmission given contact was estimated in our previous study using Approximate Bayesian Computation [33].

During sexual intercourse with a female, a tending male often manages to copulate several times due to its almost exclusive access, contrary to a coursing male (2.22 copulations on average for tending males vs. 1 copulation for coursing males; [52]). Therefore, in the model, the probability of venereal transmission during a sexual intercourse between an infectious male and a susceptible female or vice versa was supposed to be higher for tending than for coursing males (Table A1).

Venereal transmission of *Brucella abortus* experimentally-infected rats appeared more efficient from males to females [65], and male-to-female transmission is more efficient than female-to-male in several other sexually-transmitted diseases [60]. Therefore, the efficacy of male-to-female transmission was assumed to be 1.67 times higher than female-to-male transmission (Table A1; [60]).

***3.3.7. Abortions or births***

*Implementation:*

Only sexually receptive female who engaged in sexual intercourse with a male became pregnant. Females that were not sexually receptive or that did not engage in sexual intercourse were considered as not pregnant.

The possibility of an abortion to occur depended on females’ parity and transmission route. We considered that pregnant females infected by horizontal transmission caused by infectious abortions or births of other females during the same gestation did not abort, as infection was acquired during late gestation [55]. In the case of females infected by horizontal transmission caused by infectious abortions or births before pregnancy, abortion could occur only on primiparous females. For all other transmission routes (venereal, congenital and pseudo-vertical transmission), abortion could occur during the first pregnancy post-infection (Figure A4).

| (18) | For pregnant $I$ females infected for the first time during mating period, for the first pregnancy of $I$ females vertically infected, and for $I$ primiparous females infected before pregnancy (horizontally or pseudovertically), the occurrence of infectious abortion (IA) due to *Brucella* was the outcome of a Bernoulli process with probability: |
| --- | --- |
|  | $p_{i}\left( t \right)=\rho$ |
|  | with $\rho$ the probability of abortion during the first pregnancy following infection. |

If abortion did not occur for those females, their probability of giving birth was 1 and associated births were considered as infectious births (IB).

| (19) | In subsequent gestations of $I$ females, as well as for $I$ primiparous females infected horizontally during gestation, and for the first gestation following horizontal infection in $I$ multiparous females, giving birth was the outcome of a Bernoulli process with probability: |
| --- | --- |
|  | $\left\{ \begin{aligned} p_{i}\left( t \right)=\zeta_{\eta}\times\eta_{1}, &for primiparous females \\ p_{i}\left( t \right)=\zeta_{\eta}\times\eta_{2}, &for multiparous females \end{aligned} \right.$ |
|  | with $\eta_{1}$ the probability of giving birth for primiparous females, $\eta_{2}$ the probability of giving birth for multiparous females, and $\zeta_{\eta}$ the impact of brucellosis on probability of giving birth of infectious females. |

Associated births were considered as infectious births.

| (20) | For $S$ and $R$ pregnant females, giving birth was the outcome of a Bernoulli process with probability: |
| --- | --- |
|  | $\left\{ \begin{aligned} p_{i}\left( t \right)=\eta_{1}, &for primiparous females \\ p_{i}\left( t \right)=\eta_{2}, &for multiparous females \end{aligned} \right.$ |
|  | with $\eta_{1}$ the probability of giving birth for primiparous females, and $\eta_{2}$ the probability of giving birth for multiparous females. |

For these females, births were not associated with *Brucella* shedding. $S$ and $R$ pregnant females that did not give birth to a live newborn were not associated with *Brucella* shedding either.

The sex of each newborn was the outcome of a Bernoulli process with the probability $\delta$, the proportion of female at birth.

*Rationale:*

In domestic ruminants, 80% of infectious females abort and most of them do so only once, during the first pregnancy post-infection [47, 61]. If abortion does not occur, females give birth to a live newborn and invasion of the uterus leads to *Brucella* shedding in genital fluids at the time of parturition [55]. In both cases (abortion or not), females shed *Brucella* in genital fluids for *ca.* three weeks.

For other pregnant females, and for actively infected females that already went through their first pregnancy following infection, the probability to give birth to a live newborn that survives until winter depends on their parity (Table A1). We also assumed that active *Brucella* infection decreases the probability to give birth to a live newborn (Table A1). Actively infected females that give birth to a live newborn shed *Brucella* in genital fluids for three weeks. The sex-ratio at birth was expected to be 1.


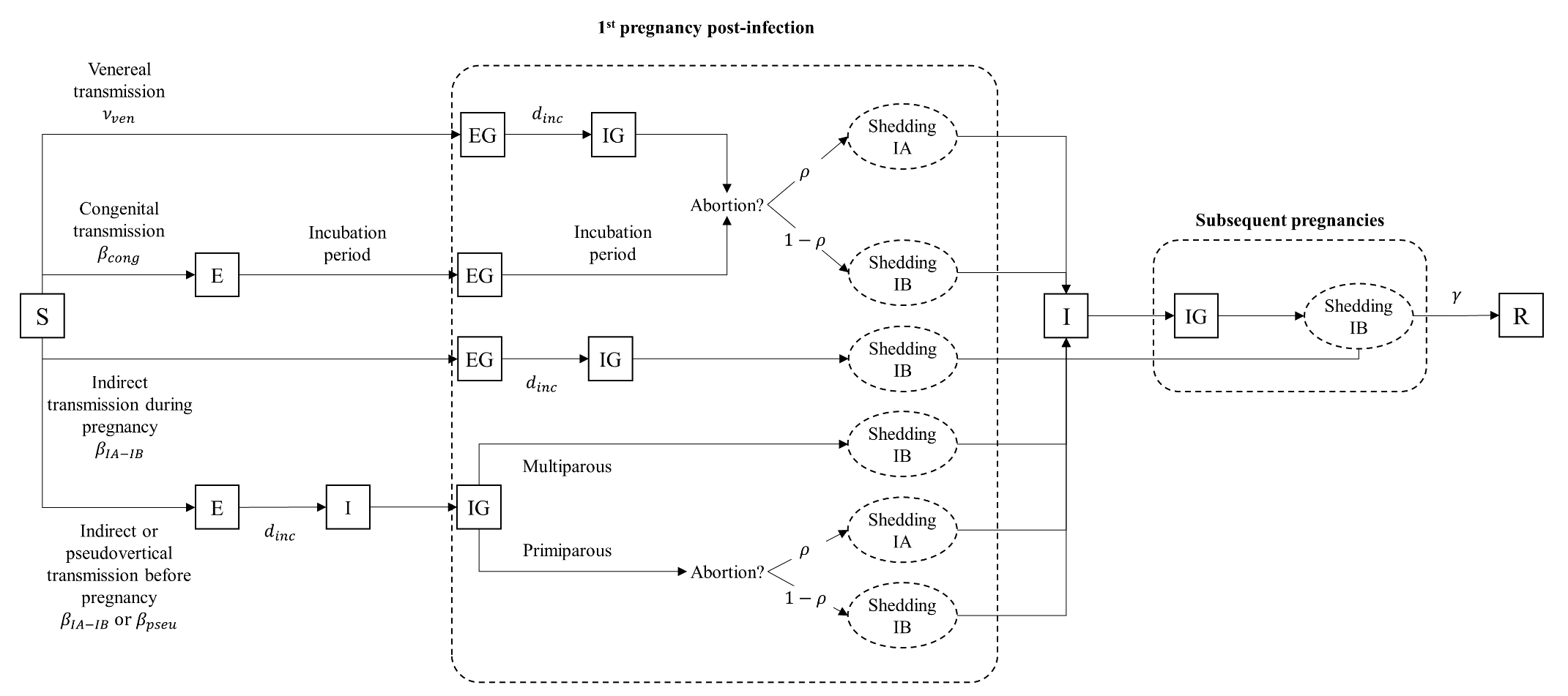


**Figure A4: Changes in disease state and shedding status of females depending on transmission routes and parity.**

Squares represent health states: *S,* susceptible to infection, *E*, exposed, *I*, actively infected, *R*, non-actively infected (non-shedders), *G*, pregnant. Solid horizontal arrows represent flows between compartments. Dashed circles represent the consequences of *B. melitensis* infection on urogenital shedding through infectious abortion (IA) or through infectious birth (IB). Parameters are: $\nu_{ven}$ the probability of successful venereal transmission given contact, $\beta_{cong}$ the congenital transmission coefficient by in utero infection, $\beta_{IA-IB}$ the per capita probability of one ibex coming into effective contact with one infectious abortion or birth, $\beta_{pseu}$ the pseudo-vertical transmission coefficient by milk ingestion, $d_{inc}$ the duration of *Brucella* incubation, $\rho$ is the probability of abortion during the first pregnancy following infection, and $\gamma$ is the probability of recovery.

***3.3.8. Vertical transmission***

*Implementation:*

All newborns were $S$, except for newborns of $I$ females who became $E$ through congenital in utero transmission as a result of a Bernoulli process with probability $\beta_{cong}$. Remaining $S$ newborns of $I$ females were exposed to *Brucella* shedding in colostrum and milk, and became $E$ through this pseudo-vertical route of transmission as a result of a Bernoulli process with probability $\beta_{pseu}$.

| (21) | For $S$ kids born from an $I$ female in the interval $\left[ t-d_{isol}+1;t \right]$, being infected with the Infectious Birth (IB) of their mother and becoming $E$ was the outcome of a Bernoulli process with probability: |
| --- | --- |
|  | $p_{i}\left( t \right)=\beta_{IB}$ |
|  | with $\beta_{IB}$ the per capita probability of one female coming into effective contact with one infectious birth (per week), and $d_{isol}$ the duration of postpartum isolation of the mother-offspring couple. |

*Rationale:*

Congenital transmission from infected mothers to their offspring in utero is demonstrated in domestic ruminants [66] and happens in *ca.* 5% of kids born from infectious mothers [47]. Pseudo-vertical transmission from infectious mothers to kids can also happen through colostrum or milk consumption in all gestation events [55, 67], but this transmission only concerns a small proportion of kids [68]. Both transmission routes are suspected in Alpine ibex [32] and were included in the model, with fixed low probabilities of 5% by analogy with domestic ruminants (Table A1; [33, 36]).

During the two weeks following parturition, we considered that an actively infected female that shed *Brucella* in genital fluids could only transmit infection to its newborn, which we considered as a special case of pseudo-vertical transmission because ibex females isolate themselves with their newborns during parturition time [53].

***3.3.9. Horizontal transmission***

*Implementation:*

| (22) | For $S$ individuals in socio-spatial unit $u$ at time $t$, being infected with Infectious Abortions (IA) of the same unit and becoming $E$ was the outcome of a Bernoulli process with probability: |
| --- | --- |
|  | $\left\{ \begin{aligned} p_{i}\left( t,u \right)=1-\left( 1-\beta_{IA} \right)^{\sum IA\left( u,t \right)}, &for females \\ p_{i}\left( t,u \right)=1-\left( 1-\sigma\times\beta_{IA} \right)^{\sum IA\left( u,t \right)}, &for males \leq5 years old \\ p_{i}\left( t,u \right)=1-\left( 1-\phi\times\beta_{IA} \right)^{\sum IA\left( u,t \right)}, &for males>5 years old \end{aligned} \right.$ |
|  | with $\beta_{IA}$ the per capita probability of one female coming into effective contact with one infectious abortion (per week), $\sigma$ the reduction in the number of contacts with infectious abortion for males 5 years of age and under, $\phi$ the reduction in the number of contacts with infectious abortion for males over 5 years of age, $IA(u,t)$ the number of females in socio-spatial unit $u$ alive at time $t$ which aborted in the interval $\left[ t-d_{shed}+1;t \right]$ and $d_{shed}$ the duration of shedding of *Brucella* in genital fluids after infectious birth or abortion. |

| (23) | For $S$ adult females who gave birth before $t-d_{isol}+1$, or for $S$ newborn males or females born before $t-d_{isol}+1$, being infected with Infectious Births (IB) and becoming $E$ was the outcome of a Bernoulli process with probability: |
| --- | --- |
|  | $p_{i}\left( t \right)=1-\left( 1-\beta_{IB} \right)^{\sum IB(u,t)}$ |
|  | with $\beta_{IB}$ the per capita probability of one female or newborn coming into effective contact with one infectious birth (per week), $IB(u,t)$ the number of females in socio-spatial unit $u$ alive at time $t$ which gave birth in the interval $\left[ t-d_{shed}+1;t-d_{isol} \right]$, $d_{shed}$ the duration of shedding of *Brucella* in genital fluids after infectious birth or abortion, and $d_{isol}$ the duration of postpartum isolation of the mother-offspring couple. |

*Rationale:*

In domestic ruminants, excretion of the *Brucella* in female genital discharges through infectious abortions or births is considered as the main route of transmission to other individuals [55]. For this transmission route, contacts were not modelled explicitly. An individual was exposed to all abortions or births caused by brucellosis within the same socio-spatial unit. We assumed that at a given time step, the probability of an individual coming into effective contact (i.e., coming into a contact that leads to infection) with one infectious abortion or with one infectious birth was the same (Table A1). This probability was estimated in our previous study using Approximate Bayesian Computation [33].

In ibex, males over 5 years of age segregate from females both socially and spatially soon after the rut, whereas segregation is more gradual for males 5 years of age and under, which are still associated to females during spring [53]. Thus, we assumed in the model that males under 5 were less exposed than females to *Brucella* shed following infectious abortions, and males over 6 even less (Table A1). After being isolated for two weeks following parturition, females and kids gather to form nurseries [51], and have different space use than males older than 1 year old and females without kids [69, 70]. Thus, we considered that mothers began to gather in nurseries three weeks after parturition. Horizontal transmission through infectious births was assumed to occur only in nurseries, from infected mothers to non-infected mothers and susceptible newborns.

***3.3.10. Incubation and recovery***

*Implementation:*

After $d_{inc}$ weeks of incubation, $E$ individuals became $I$, except for $E$ individuals infected through *in utero* transmission, as infection remained latent in these individuals and they stayed in state $E$ up to the first abortion or birth for females, and up the age of sexual maturity in males.

| (24) | Then, at each time step, the probability for an $I$ individual $i$ to become $R$ was: |
| --- | --- |
|  | $p_{i}\left( t \right)=1-\left( 1-\gamma\right)^{\frac{1}{52}}$ |
|  | with $\gamma$ the annual probability of recovery. |

*Rationale:*

Transmission of *Brucella* to a susceptible ibex $S$ led to an incubation (state $E$) of three weeks (Table A1), which is the duration of the incubation period after which seroconversion generally occurs in domestic ruminants [55]. For kids born after congenital transmission, the incubation ends only at abortion or parturition after their first pregnancy for females or at the age of sexual maturity for males [66]. After incubation, we considered that infected individuals first entered the $I$ class, characterised by an active infection and therefore the ability to shed the bacteria.

Bacteriological analyses revealed that the probability of active infection decreases with increasing age [32]. Therefore, we assumed that $I$ individuals could transit to the $R$ class, i.e., infected but without active infection and unable to shed the bacteria, based on the probability of recovery. This probability of recovery $\gamma$ was estimated from data on bacteriological cultures in seropositive ibex of the study site [32].
