# Supplementary material for: Targeted strategies for the management of wildlife diseases: the case of brucellosis in Alpine ibex
Source: Vet Res. 2021 Sep 14;52:116. doi: 10.1186/s13567-021-00984-0 (PMC8439036; doi:10.1186/s13567-021-00984-0)
Supplement: Supplementary file 2 — Additional file 2. Sensitivity analysis. [33, 36, 38, 39, 44–47, 51, 52, 55, 60, 66, 71–73] This file contains the framework and detailed results of the sensitivity analysis. [file 13567_2021_984_MOESM2_ESM.docx]

**ADDITIONAL FILE 2: Sensitivity analysis**

**1. Choice of model outputs**

The outputs considered in the sensitivity analysis were the same as the outputs used to compare management strategies based on their effect on *Brucella* *melitensis* infection and their impact on the ibex population in terms of number of animal treated and population size. These outputs were: (*i*) the seroprevalence at the end of the simulations, which is as a proxy of infection in the population and can be directly compared with field data; (*ii*) the proportion of simulations where *Brucella* was no longer present at the end of the simulation (no remaining infectious ibex), used as a probability of extinction of *B. melitensis* in this population, which would be a desirable goal; (*iii*) the population size at the end of the simulations; and the numbers of individuals (*iv*) captured, (*v*) removed (during test-and-remove protocol), and (*vi*) culled (without testing) after ten years of simulations.

**2. Choice of parameters**

Most epidemiological and demographic parameters in Lambert et al. [33] were calibrated by using published data on brucellosis in domestic ruminants, experts’ knowledge and unpublished data on brucellosis in ibex [36], and knowledge from published and unpublished data on ibex demography and behaviour in the study site or others (see Additional File 1).

As sensitivity analysis can be quite computationally expensive and our model was complex, we wanted to reduce the number of parameters to include in the sensitivity analysis to keep a reasonable number of simulations.

Based on our choice of model outputs, we considered parameters relating to the following model processes to be the most influential *a priori*: density-dependent regulation of population parameters, management interventions, venereal transmission, vertical transmission, horizontal transmission, incubation and recovery (see Additional File 1 for processes description). To prioritize parameters for inclusion in the sensitivity analysis, we classified these parameters in two categories, depending on the degree of available knowledge on these parameters and their potential influence on model outputs (Table A1).

Among parameters in the first category, the most uncertain one was the delay between the beginning of the simulations and the relaxation of the density-dependent regulation of population parameters. Four parameters (the carrying capacity, the per-capita probability of one individual coming into effective contact with one infectious abortion or birth per week, the probability of successful venereal transmission given contact between an infectious and a susceptible host, and the probability of recovery) were estimated in our previous study [33]. Nonetheless, they were included because of the remaining uncertainty in their distribution and their likely impact on the selected outputs.

**Table A1: Definition of a subset of parameters, classified in two categories based on their uncertainty/potential influence.**

| Symbol | Description (dimension) | Value | References | Category |
| --- | --- | --- | --- | --- |
| $d_{dens}$ | Delay between the beginning of the simulations and the relaxation of the density-dependent regulation (years) | 0; 5; 10 | * | 1 |
| $K$ | Carrying capacity of the metapopulation (individuals) |  | † | 1 |
| $d_{man}$ | Duration of management interventions (years) | 10 | ‡ | 1 |
| $n_{capt}$ | Objective level for the total number of individuals to be captured (per year) | 50 | ‡ | 1 |
| $n_{cull}$ | Objective level for the total number of individuals to be culled (per year) | 20 | ‡ | 1 |
| $Se$ | Sensitivity of serologic tests | 0.95 | [36] | 1 |
| $\nu_{ven}$ | Probability of successful venereal transmission from tending males to females given contact |  | † | 1 |
| $\beta_{cong}$ | Congenital transmission probability by *in utero* infection | 0.05 | [47] | 1 |
| $\beta_{pseu}$ | Pseudo-vertical transmission probability by milk ingestion | 0.05 | * | 1 |
| $\beta_{IA}=\beta_{IB}$ | Per capita probability of one female coming into effective contact with one infectious abortion or birth (per week) |  | † | 1 |
| $\gamma$ | Probability of recovery (annual) | 0.16 | § | 1 |
| $k$ | Threshold for density-dependent effect (individuals) | 131 | * | 2 |
| $\kappa$ | Relative efficacy of venereal transmission from coursing males | 0.45 | [52] | 2 |
| $\omega$ | Relative efficacy of female-to-male venereal transmission | 0.60 | [60] | 2 |
| $d_{isol}$ | Duration of postpartum isolation of the mother-offspring couple (weeks) | 2 | [51] | 2 |
| $d_{shed}$ | Duration of shedding of *Brucella* in genital fluids when a female aborts or gives birth (weeks) | 3 | [55] | 2 |
| $\sigma$ | Reduction in the number of contacts with infectious abortion for males ≤5 years old | 0.27 | § | 2 |
| $\phi$ | Reduction in the number of contacts with infectious abortion for males >5 years old | 0.06 | § | 2 |
| $d_{inc}$ | Duration of *Brucella* incubation (weeks) | 3 | [55] | 2 |

* Experts knowledge

† Accepted parameter values in the Approximate Bayesian Computation [33]

‡ Tailored to represent realistic management interventions in the study population

§ Calibrated using field data

The probabilities for the two remaining transmission routes, congenital and pseudo-vertical transmission, were also included as they are likely to impact the outputs. In particular, individuals congenitally infected seroconvert only at abortion or parturition after their first pregnancy for females or at the age of sexual maturity for males [66]. This could play an important role in the persistence of *B. melitensis* in the ibex population, as observed in domestic ruminants [66].

Finally, all parameters related to management interventions were included in the first category, as we obviously expect them to impact the outputs of our model. These parameters were the duration of management interventions, the objective levels for the total number of individuals to be captured or culled annually, and the sensitivity of serological tests used for test-and-remove protocols during captures.

Other parameters were included in the second category as they were considered as less uncertain based on published literature and available data or as less likely to impact the model outputs. Thus, we were left with eleven parameters, that we considered as the most uncertain and potentially the most influential on the outputs of our model (Table A2).

**Table A2: Parameter values used in the factorial design of the sensitivity analysis.**

|  | Range | Range justification | Values tested in the sensitivity analysis | | |
| --- | --- | --- | --- | --- | --- |
| $d_{dens}$ | [0 - 10] | * | 0 | 5 | 10 |
| $K$ | [535 - 591] | † | 535 | 564 | 591 |
| $d_{man}$ | [5 - 10] | ‡ | 5 | 7.5 | 10 |
| $n_{capt}$ | [0 - 50] | ‡ | 0 | 25 | 50 |
| $n_{cull}$ | [0 - 20] | ‡ | 0 | 10 | 20 |
| $Se$ | [0.75 - 1] | *; [36, 44–46] | 0.75 | 0.875 | 1 |
| $\nu_{ven}$ | [0.005 - 0.682] | † | 0.005 | 0.168 | 0.682 |
| $\beta_{cong}$ | [0 - 0.10] | *; [47] | 0 | 0.05 | 0.10 |
| $\beta_{pseu}$ | [0 - 0.10] | * | 0 | 0.05 | 0.10 |
| $\beta_{IA}=\beta_{IB}$ | [0.001 - 0.128] | † | 0.001 | 0.026 | 0.128 |
| $\gamma$ | [0.11 - 0.22] | § | 0.11 | 0.16 | 0.22 |

* Experts knowledge

† 95% credible interval (Approximate Bayesian Computation); Lambert et al. [33]

‡ Tailored to represent realistic management interventions in the study population

§ 95% confidence interval (calibrated using field data); Lambert et al. [33]

**3. Sensitivity analysis design**

To perform sensitivity analysis on these eleven parameters, we used a fractional factorial plan – FFP. We used 3 levels (values) per parameter: the median, minimum and maximum of their range values (Table A2). Using planor R package [71], we generated a fractional factorial plan of resolution V, which allowed us to estimate all of the main effects and first order interactions assuming that higher order interactions are negligible [72, 73].

This design represented $3^{5}=243$ scenarios (one scenario was a set of parameter values). As our model was stochastic, 1000 iterations were performed per scenario, and the median and variance of each simulated outputs were retrieved (except for the proportion of simulations where *Brucella* was extinct, which by definition takes a single value per scenario). This number of iterations allowed us to obtain convergence of all simulated outputs (not shown).

We performed a global sensitivity analysis on simulated outputs, implemented in the multisensi R package [38, 39]. We applied global sensitivity analysis after multivariate dimension reduction on all simulated outputs using principal component analysis – PCA [38, 39]. For each parameter, generalized sensitivity indexes $GSI$ were calculated and were represented by Pareto plots.

For comparison purposes, the global sensitivity analysis was repeated four times, for each targeted scenario: (*i*) untargeted management interventions; (*ii*) management interventions targeted towards the socio-spatial units of the core area of the massif (“core”); (*iii*) management interventions targeted towards females (“female”); or (*iv*) management interventions targeted towards females of the core area (“corefemale”).

**4. Sensitivity analysis results**

Seven main parameters stood out with generalized sensitivity indices over 0.05 for each targeted scenarios and over 0.20 for at least one of the six outputs (Figures A1-5):

1. $n_{capt}$: the objective level for the total number of individuals to be captured annually.
2. $\beta_{IA}=\beta_{IB}$: the per-capita probability of one host coming into effective contact with one infectious abortion or birth per week.
3. $n_{cull}$: the objective level for the total number of individuals to be culled annually.
4. $d_{dens}$: the delay between the beginning of the simulations and the relaxation of the density-dependent regulation of population parameters.
5. $d_{man}$: the duration of management interventions.
6. $\nu_{ven}$: the probability of successful venereal transmission given contact between an infectious and a susceptible host.
7. $K$: the carrying capacity.

Global sensitivity analyses for each output separately are given in Figures A2-5. Results were qualitatively and quantitatively similar for each targeted scenarios (Figures A2-5). Results were also similar when performing the sensitivity analysis on only the mean of the simulated outputs, only their variance, or both (not shown).

| 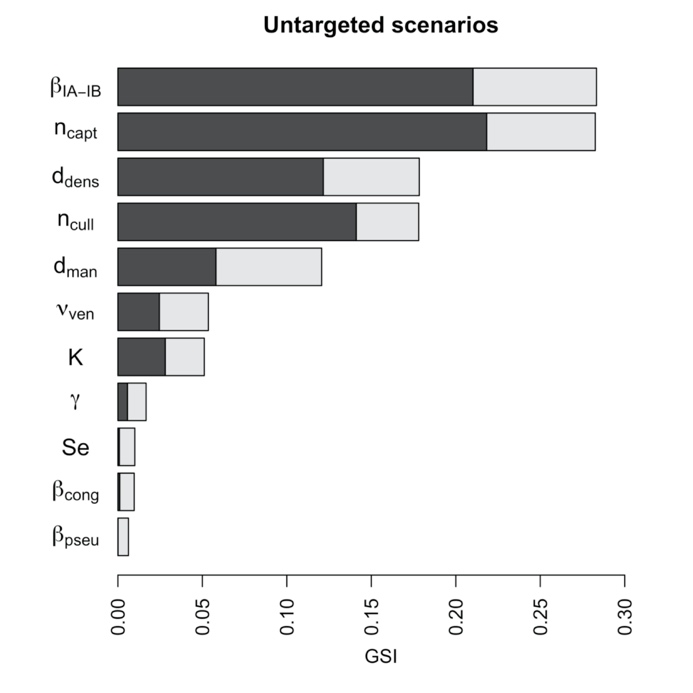 | 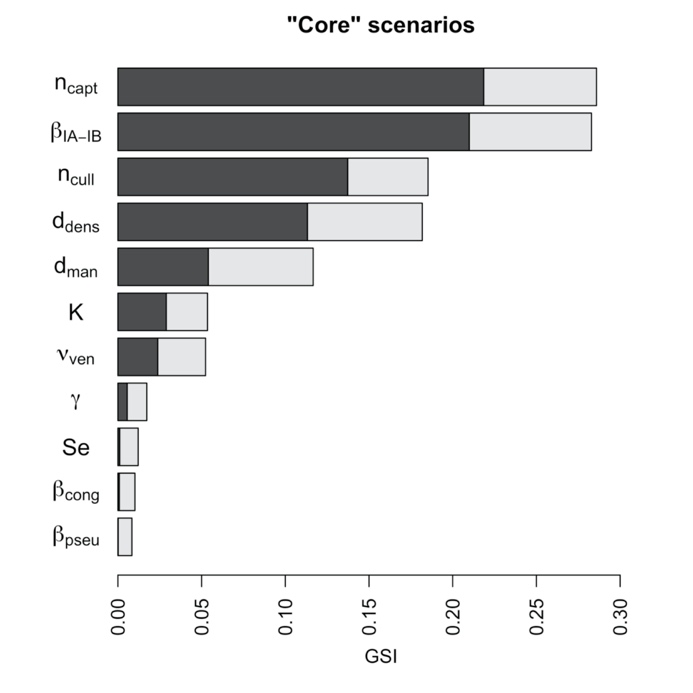 |
| --- | --- |
| 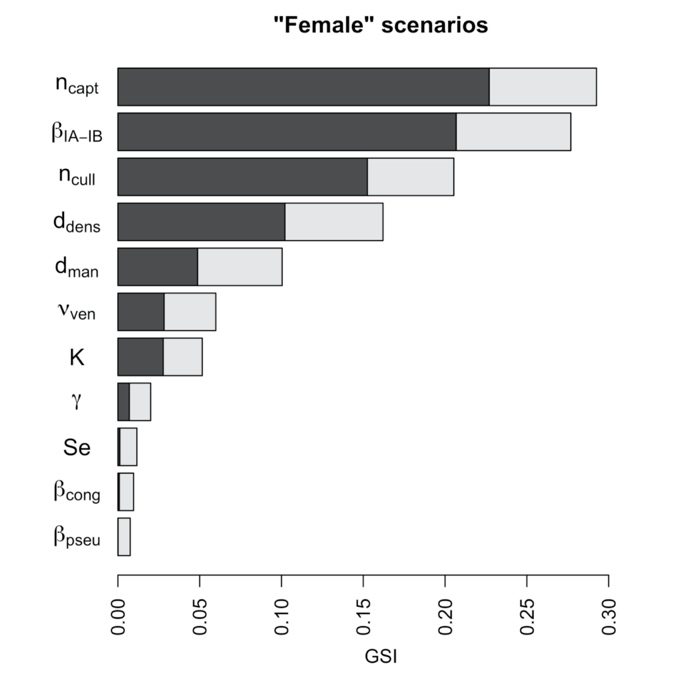 | 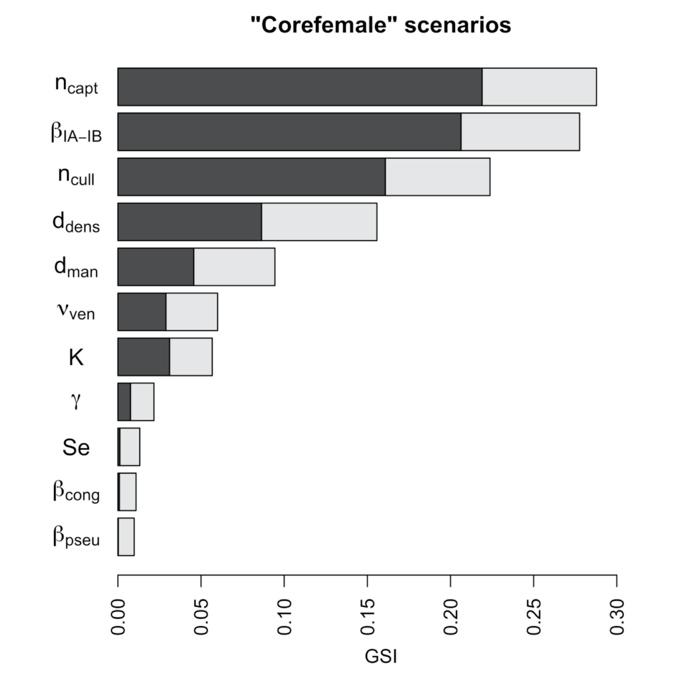 |

**Figure A1: Comparison of the generalized sensitivity indices for all simulated outputs for each targeted scenario.**

The main sensitivity indices are in dark bars and interaction ones are in pale bars. The total length represents the total sensitivity index. Targeted scenarios were: (*i*) untargeted management interventions (“untargeted”); (*ii*) management interventions targeted towards the socio-spatial units of the core area of the massif (“core”); (*iii*) management interventions targeted towards females (“female”); or (*iv*) management interventions targeted towards females of the core area (“corefemale”).

| 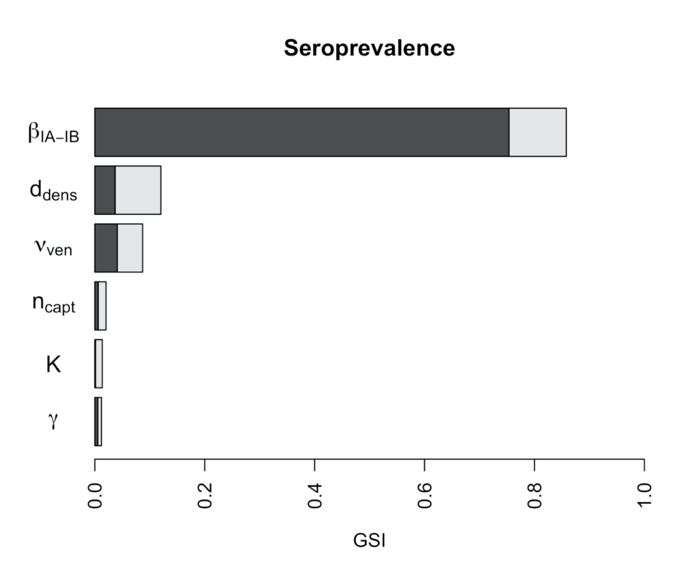 | 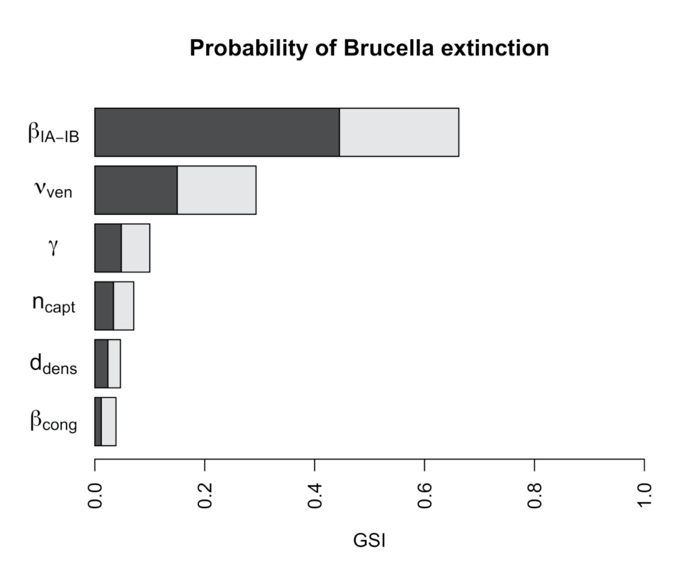 |
| --- | --- |
| 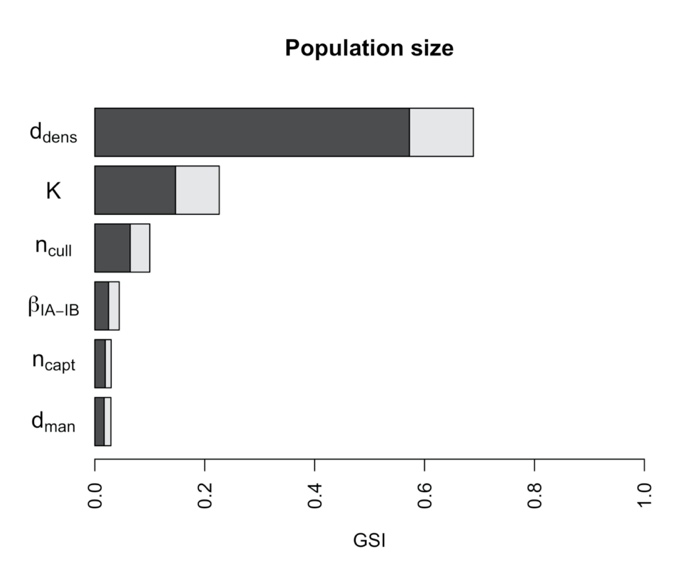 | 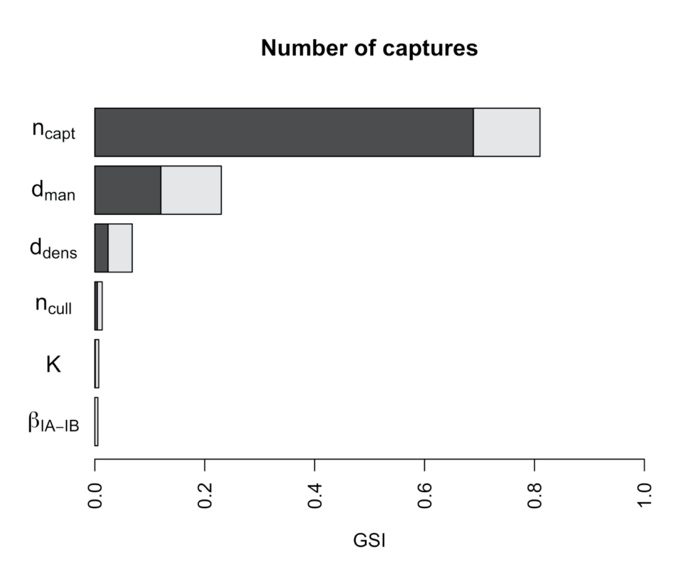 |
| 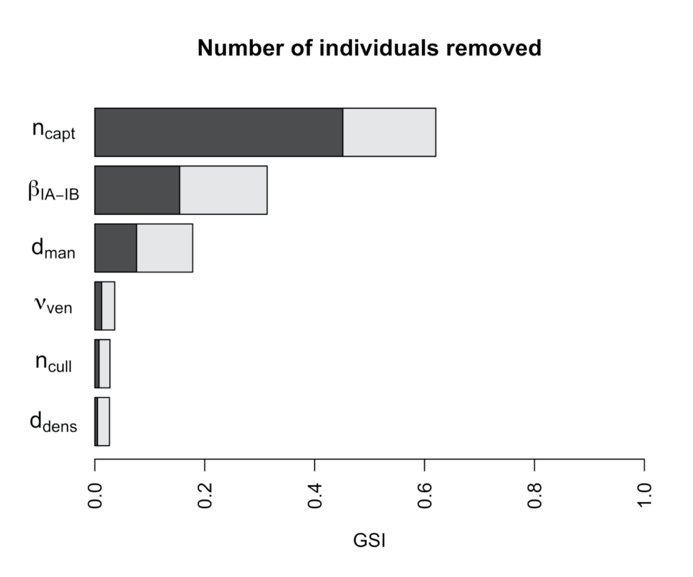 | 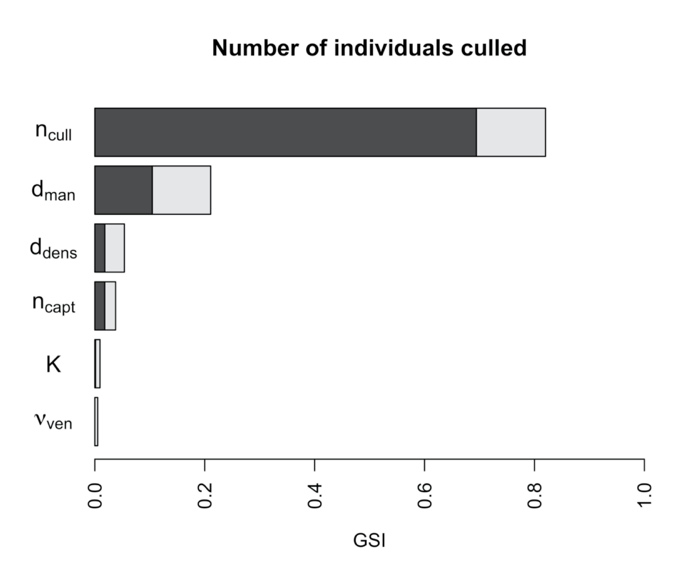 |

**Figure A2: Generalized sensitivity indices for each simulated output in the untargeted scenarios.**

The main sensitivity indices are in dark bars and interaction ones are in pale bars. The total length represents the total sensitivity index. For each output, only the first six parameters were plotted. Outputs were: (*i*) the seroprevalence at the end of the simulations (median and variance); (*ii*) the proportion of simulations where *Brucella* was extinct at the end of the simulation; (*iii*) the population size at the end of the simulations (median and variance); and the numbers of individuals (*iv*) captured, (*v*) removed (during test-and-remove protocol), and (*vi*) culled (without testing) after ten years of simulations (median and variance).

| 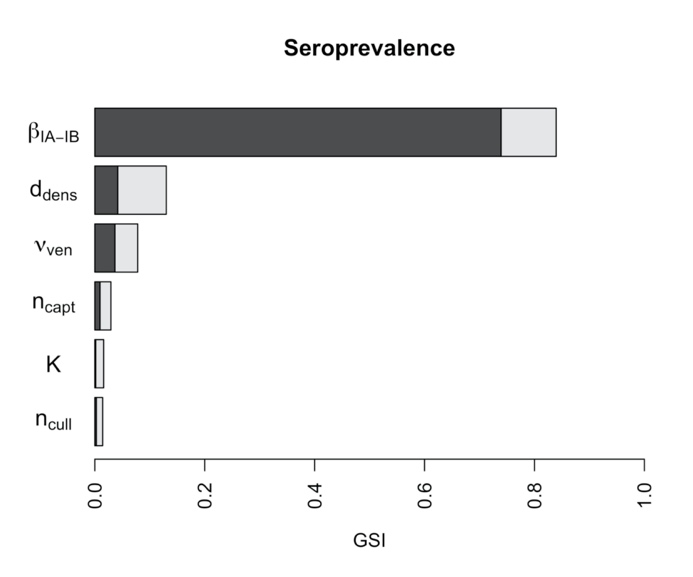 | 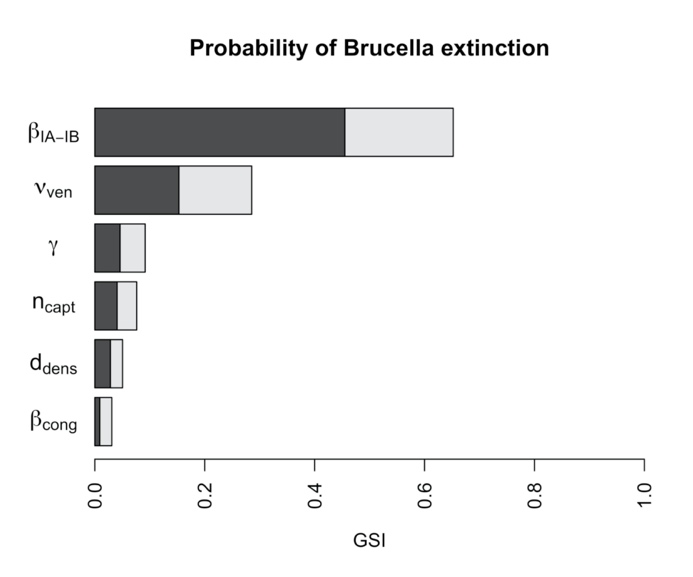 |
| --- | --- |
| 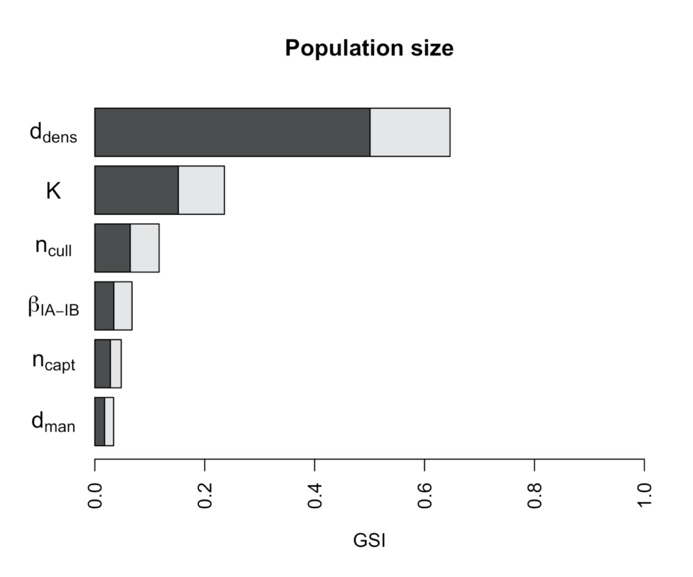 | 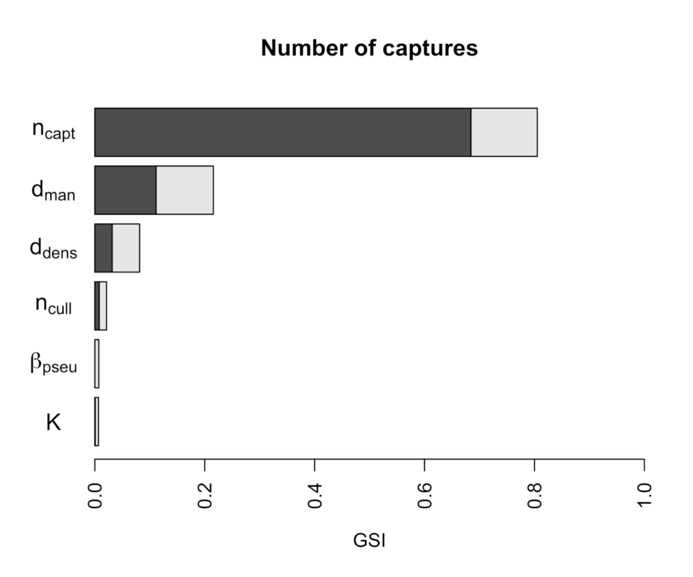 |
| 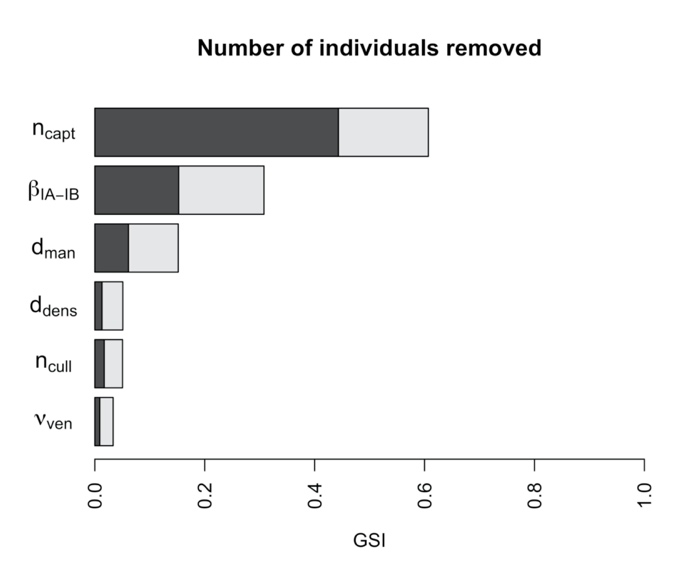 | 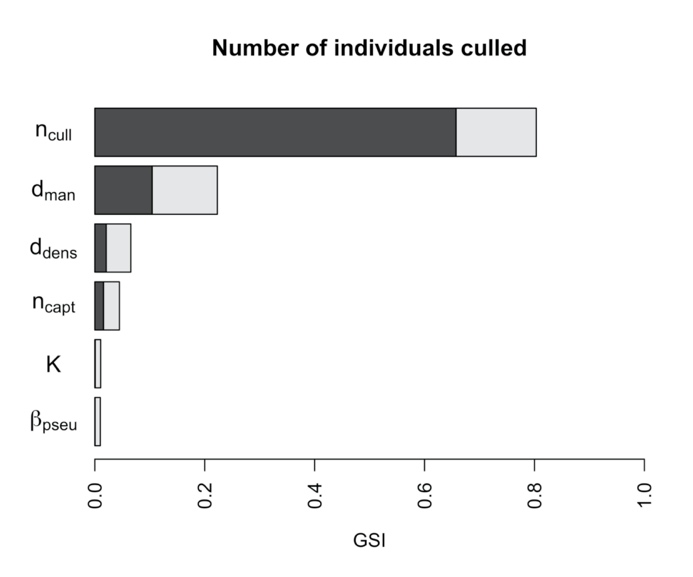 |

**Figure A3: Generalized sensitivity indices for each simulated output in the “core” scenarios.**

The main sensitivity indices are in dark bars and interaction ones are in pale bars. The total length represents the total sensitivity index. For each output, only the first six parameters were plotted. Outputs were: (*i*) the seroprevalence at the end of the simulations (median and variance); (*ii*) the proportion of simulations where *Brucella* was extinct at the end of the simulation; (*iii*) the population size at the end of the simulations (median and variance); and the numbers of individuals (*iv*) captured, (*v*) removed (during test-and-remove protocol), and (*vi*) culled (without testing) after ten years of simulations (median and variance).

| 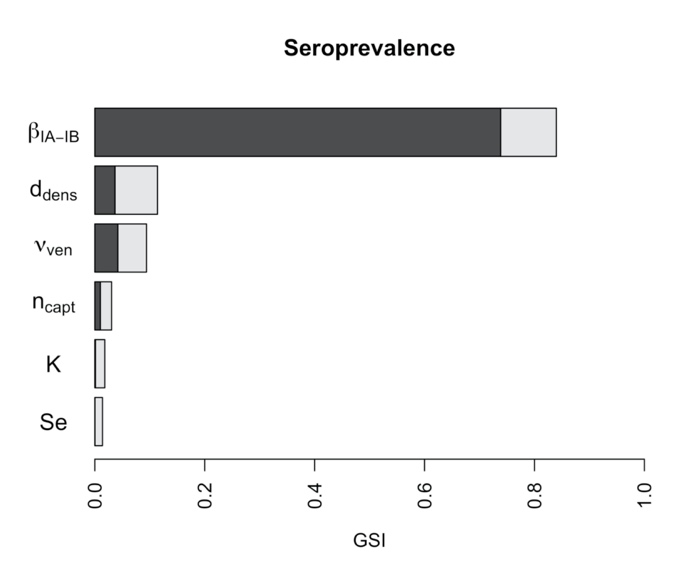 | 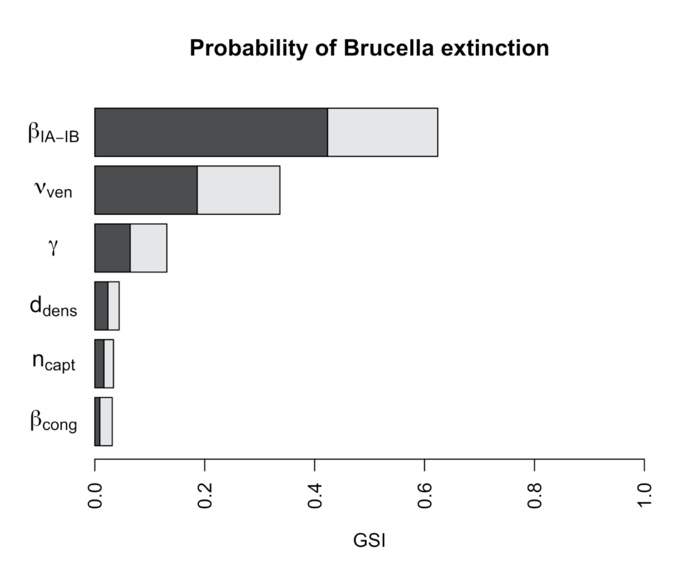 |
| --- | --- |
| 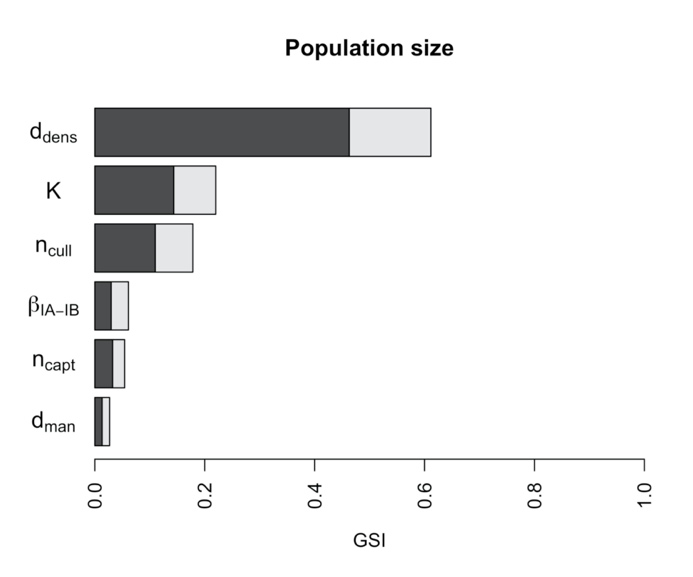 | 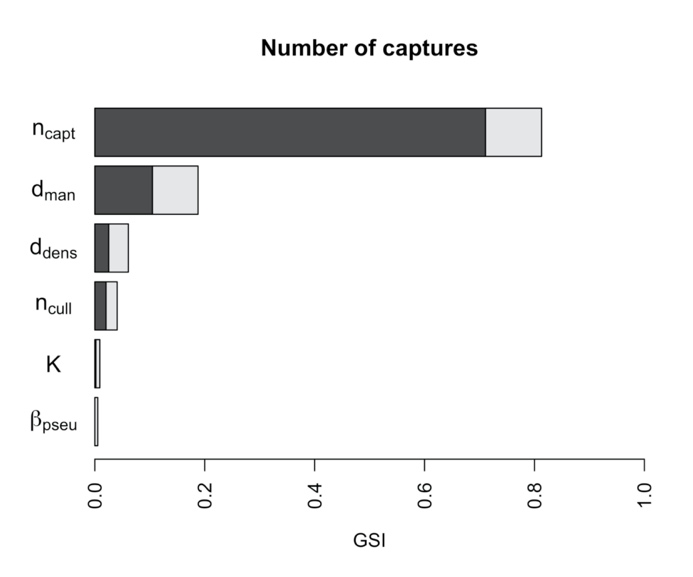 |
| 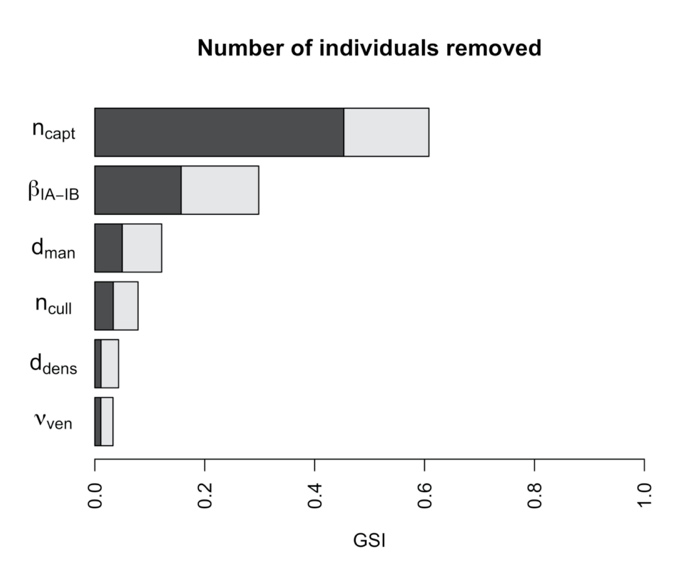 | 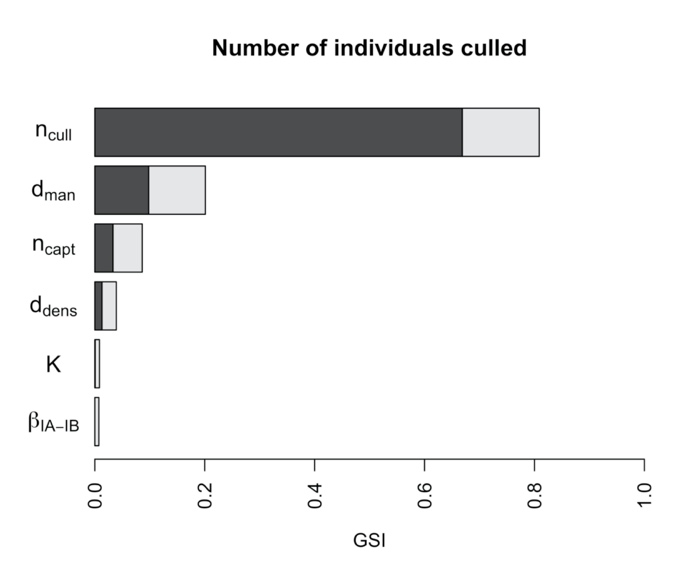 |

**Figure A4: Generalized sensitivity indices for each simulated output in the “female” scenarios.**

The main sensitivity indices are in dark bars and interaction ones are in pale bars. The total length represents the total sensitivity index. For each output, only the first six parameters were plotted. Outputs were: (*i*) the seroprevalence at the end of the simulations (median and variance); (*ii*) the proportion of simulations where *Brucella* was extinct at the end of the simulation; (*iii*) the population size at the end of the simulations (median and variance); and the numbers of individuals (*iv*) captured, (*v*) removed (during test-and-remove protocol), and (*vi*) culled (without testing) after ten years of simulations (median and variance).

| 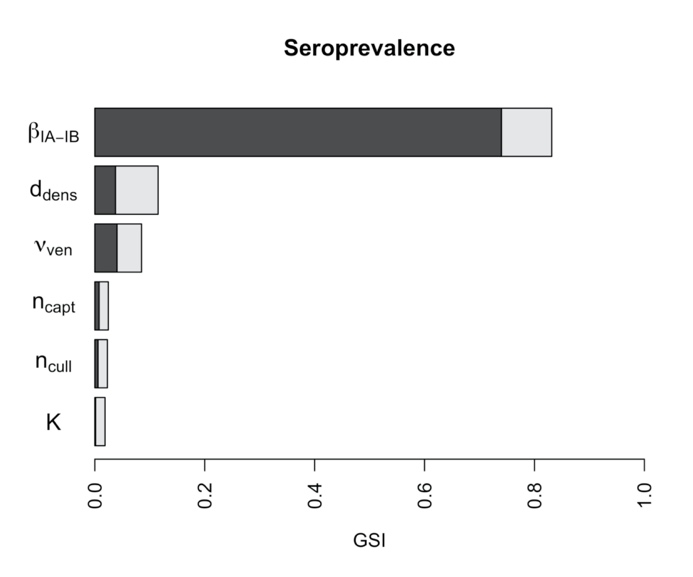 | 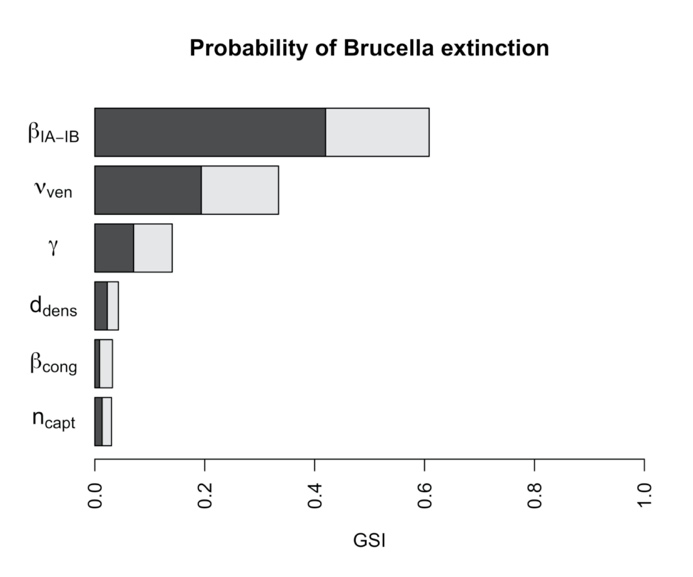 |
| --- | --- |
| 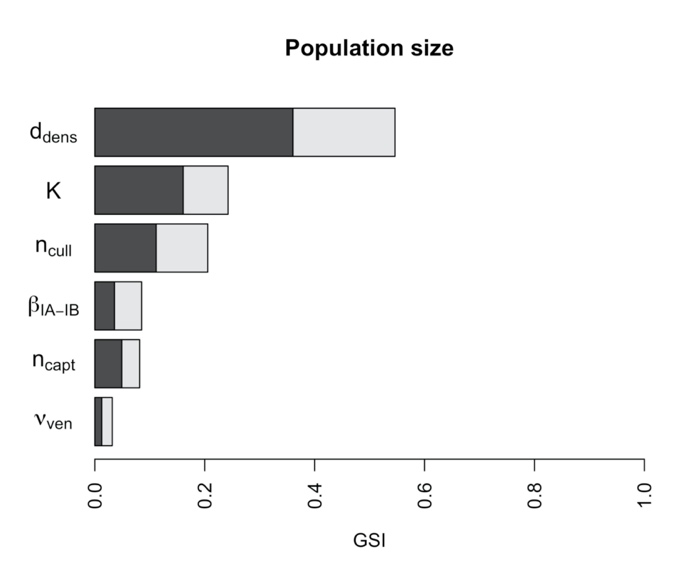 | 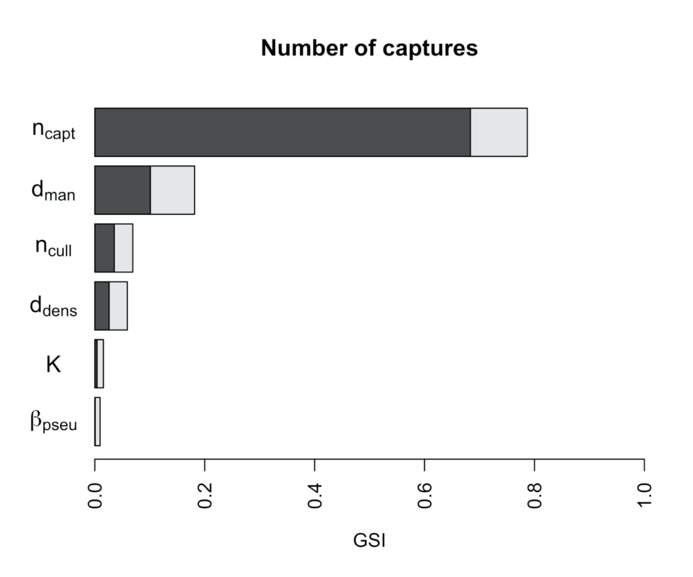 |
| 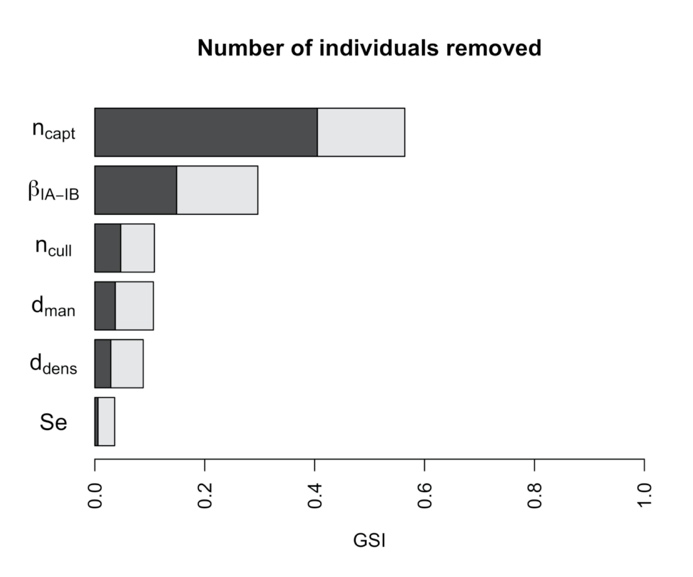 | 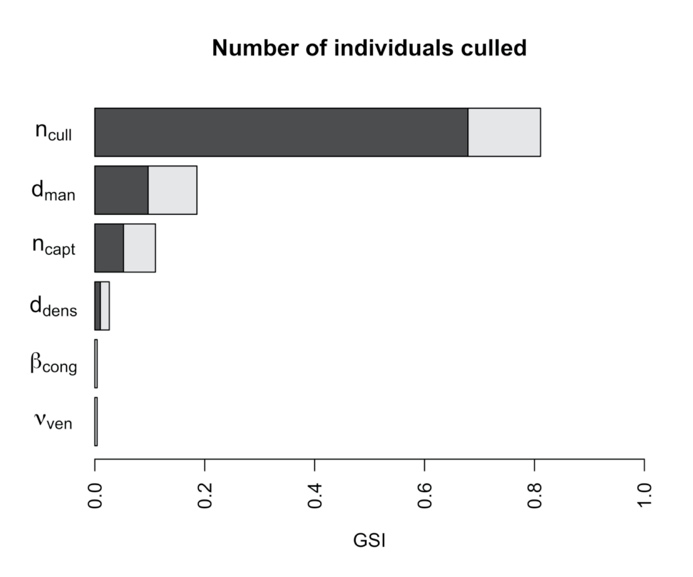 |

**Figure A5: Generalized sensitivity indices for each simulated output in the “corefemale” scenarios.**

The main sensitivity indices are in dark bars and interaction ones are in pale bars. The total length represents the total sensitivity index. Outputs were: (*i*) the seroprevalence at the end of the simulations (median and variance); (*ii*) the proportion of simulations where *Brucella* was extinct at the end of the simulation; (*iii*) the population size at the end of the simulations (median and variance); and the numbers of individuals (*iv*) captured, (*v*) removed (during test-and-remove protocol), and (*vi*) culled (without testing) after ten years of simulations (median and variance).
