# Supplementary material for: Targeted strategies for the management of wildlife diseases: the case of brucellosis in Alpine ibex
Source: Vet Res. 2021 Sep 14;52:116. doi: 10.1186/s13567-021-00984-0 (PMC8439036; doi:10.1186/s13567-021-00984-0)
Supplement: Supplementary file 3 — Additional file 3. Comparison of management scenarios under varying assumptions for the delay in density-dependent responses. This file contains additional figures and tables showing model outputs for different assumptions. [file 13567_2021_984_MOESM3_ESM.docx]

**ADDITIONAL FILE 3: Comparison of management scenarios under varying assumptions for the delay in density-dependent responses**

**1. Short-delay assumption**

| 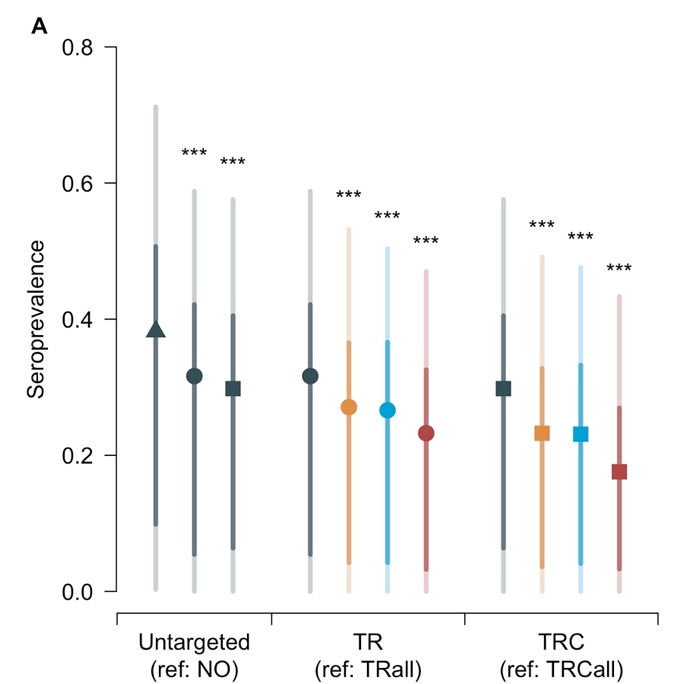 | 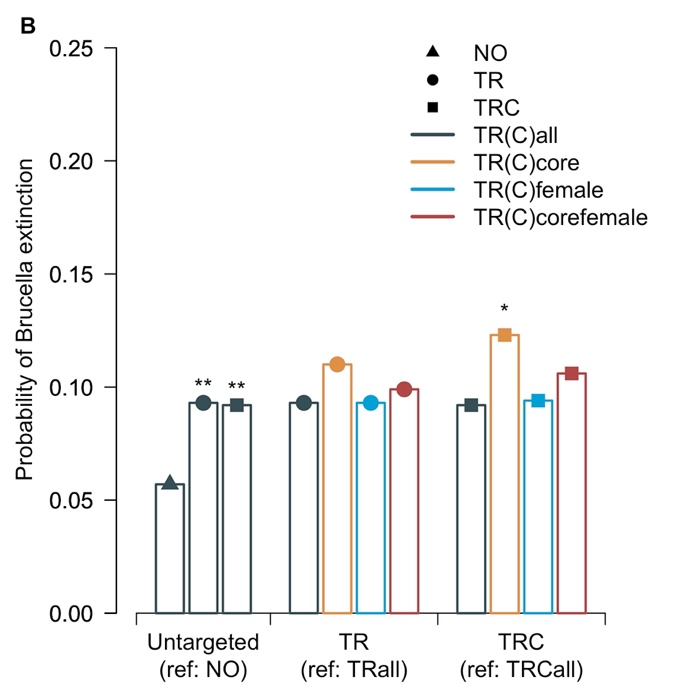 |
| --- | --- |
| 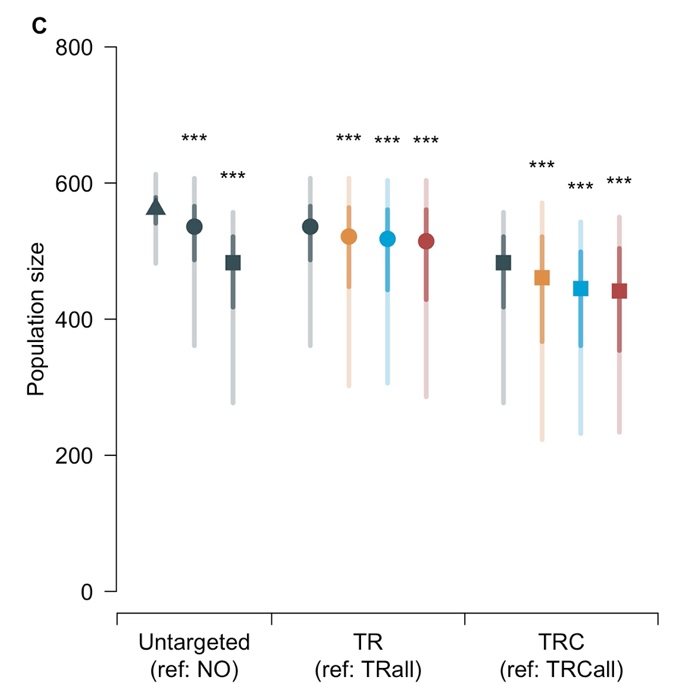 | 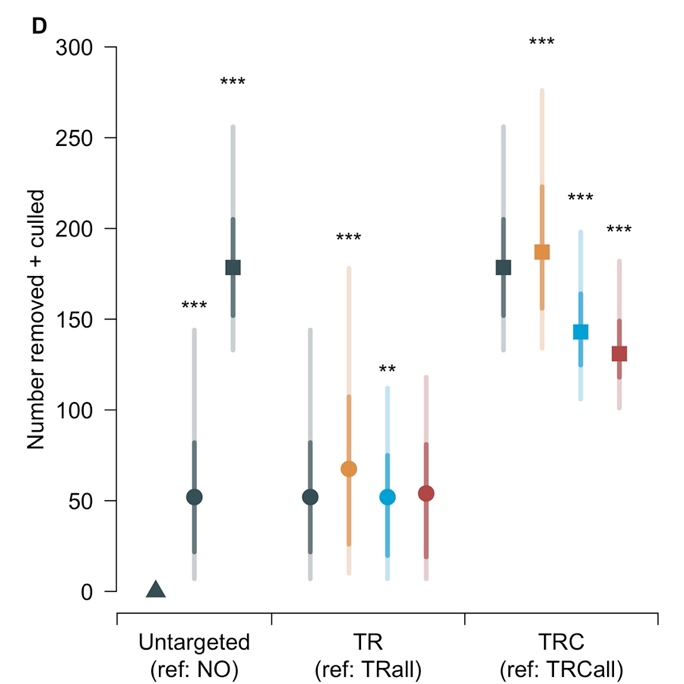 |

**Figure A1: Results of untargeted and targeted management scenarios under the short-delay in density-dependence assumption (increase of reproductive output during first year of simulation).**

(A) Simulated seroprevalence at the end of the simulations; (B) proportion of simulations where *Brucella melitensis* was no longer persistent at the end of the simulations; (C) population size at the end of the simulations; (D) total number of individuals removed and culled over the ten years of simulations. NO: Do Nothing (triangle); TR: Test-and-Remove (points); TRC: TR combined with the culling of unmarked individuals (squares); TR(C)all: untargeted (grey); TR(C)core: targeted towards the core area (orange); TR(C)female: targeted towards females (blue); TR(C)corefemale: targeted towards both females and the core area (dark red). Except for the probability of *Brucella* extinction (single value), central points indicate the median, with 95% and 50% credible intervals indicated by light and dark shaded bars, respectively. Stars above bars indicate the p-values (‘***’: p<0.001; ‘**’: p<0.01; ‘*’: p<0.05) of the chi-squared (B) or Mann-Whitney (A;C-D) tests comparing the result of a given strategy with its reference (indicated in the x-axis). All scenarios (except NO) had an objective of 50 individuals captured each year, and TRC had an additional objective of 20 unmarked individuals culled each year.

**Table A1:** N**umber of individuals managed after ten years of simulations under the short-delay in density-dependence assumption.**

|  |  | **TR scenarios** | | | |
| --- | --- | --- | --- | --- | --- |
|  |  | **TRall (reference)** | **TRcore** | **TRfemale** | **TRcorefemale** |
| **Number (over 10 years)** | Captured | 310 [273-350] | 296 [255-329] $\downarrow$ (*p* < 0.001) | 207 [168-246] $\downarrow$ (*p* < 0.001) | 184 [141-224] $\downarrow$ (*p* < 0.001)^a^ $\downarrow$ (*p* < 0.001)^a^ $\downarrow$ (*p* < 0.001)^b^ |
|  | Removed | 52 [7-144] | 68 [10-178] $\uparrow$ (*p* < 0.001) | 52 [7-112] $\downarrow$ (*p* = 0.006) | 54 [7-118] $\downarrow$ (*p* = 0.310)^a^ $\downarrow$ (*p* < 0.001)^a^ $\downarrow$ (*p* = 0.070)^b^ |
|  |  | **TRC scenarios** | | | |
|  |  | **TRCall (reference)** | **TRCcore** | **TRCfemale** | **TRCcorefemale** |
| **Number (over 10 years)** | Captured | 264 [224-301] | 252 [196-294] $\downarrow$ (*p* < 0.001) | 144 [105-181] $\downarrow$ (*p* < 0.001) | 124 [66-160] $\downarrow$ (*p* < 0.001)^a^ $\downarrow$ (*p* < 0.001)^a^ $\downarrow$ (*p* < 0.001)^b^ |
|  | Removed | 48 [7-126] | 58 [8-142] $\uparrow$ (*p* < 0.001) | 39 [6-89] $\downarrow$ (*p* < 0.001) | 34 [6-81] $\downarrow$ (*p* < 0.001)^a^ $\downarrow$ (*p* < 0.001)^a^ $\downarrow$ (*p* = 0.001)^b^ |
|  | Culled | 131 [114-148] | 131 [113-147] $\downarrow$ (*p* = 0.938) | 107 [86-123] $\downarrow$ (*p* < 0.001) | 99 [77-121] $\downarrow$ (*p* < 0.001)^a^ $\downarrow$ (*p* < 0.001)^a^ $\downarrow$ (*p* < 0.001)^b^ |

TR: Test-and-Remove; TRC: TR combined with the culling of unmarked individuals; TR(C)core: targeted towards the core area of the massif; TR(C)female: targeted towards females; TR(C)corefemale: targeted towards both females and the core area (multilevel). Results are indicated as median [95% credible intervals]. The *p*-values of the Mann-Whitney test for the distributions of the outputs compared to TR(C)all (reference) are indicated in parentheses. All scenarios had an objective of 50 individuals captured per year, and TRC scenarios had an additional objective of 20 unmarked individuals culled per year.

^a^ Reference: TR(C)core

^b^ Reference: TR(C)female

**2. Medium-delay assumption**

| 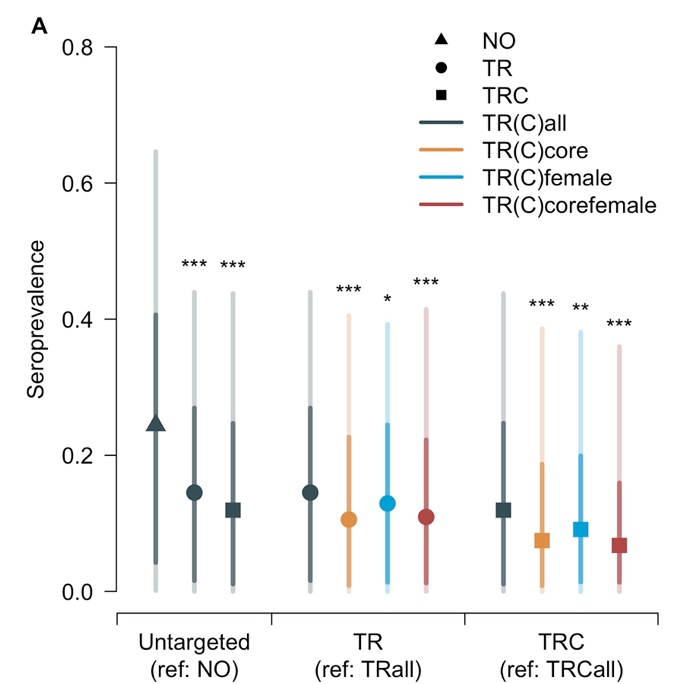 | 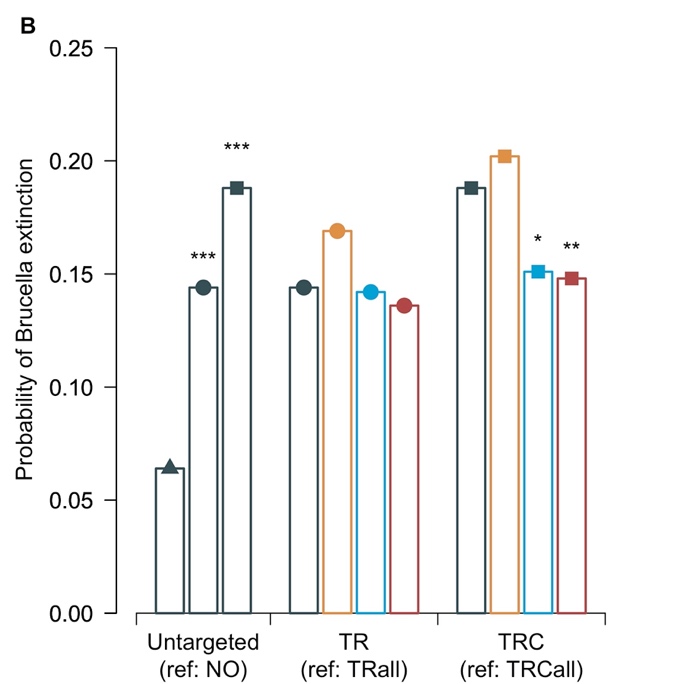 |
| --- | --- |
| 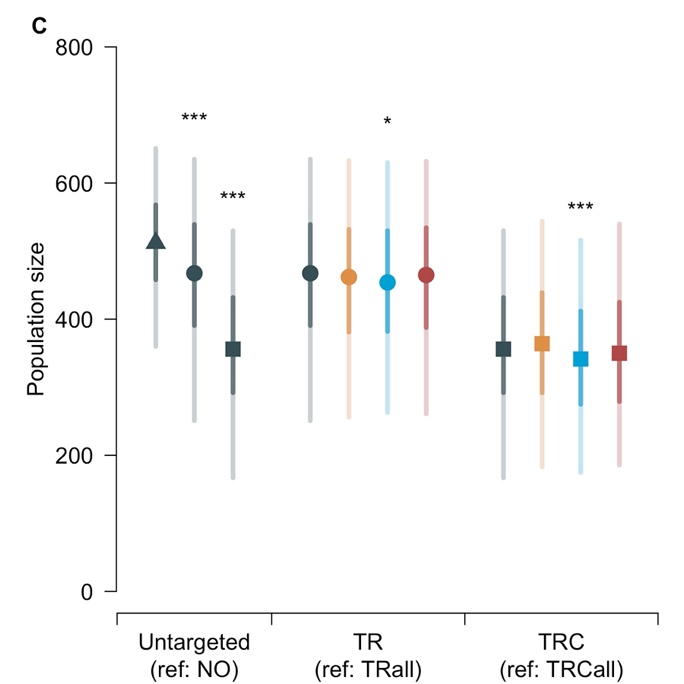 | 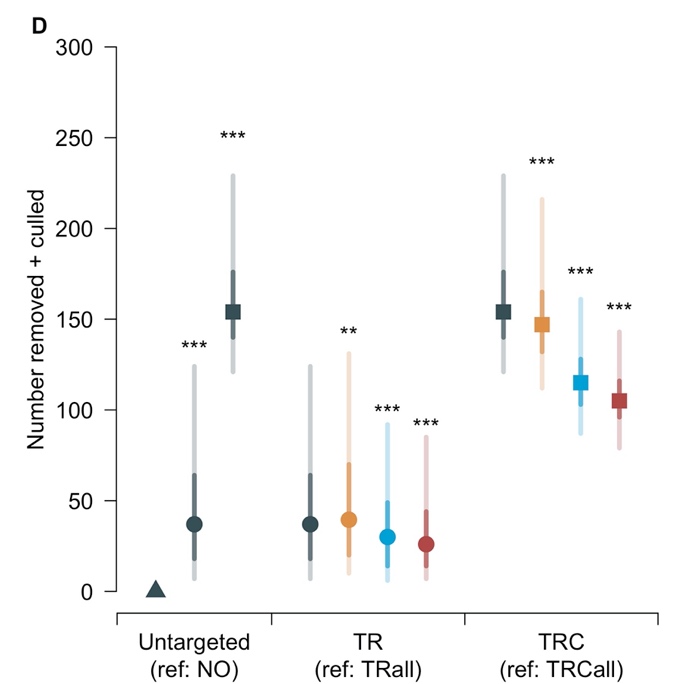 |

**Figure A2: Results of untargeted and targeted management scenarios under the medium-delay in density-dependence assumption (increase of reproductive output during sixth year of simulation).**

(A) Simulated seroprevalence at the end of the simulations; (B) proportion of simulations where *Brucella melitensis* was no longer persistent at the end of the simulations; (C) population size at the end of the simulations; (D) total number of individuals removed and culled over the ten years of simulations. NO: Do Nothing (triangle); TR: Test-and-Remove (points); TRC: TR combined with the culling of unmarked individuals (squares); TR(C)all: untargeted (grey); TR(C)core: targeted towards the core area (orange); TR(C)female: targeted towards females (blue); TR(C)corefemale: targeted towards both females and the core area (dark red). Except for the probability of *Brucella* extinction (single value), central points indicate the median, with 95% and 50% credible intervals indicated by light and dark shaded bars, respectively. Stars above bars indicate the *p*-values (‘***’: *p*<0.001; ‘**’: *p*<0.01; ‘*’: *p*<0.05) of the chi-squared (B) or Mann-Whitney (A;C-D) tests comparing the result of a given strategy with its reference (indicated in the x-axis). All scenarios (except NO) had an objective of 50 individuals captured each year, and TRC had an additional objective of 20 unmarked individuals culled each year.

**Table A2:** N**umber of individuals managed after ten years of simulations under the medium-delay in density-dependence assumption.**

|  |  | **TR scenarios** | | | |
| --- | --- | --- | --- | --- | --- |
|  |  | **TRall (reference)** | **TRcore** | **TRfemale** | **TRcorefemale** |
| **Number (over 10 years)** | Captured | 300 [234-360] | 273 [201-349] $\downarrow$ (*p* < 0.001) | 194 [134-260] $\downarrow$ (*p* < 0.001) | 157 [102-213] $\downarrow$ (*p* < 0.001)^a^ $\downarrow$ (*p* < 0.001)^a^ $\downarrow$ (*p* < 0.001)^b^ |
|  | Removed | 37 [7-124] | 40 [10-131] $\uparrow$ (*p* = 0.007) | 30 [6-92] $\downarrow$ (*p* < 0.001) | 26 [7-85] $\downarrow$ (*p* < 0.001)^a^ $\downarrow$ (*p* < 0.001)^a^ $\downarrow$ (p = 0.016)^b^ |
|  |  | **TRC scenarios** | | | |
|  |  | **TRCall (reference)** | **TRCcore** | **TRCfemale** | **TRCcorefemale** |
| **Number (over 10 years)** | Captured | 241 [169-305] | 201 [127-275] $\downarrow$ (*p* < 0.001) | 121 [71-179] $\downarrow$ (*p* < 0.001) | 88 [42-145] $\downarrow$ (*p* < 0.001)^a^ $\downarrow$ (*p* < 0.001)^a^ $\downarrow$ (*p* < 0.001)^b^ |
|  | Removed | 29 [7-100] | 28 [8-92] $\uparrow$ (*p* = 0.779) | 19 [5-59] $\downarrow$ (*p* < 0.001) | 18 [5-50] $\downarrow$ (*p* < 0.001)^a^ $\downarrow$ (*p* < 0.001)^a^ $\downarrow$ (*p* = 0.002)^b^ |
|  | Culled | 124 [100-149] | 116 [91-140] $\downarrow$ (*p* < 0.001) | 94 [72-117] $\downarrow$ (*p* < 0.001) | 87 [61-107] $\downarrow$ (*p* < 0.001)^a^ $\downarrow$ (*p* < 0.001)^a^ $\downarrow$ (*p* < 0.001)^b^ |

TR: Test-and-Remove; TRC: TR combined with the culling of unmarked individuals; TR(C)core: targeted towards the core area of the massif; TR(C)female: targeted towards females; TR(C)corefemale: targeted towards both females and the core area (multilevel). Results are indicated as median [95% credible intervals]. The *p*-values of the Mann-Whitney test for the distributions of the outputs compared to TR(C)all (reference) are indicated in parentheses. All scenarios had an objective of 50 individuals captured per year, and TRC scenarios had an additional objective of 20 unmarked individuals culled per year.

^a^ Reference: TR(C)core

^b^ Reference: TR(C)female

**3. Long-delay assumption**

| 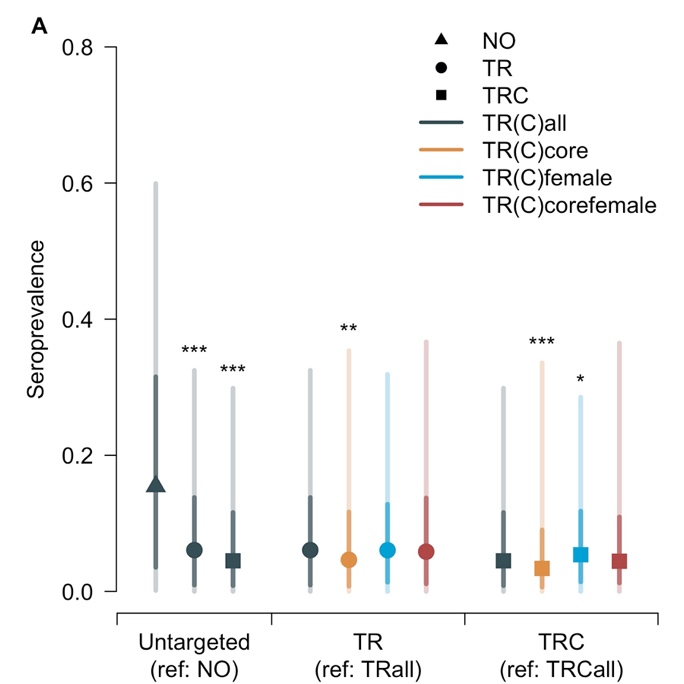 | 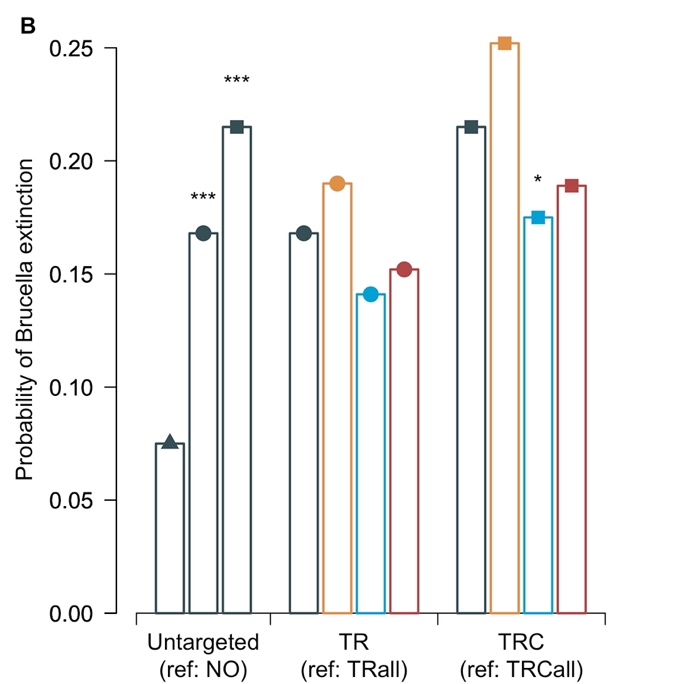 |
| --- | --- |
| 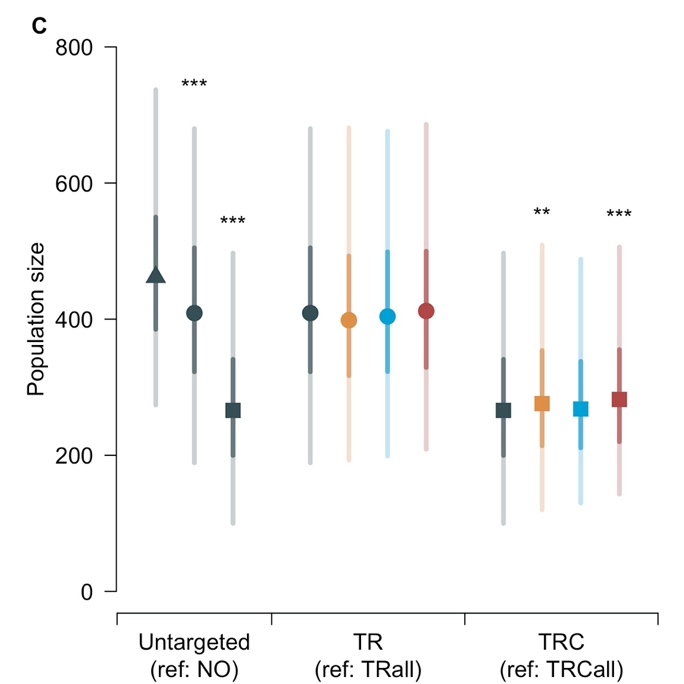 | 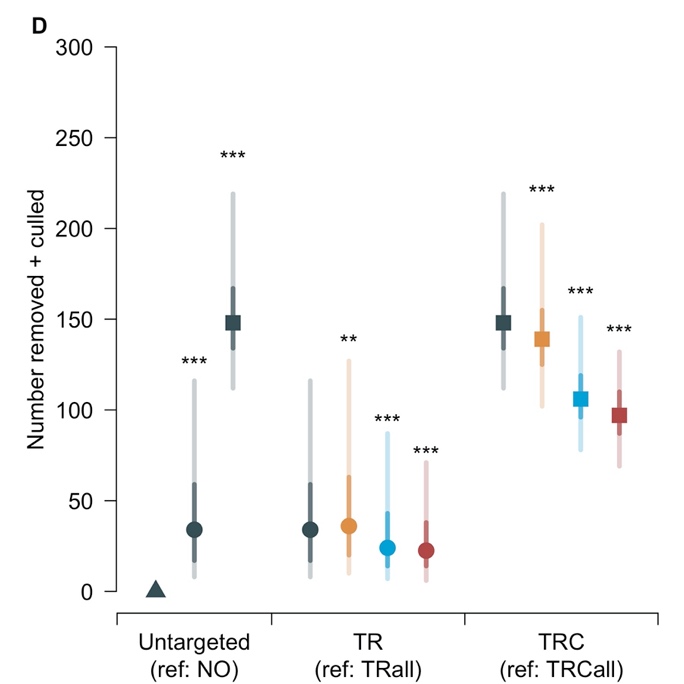 |

**Figure A3: Results of untargeted and targeted management scenarios under the long-delay in density-dependence assumption (no increase of reproductive output during the simulation).**

(A) Simulated seroprevalence at the end of the simulations; (B) proportion of simulations where *Brucella melitensis* was no longer persistent at the end of the simulations; (C) population size at the end of the simulations; (D) total number of individuals removed and culled over the ten years of simulations. NO: Do Nothing (triangle); TR: Test-and-Remove (points); TRC: TR combined with the culling of unmarked individuals (squares); TR(C)all: untargeted (grey); TR(C)core: targeted towards the core area (orange); TR(C)female: targeted towards females (blue); TR(C)corefemale: targeted towards both females and the core area (dark red). Except for the probability of *Brucella* extinction (single value), central points indicate the median, with 95% and 50% credible intervals indicated by light and dark shaded bars, respectively. Stars above bars indicate the p-values (‘***’: *p* < 0.001; ‘**’: *p* < 0.01; ‘*’: *p* < 0.05) of the chi-squared (B) or Mann-Whitney (A;C-D) tests comparing the result of a given strategy with its reference (indicated in the x-axis). All scenarios (except NO) had an objective of 50 individuals captured each year, and TRC had an additional objective of 20 unmarked individuals culled each year.

**Table A3:** N**umber of individuals managed after ten years of simulations under the long-delay in density-dependence assumption.**

|  |  | **TR scenarios** | | | |
| --- | --- | --- | --- | --- | --- |
|  |  | **TRall (reference)** | **TRcore** | **TRfemale** | **TRcorefemale** |
| **Number (over 10 years)** | Captured | 294 [218-360] | 270 [187-348] $\downarrow$ (*p* < 0.001) | 193 [123-266] $\downarrow$ (*p* < 0.001) | 156 [91-231] $\downarrow$ (*p* < 0.001)^a^ $\downarrow$ (*p* < 0.001)^a^ $\downarrow$ (*p* < 0.001)^b^ |
|  | Removed | 34 [8-116] | 36 [10-127] $\uparrow$ (*p* = 0.004) | 24 [7-87] $\downarrow$ (*p* = 0.006) | 22 [6-71] $\downarrow$ (*p* < 0.001)^a^ $\downarrow$ (*p* < 0.001)^a^ $\downarrow$ (*p* = 0.003)^b^ |
|  |  | **TRC scenarios** | | | |
|  |  | **TRCall (reference)** | **TRCcore** | **TRCfemale** | **TRCcorefemale** |
| **Number (over 10 years)** | Captured | 231 [149-303] | 190 [110-280] $\downarrow$ (*p* < 0.001) | 119 [61-191] $\downarrow$ (*p* < 0.001) | 76 [36-146] $\downarrow$ (*p* < 0.001)^a^ $\downarrow$ (*p* < 0.001)^a^ $\downarrow$ (*p* < 0.001)^b^ |
|  | Removed | 26 [6-95] | 26 [8-84] $\uparrow$ (*p* = 0.726) | 17 [5-57] $\downarrow$ (*p* < 0.001) | 16 [6-45] $\downarrow$ (*p* < 0.001)^a^ $\downarrow$ (*p* < 0.001)^a^ $\downarrow$ (*p* < 0.001)^b^ |
|  | Culled | 120 [91-148] | 112 [82-140] $\downarrow$ (*p* < 0.001) | 87 [62-112] $\downarrow$ (*p* < 0.001) | 81 [53-107] $\downarrow$ (*p* < 0.001)^a^ $\downarrow$ (*p* < 0.001)^a^ $\downarrow$ (*p* < 0.001)^b^ |

TR: Test-and-Remove; TRC: TR combined with the culling of unmarked individuals; TR(C)core: targeted towards the core area of the massif; TR(C)female: targeted towards females; TR(C)corefemale: targeted towards both females and the core area (multilevel). Results are indicated as median [95% credible intervals]. The p-values of the Mann-Whitney test for the distributions of the outputs compared to TR(C)all (reference) are indicated in parentheses. All scenarios had an objective of 50 individuals captured per year, and TRC scenarios had an additional objective of 20 unmarked individuals culled per year.

^a^ Reference: TR(C)core

^b^ Reference: TR(C)female

**4. Comparison of the effects of targeting depending on the delay in density-dependent responses**

**Table A4:** **Summary of differences between targeted and untargeted test-and-remove scenarios.**

| **Scenario** | |  | **core vs all** | | |  | **female vs all** | | |  | **corefemale vs all** | | |  | **corefemale vs core** | | |  | **corefemale vs female** | | |
| --- | --- | --- | --- | --- | --- | --- | --- | --- | --- | --- | --- | --- | --- | --- | --- | --- | --- | --- | --- | --- | --- |
| $\boldsymbol{d}_{\boldsymbol{dens}}$ | |  | 0 | 5 | 10 |  | 0 | 5 | 10 |  | 0 | 5 | 10 |  | 0 | 5 | 10 |  | 0 | 5 | 10 |
| Seroprevalence | |  |  | $\downarrow$ |  |  | $\downarrow$ | $\downarrow$ | NS |  | $\downarrow$ | $\downarrow$ | NS |  | $\downarrow$ | NS | $\uparrow$ |  | $\downarrow$ | NS | NS |
| Probability of *Brucella* extinction | |  |  | NS |  |  |  | NS |  |  |  | NS |  |  | NS | $\downarrow$ | $\downarrow$ |  |  | NS |  |
| Population size | |  | $\downarrow$ | NS | NS |  | $\downarrow$ | NS | NS |  | $\downarrow$ | NS | NS |  | $\downarrow$ | NS | NS |  |  | NS |  |
| Number (over 10 years) | Captured |  |  | $\downarrow$ |  |  |  | $\downarrow$ |  |  |  | $\downarrow$ |  |  |  | $\downarrow$ |  |  |  | $\downarrow$ |  |
|  | Removed |  |  | $\uparrow$ |  |  |  | $\downarrow$ |  |  | NS | $\downarrow$ | $\downarrow$ |  |  | $\downarrow$ |  |  | NS | $\downarrow$ | $\downarrow$ |

TR: Test-and-Remove; TRall: untargeted; TRcore: targeted towards the core area; TRfemale: targeted towards females; TRcorefemale: targeted towards both females and the core area. Arrows indicate significant (p<0.05) increase ($\uparrow$) or decrease ($\downarrow$) of model outputs as indicated by the chi-squared (probability of *Brucella* extinction) or Mann-Whitney (all other outputs) tests comparing the results between two scenarios. ‘NS’ indicates the absence of significant differences. Output values are detailed in Figures A1-3 and Tables A1-3. The delay in density-dependent responses, $d_{dens}$, takes different values according to the assumption (“short-delay” assumption: $d_{dens}=0$ years; “medium-delay” assumption: $d_{dens}=5$ years; “long-delay” assumption: $d_{dens}=10$ years).

**Table A5:** **Summary of differences between targeted and untargeted test-and-remove scenarios combined with culling.**

| **Scenario** | |  | **core vs all** | | |  | **female vs all** | | |  | **corefemale vs all** | | |  | **corefemale vs core** | | |  | **corefemale vs female** | | |
| --- | --- | --- | --- | --- | --- | --- | --- | --- | --- | --- | --- | --- | --- | --- | --- | --- | --- | --- | --- | --- | --- |
| $\boldsymbol{d}_{\boldsymbol{dens}}$ | |  | 0 | 5 | 10 |  | 0 | 5 | 10 |  | 0 | 5 | 10 |  | 0 | 5 | 10 |  | 0 | 5 | 10 |
| Seroprevalence | |  |  | $\downarrow$ |  |  | $\downarrow$ | $\downarrow$ | $\uparrow$ |  | $\downarrow$ | $\downarrow$ | NS |  | $\downarrow$ | NS | $\uparrow$ |  | $\downarrow$ | $\downarrow$ | NS |
| Probability of *Brucella* extinction | |  | $\uparrow$ | NS | NS |  | NS | $\downarrow$ | $\downarrow$ |  | NS | $\downarrow$ | NS |  | NS | $\downarrow$ | $\downarrow$ |  |  | NS |  |
| Population size | |  | $\downarrow$ | NS | $\uparrow$ |  | $\downarrow$ | $\downarrow$ | NS |  | $\downarrow$ | NS | $\uparrow$ |  | $\downarrow$ | $\downarrow$ | NS |  | NS | NS | $\uparrow$ |
| Number (over 10 years) | Captured |  |  | $\downarrow$ |  |  |  | $\downarrow$ |  |  |  | $\downarrow$ |  |  |  | $\downarrow$ |  |  |  | $\downarrow$ |  |
|  | Removed |  | $\uparrow$ | NS | NS |  |  | $\downarrow$ |  |  |  | $\downarrow$ |  |  |  | $\downarrow$ |  |  |  | $\downarrow$ |  |
|  | Culled |  | NS | $\downarrow$ | $\downarrow$ |  |  | $\downarrow$ |  |  |  | $\downarrow$ |  |  |  | $\downarrow$ |  |  |  | $\downarrow$ |  |
|  | Removed + culled |  | $\uparrow$ | $\downarrow$ | $\downarrow$ |  |  | $\downarrow$ |  |  |  | $\downarrow$ |  |  |  | $\downarrow$ |  |  |  | $\downarrow$ |  |

TRC: Test-and-Remove combined with the culling of unmarked individuals; TRCall: untargeted; TRCcore: targeted towards the core area; TRCfemale: targeted towards females; TRCcorefemale: targeted towards both females and the core area. Arrows indicate significant (p<0.05) increase ($\uparrow$) or decrease ($\downarrow$) of model outputs as indicated by the chi-squared (probability of *Brucella* extinction) or Mann-Whitney (all other outputs) tests comparing the results between two scenarios. “NS” indicates the absence of significant differences. Output values are detailed in Figures A1-3 and Tables A1-3. The delay in density-dependent responses, $d_{dens}$, takes different values according to the assumption (“short-delay” assumption: $d_{dens}=0$ years; “medium-delay” assumption: $d_{dens}=5$ years; “long-delay” assumption: $d_{dens}=10$ years).
