# Supplementary material for: Targeted strategies for the management of wildlife diseases: the case of brucellosis in Alpine ibex
Source: Vet Res. 2021 Sep 14;52:116. doi: 10.1186/s13567-021-00984-0 (PMC8439036; doi:10.1186/s13567-021-00984-0)
Supplement: Supplementary file 4 — Additional file 4. Test-and-vaccinate-or-remove strategy. [30, 35, 36, 40, 74–80] This file describes the methods, the results and the conclusions for an additional management scenario. [file 13567_2021_984_MOESM4_ESM.docx]

**ADDITIONAL FILE 4: Test-and-Vaccinate-or-Remove strategy**

**1. Materials and Methods**

**1.1. Management strategy**

We explored an additional untargeted strategy called Test-and-Vaccinate-or-Remove (“TVR”), i.e., serological testing of captured individuals followed by removal (euthanasia) of seropositive individuals among them (Test-and-Remove: “TR”), combined with the vaccination of seronegative individuals that are marked and released. Because only unmarked individuals were targeted for capture, marked individuals captured before 2018 (~40% of the population [35]) were not vaccinated and vaccinated (marked) individuals could not be booster-vaccinated to extend the protection of the vaccine.

Based on the results obtained for the untargeted TVR strategy compared to TR alone and to TR combined with the culling of unmarked individuals (“TRC”), we decided not to explore its targeted counterparts.

**1.2. *Brucella melitensis* vaccine**

The live *B. melitensis* Rev.1 strain is considered the only vaccine available for *B. melitensis* management in small domestic ruminants [74]. Indeed, the efficacy of killed vaccines is not high enough [75] and only live vaccines are considered suitable for brucellosis management [74]. Moreover, alternative live vaccines are less effective than Rev. 1 in domestic ruminants [74]. This vaccine was considered as a potential tool for the management of *B. melitensis* in Alpine ibex [30, 36, 40], but as all live vaccines it faces several limitations.

A previous study assessed the innocuousness of this vaccine in non-pregnant adult Alpine ibex. None of the vaccinated animals developed lesions or clinical signs attributable to the vaccine [40]. However, the existence of vaccine-induced abortions in pregnant females, reported in domestic ruminants [76], was not assessed in this study. More importantly, vaccinated individuals shed the vaccine strain and non-vaccinated contact individuals developed strong serological responses [40]. This represents a serious limitation to the use of the vaccine in the field, as the serological responses of individuals exposed to the vaccine strain cannot be distinguished from those of naturally-infected animals [30, 40]. Finally, although the efficacy of the vaccine against *B. melitensis* in Alpine ibex remains to be determined, it does not confer 100% protection in domestic ruminants [77].

Despite all these limitations, we simulated an optimal vaccine to assess if it could improve the efficacy of management strategies, keeping in mind that those limitations could restrain the use of vaccination if this strategy turned out to be promising according to the results of the model. Thus, we assumed that: (*i*) the vaccine was perfectly safe, including in pregnant females; (*ii*) vaccinated individuals did not shed the vaccine strain and transmit it to other individuals; (*iii*) the efficacy of vaccination was 100%, meaning that susceptible individuals that received vaccination could not become infected as long as vaccination conferred protection.

We also assumed that the duration of protective immunity was 4.5 years, based on experiments on small domestic ruminants [78], and that vaccination of infected individuals that were mistakenly detected as seronegative during capture had no effect [76].

**2. Results**

**2.1 Management interventions *vs* doing nothing**

All management strategies (TRall, TVRall and TRCall) showed increased sanitary benefits (significantly higher probabilities of extinction and significantly lower seroprevalence) associated with increased population costs (significantly lower population sizes) compared to the reference scenario NO (Figure A1).

**2.2 Comparison between TR, TVR and TRC**

The total number of individuals captured and removed was significantly lower for TRCall compared to TRall and TVRall because culling unmarked individuals each year reduced the number of unmarked individuals available for capture (Tables A1-2).

TVRall was associated with significantly lower seroprevalence compared to TRall, for similar number of captures and individuals removed (Tables A1-2), because of the additional benefits of vaccination. However, no significant differences were observed between the probabilities of *B. melitensis* extinction between TVRall and TRall (Table A1).

Except for the short-delay assumption, TRCall was associated with significantly lower seroprevalence and significantly higher probabilities of extinction compared to TRall (Table A1). However, these increased sanitary benefits were associated with a significantly lower population size at the end of the simulations compared to TRall because of the additional culling of unmarked individuals (Table A1 and Figure A1).

Finally, there were no significant differences between the seroprevalence and the probability of *B. melitensis* extinction between TVRall and TRCall (Table A1).

**Table A1:** **Summary of differences between management scenarios under varying assumptions for the delay in density-dependent responses.**

| **Scenario** | |  | **TVRall vs TRall** | | |  | **TRCall vs TRall** | | |  | **TRCall vs TVRall** | | |
| --- | --- | --- | --- | --- | --- | --- | --- | --- | --- | --- | --- | --- | --- |
| $\boldsymbol{d}_{\boldsymbol{dens}}$ | |  | 0 | 5 | 10 |  | 0 | 5 | 10 |  | 0 | 5 | 10 |
| Seroprevalence | |  |  | $\downarrow$ |  |  | NS | $\downarrow$ | $\downarrow$ |  |  | NS |  |
| Probability of *Brucella* extinction | |  |  | NS |  |  | NS | $\uparrow$ | $\uparrow$ |  |  | NS |  |
| Population size | |  |  | NS |  |  |  | $\downarrow$ |  |  |  | $\downarrow$ |  |
| Number (over 10 years) | Captured |  |  | NS |  |  |  | $\downarrow$ |  |  |  | $\downarrow$ |  |
|  | Removed |  |  | NS |  |  |  | $\downarrow$ |  |  |  | $\downarrow$ |  |
|  | Removed + culled |  |  | NS |  |  |  | $\uparrow$ |  |  |  | $\uparrow$ |  |

TRall: untargeted Test-and-Remove; TVRall: untargeted Test-and-Vaccinate-or-Remove; TRCall: untargeted TR combined with culling of unmarked individuals. Arrows indicate significant (*p* < 0.05) increase ($\uparrow$) or decrease ($\downarrow$) of model outputs as indicated by the chi-squared (probability of *Brucella* extinction) or Mann-Whitney (all other outputs) tests comparing the results between two scenarios. “NS” indicates the absence of significant differences. Output values are detailed in Figure A1 and Table A2. The delay in density-dependent responses, $d_{dens}$, takes different values according to the assumption.

| 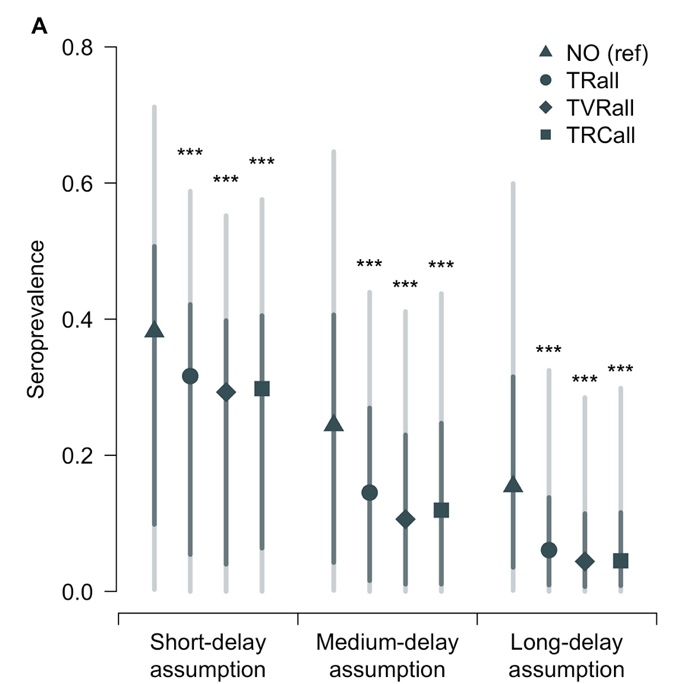 | 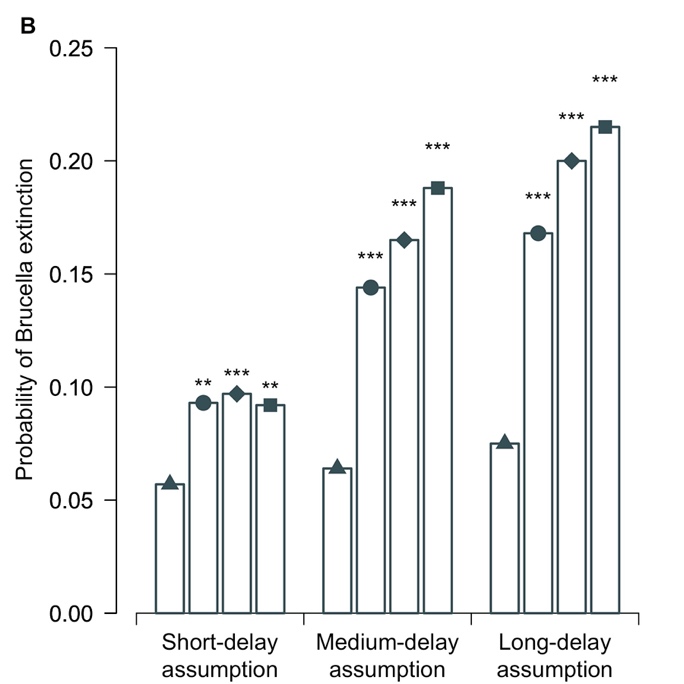 |
| --- | --- |
| 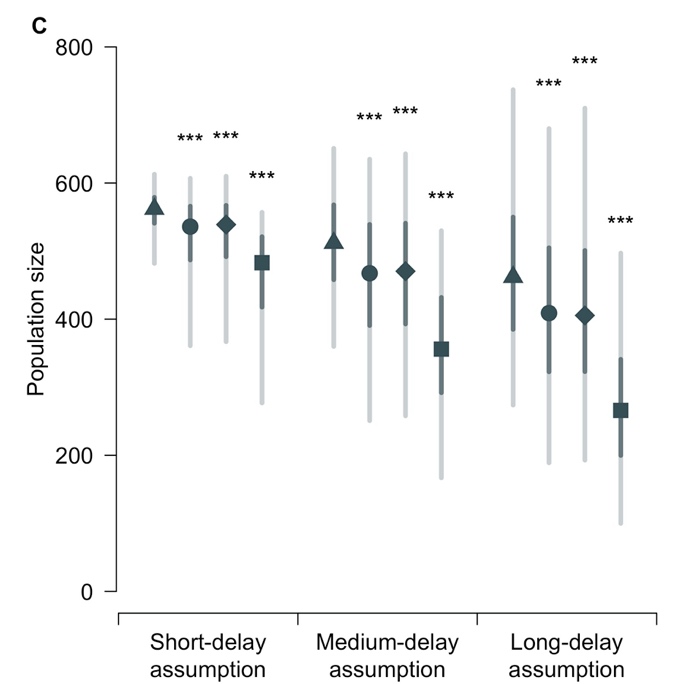 | 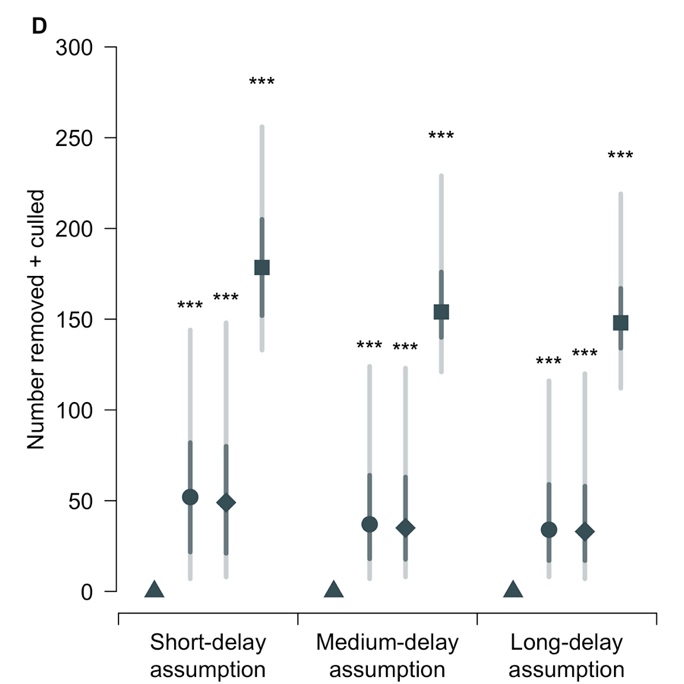 |

**Figure A1: Results of untargeted management scenarios under varying assumptions for the delay in density-dependent responses.**

(A) Simulated seroprevalence at the end of the simulations; (B) proportion of simulations where *Brucella melitensis* was no longer persistent at the end of the simulations; (C) population size at the end of the simulations; (D) total number of individuals removed and culled over the ten years of simulations. NO: Do Nothing (triangle); TRall: untargeted Test-and-Remove (points); TVRall: untargeted Test-and-Vaccinate-or-Remove (diamond); TRCall: untargeted TR combined with the culling of unmarked individuals (squares). Except for the probability of *Brucella* extinction (single value), central points indicate the median, with 95% and 50% credible intervals indicated by light and dark shaded bars, respectively. Stars above bars indicate the p-values (‘***’: *p* < 0.001; ‘**’: *p* < 0.01; ‘*’: *p* < 0.05) of the chi-squared (B) or Mann-Whitney (A;C;D) tests comparing the result of a given strategy with its reference (NO). All scenarios (except NO) had an objective of 50 individuals captured each year, and TRC had an additional objective of 20 unmarked individuals culled each year.

**Table A2: Number of individuals managed after ten years of simulations under varying assumptions for the delay in density-dependent responses.**

|  |  | **Short-delay assumption** | | | |
| --- | --- | --- | --- | --- | --- |
|  |  | **NO (reference)** | **TRall** | **TVRall** | **TRCall** |
| **Number (over 10 years)** | Captured | 0 | 310 [273-350] $\uparrow$ (*p* < 0.001) | 310 [270-348] $\uparrow$ (*p* < 0.001)^a^ $\uparrow$ (*p* = 0.634)^a^ | 264 [224-301] $\uparrow$ (*p* < 0.001)^a^ $\downarrow$ (*p* < 0.001)^a^ $\downarrow$ (*p* < 0.001)^b^ |
|  | Removed | 0 | 52 [7-144] $\uparrow$ (*p* < 0.001) | 49 [8-148] $\uparrow$ (*p* < 0.001)^a^ $\uparrow$ (*p* = 0.402)^a^ | 48 [7-126] $\uparrow$ (*p* < 0.001)^a^ $\downarrow$ (*p* = 0.002)^a^ $\downarrow$ (*p* = 0.019)^b^ |
|  | Culled | 0 | 0 | 0 | 131 [114-148] $\uparrow$ (*p* < 0.001)^a^ |
|  |  | **Medium-delay assumption** | | | |
|  |  | **NO (reference)** | **TRall** | **TVRall** | **TRCall** |
| **Number (over 10 years)** | Captured | 0 | 300 [234-360] $\uparrow$ (*p* < 0.001) | 301 [230-361] $\uparrow$ (*p* < 0.001)^a^ $\uparrow$ (*p* = 0.973)^a^ | 241 [169-305] $\uparrow$ (*p* < 0.001)^a^ $\downarrow$ (*p* < 0.001)^a^ $\downarrow$ (*p* < 0.001)^b^ |
|  | Removed | 0 | 37 [7-124] $\uparrow$ (*p* < 0.001) | 35 [8-123] $\uparrow$ (*p* < 0.001)^a^ $\uparrow$ (*p* = 0.434)^a^ | 29 [7-100] $\uparrow$ (*p* < 0.001)^a^ $\downarrow$ (*p* < 0.001)^a^ $\downarrow$ (*p* = 0.001)^b^ |
|  | Culled | 0 | 0 | 0 | 124 [100-149] $\uparrow$ (*p* < 0.001)^a^ |
|  |  | **Long-delay assumption** | | | |
|  |  | **NO (reference)** | **TRall** | **TVRall** | **TRCall** |
| **Number (over 10 years)** | Captured | 0 | 204 [218-360] $\uparrow$ (*p* < 0.001) | 293 [219-362] $\uparrow$ (*p* < 0.001) ^a^ $\uparrow$ (*p* = 0.905)^a^ | 231 [149-303] $\uparrow$ (*p* < 0.001)^a^ $\downarrow$ (*p* < 0.001)^a^ $\downarrow$ (*p* < 0.001)^b^ |
|  | Removed | 0 | 34 [8-116] $\uparrow$ (*p* < 0.001) | 33 [7-120] $\uparrow$ (*p* < 0.001) ^a^ $\uparrow$ (*p* = 0.706)^a^ | 26 [6-95] $\uparrow$ (*p* < 0.001)^a^ $\downarrow$ (*p* < 0.001)^a^ $\downarrow$ (*p* = 0.001)^b^ |
|  | Culled | 0 | 0 | 0 | 120 [91-148] $\uparrow$ (*p* < 0.001)^a^ |

NO: Do Nothing; TRall: untargeted Test-and-Remove; TVRall: untargeted Test-and-Remove-or-Vaccinate; TRCall: untargeted TR combined with the culling of unmarked individuals. Results are indicated as median [95% credible intervals]. The p-values of the Mann-Whitney test for the distributions of the outputs compared to NO (reference) are indicated in parentheses. All scenarios had an objective of 50 individuals captured per year, and TRC scenarios had an additional objective of 20 unmarked individuals culled per year.

^a^ Reference: TRall

^b^ Reference: TVRall

**3. Discussion**

The addition of vaccination compared to test-and-remove alone improved the efficacy in reducing seroprevalence but not brucellosis persistence, all else being equal (Figure A1 and Table A1). However, it did not improve the sanitary benefits compared to test-and-remove combined with culling of unmarked individuals (Table A1).

Although we used a 100% efficacy of the vaccine in our study, this result could be explained by a relatively small coverage of vaccination in the population. Indeed, around 250 animals detected as seronegative were vaccinated in ten years when the objective was 50 captures each year (Table A2), but it represented only a very small proportion of the population each year (only 50 animals captured, and, among them, only seronegative individuals can be vaccinated). As for TR, we could also expect a better efficacy of the TVR strategy if the number of captures could be increased [36, 79]. However, the number of captures tested in our study was considered as a realistic and achievable target.

Moreover, we assumed that protective immunity induced by the vaccine was not lifelong. Therefore, vaccinated individuals who lost their protection after some time returned to the susceptible state and could get the infection. Because vaccinated animals were marked and no recapture were implemented in our scenarios, these individuals who lost their protection could neither be vaccinated again nor be removed if they got infected, which probably hampered the efficacy of vaccination. In bison in the Greater Yellowstone Area, the most effective vaccination strategy evaluated by modelling allowed for repeated vaccinations of animals, thus extending the duration of vaccine protection [80]. Although the duration of vaccine protection may be longer than the 4.5 years that were used here [78], there are currently no data to validate or invalidate this in ibex. If the vaccine should be used in the studied population, it could therefore be recommended to recapture vaccinated individuals after some time to extend the duration of vaccine protection (but as a result the proportion of unmarked individuals within captures would decrease if the total number of captures, i.e., 50 per year, is not increased). A scenario where recaptures are allowed in our model could be evaluated to confirm or not the additional benefit of such a strategy.

However, the use of the currently available *B. melitensis* vaccine faces many serious limitations [30, 40]: it is unlikely that the vaccine confers 100% protection in ibex (as in domestic ruminants), vaccine-induced abortions in pregnant females cannot be excluded, and vaccinated individuals have the ability to shed the vaccine strain and as a result to develop strong serological responses in non-vaccinated individuals that cannot be distinguished from naturally-infected animals. Given all these limitations and the unpromising results obtained in our study, TVR strategies were not further explored [30].
